# Development of robust targeted proteomics assays for cerebrospinal fluid biomarkers in multiple sclerosis

**DOI:** 10.1186/s12014-020-09296-5

**Published:** 2020-09-18

**Authors:** Astrid Guldbrandsen, Ragnhild Reehorst Lereim, Mari Jacobsen, Hilde Garberg, Ann Cathrine Kroksveen, Harald Barsnes, Frode S. Berven

**Affiliations:** 1 Proteomics Unit, PROBE, Department of Biomedicine, University of Bergen, Bergen, Norway; 2 Computational Biology Unit, CBU, Department of Informatics, University of Bergen, Bergen, Norway; 3 Biobank Haukeland, Haukeland University Hospital, Bergen, Norway

**Keywords:** Proteomics, Parallel reaction monitoring, Cerebrospinal fluid, Multiple sclerosis, Biomarker, Assay development, Neurological diseases

## Abstract

**Background:**

Verification of cerebrospinal fluid (CSF) biomarkers for multiple sclerosis and other neurological diseases is a major challenge due to a large number of candidates, limited sample material availability, disease and biological heterogeneity, and the lack of standardized assays. Furthermore, verification studies are often based on a low number of proteins from a single discovery experiment in medium-sized cohorts, where antibodies and surrogate peptides may differ, thus only providing an indication of proteins affected by the disease and not revealing the bigger picture or concluding on the validity of the markers. We here present a standard approach for locating promising biomarker candidates based on existing knowledge, resulting in high-quality assays covering the main biological processes affected by multiple sclerosis for comparable measurements over time.

**Methods:**

Biomarker candidates were located in CSF-PR (proteomics.uib.no/csf-pr), and further filtered based on estimated concentration in CSF and biological function. Peptide surrogates for internal standards were selected according to relevant criteria, parallel reaction monitoring (PRM) assays created, and extensive assay quality testing performed, i.e. intra- and inter-day variation, trypsin digestion status over time, and whether the peptides were able to separate multiple sclerosis patients and controls.

**Results:**

Assays were developed for 25 proteins, represented by 72 peptides selected according to relevant guidelines and available literature and tested for assay peptide suitability. Stability testing revealed 64 peptides with low intra- and inter-day variations, with 44 also being stably digested after 16 h of trypsin digestion, and 37 furthermore showing a significant difference between multiple sclerosis and controls, thereby confirming literature findings. Calibration curves and the linear area of measurement have, so far, been determined for 17 of these peptides.

**Conclusions:**

We present 37 high-quality PRM assays across 21 CSF-proteins found to be affected by multiple sclerosis, along with a recommended workflow for future development of new assays. The assays can directly be used by others, thus enabling better comparison between studies. Finally, the assays can robustly and stably monitor biological processes in multiple sclerosis patients over time, thus potentially aiding in diagnosis and prognosis, and ultimately in treatment decisions.

## Background

There are currently only a few biomarkers for multiple sclerosis (MS) in clinical use, including MRI (T2-weighted lesions), oligoclonal bands and IgG ratio, JC viral antibody titers and neurofilament light, as summarized in a recent review [1]. However, both in-house discovery studies and available literature suggest numerous additional biomarkers representing several of the processes and pathways active in MS, such as inflammation and neurodegeneration [2–5]. Such findings however require further verification via robust targeted assays, e.g. through parallel reaction monitoring (PRM) using high-quality stable isotope labelled heavy peptides [6–8].

The process of developing robust targeted assays in turn requires the consideration of a multitude of factors in order to ensure the quality and relevance of the assays, especially when the goal is to provide absolute protein measurements that would allow the consecutive analyses of proteins both across different labs and over time. This will make it possible to monitor specific pathological processes occurring in individual MS patients and thereby gain a deeper insight into the processes active at the various stages of the disease, which in turn would be a valuable tool in diagnosis, prognosis and treatment decisions.

Cerebrospinal fluid (CSF) is a commonly used body fluid in studies of neurological diseases, such as MS. Although not as easily accessible as serum/plasma, it is likely to better reflect ongoing neurological processes as it is in direct contact with the central nervous system [9]. However, large scale biomarker verification of discovery experiments has proven difficult in CSF and results are rarely consistent between studies. Likely reasons for this are methodological differences, large individual variations in total CSF protein concentrations [10] as well as significant heterogeneity in neurological diseases [11–13]. As a consequence, the quantitative data from individual biomarker discovery and verification studies do not always overlap and cannot directly and easily be compared and combined [14]. The large dynamic range of proteins in CSF also leads to challenges when measuring small disease-related changes in low abundant proteins [13, 15], especially vulnerable to small methodological variations and inaccuracies. Combined with relatively low patient numbers in most studies, it becomes almost impossible to conclude regarding a biomarker’s potential, and thus move from the biomarker discovery phase to clinically useful biomarkers. It is therefore crucial to create robust targeted assays for accurate measurements of biomarker candidates.

Here we describe a suggested standard approach for the selection of candidate biomarkers in CSF for MS, and detail the required validation of PRM assays for absolute quantification of 25 proposed biomarker candidates. The validation includes (i) intra- and inter-day variation, (ii) the effect of trypsin digestion time, and (iii) verification of the separation capability between MS and controls observed from the literature [14]. Additionally, the linearity around the typical concentration of target peptides was determined and corresponding response curves determined. The validated assays are ready to be used in large-scale analysis of patient samples and the presented standard approach for PRM assay development can also be applied for other neurological diseases.

## Results—from biomarker candidates to high-quality PRM assays

**Fig. 1:**
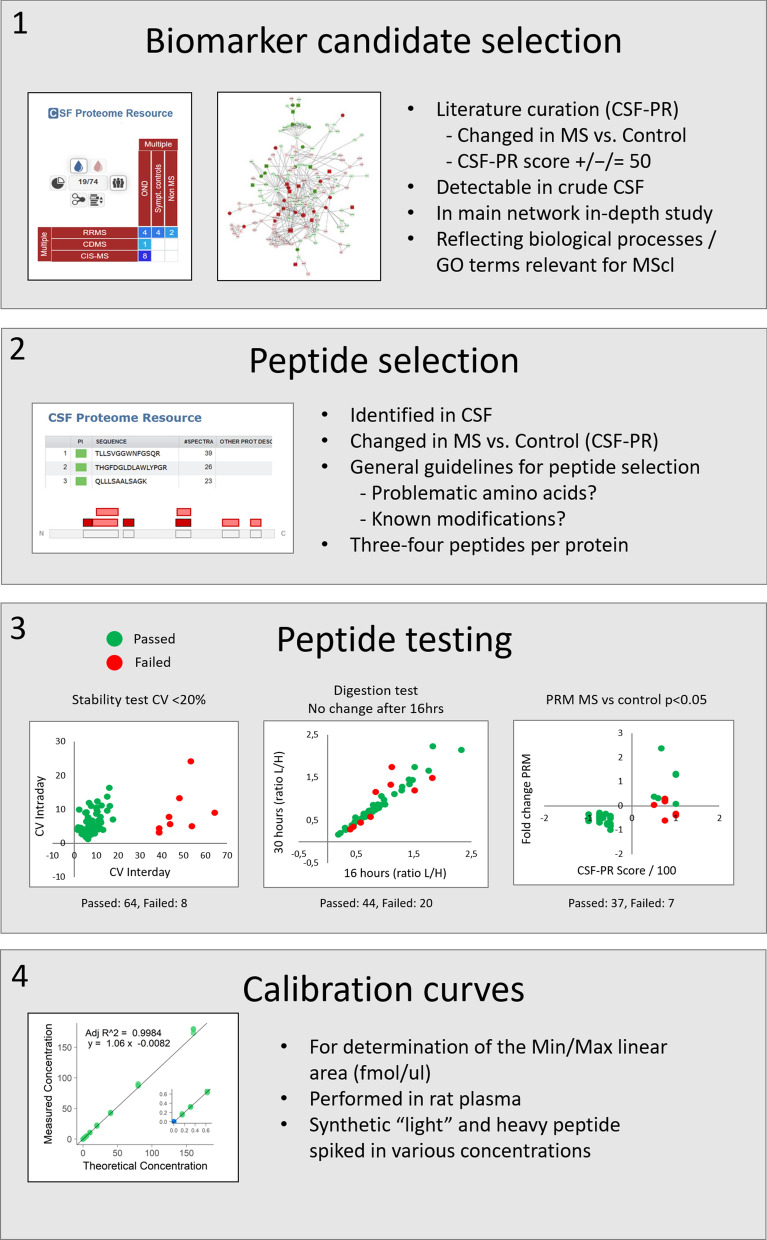
The main steps in developing robust PRM assays for CSF biomarkers related to MS

The following sections describes the steps from potential literature-based biomarker candidates from CSF-PR (proteomics.uib.no/csf-pr), to the list of the most promising proteins and peptides to include in robust high-quality PRM biomarker assays for MS (Fig. 1).

## Selection of proteins and peptides

### Literature curation using CSF-PR

The recently published CSF-PR 2.0 [14] contains structured and searchable quantitative data for thousands of CSF-proteins from close to 100 datasets related to MS, Alzheimer’s disease (AD) and Parkinson’s disease (PD). The data in CSF-PR comes from mass spectrometry studies that have passed certain filters notably related to (i) methodology (bottom-up shotgun or targeted proteomics for main experiment; ELISA for verification), (ii) number of patients (n ≥ 5 in each disease group; if using pools, ≥ 3 pools for each disease group and n ≥ 20 total), and (iii) sample type (CSF from living subjects).

The biomarker selection was conducted by merging relevant datasets from MS and control subcategories in CSF-PR and extracting the proteins found to be significantly different in abundance in the majority of studies, according to certain criteria. See “Materials and methods” section for more details. The CSF-PR data extraction resulted in an initial list of 133 proteins (Additional file 1: Table S1), representing promising biomarker candidates for MS quantified in several experiments where various degree of fractionation had been used. Separately, we also collected a list of proteins that were changed between MS and control, but quantified in only one study in CSF-PR (Additional file 2: Table S2). Most of the latter were proteins from our recent discovery study [5], where both depletion and extensive peptide fractionation was performed, and they are therefore likely the lowest abundant proteins possible to quantify by current mass spectrometry proteomics technology.

### Identifying proteins within the suitable dynamic range of CSF

An important condition for assay development is to be able to perform PRM quantitation in crude CSF without high-abundant protein depletion or peptide fractionation. In order to find the proteins most likely fulfilling this condition, a CSF fractionation test was carried out, where the trypsin digested CSF-proteome was separated into eleven fractions and analysed by data-dependent acquisition (DDA) MS/MS, resulting in 1194 proteins. We estimate that PRM can be ten times more sensitive than what can be identified in a regular DDA shotgun experiment [16]. Therefore, the identification of a protein in a 20 µg un-depleted sample, fractionated into 11 fractions (first fraction usually does not contain peptides), indicates that the protein can be quantified by PRM in crude CSF. A total of 120 of the 132 proteins extracted from CSF-PR were identified in the DDA analysis (Additional file 3: Table S3) and passed on to the next steps in the assay development. All of the proteins identified in the DDA analysis can be found in Additional file 4: Table S4.

### Biological processes and categories

A closer inspection of the 120 proteins revealed several groups of related proteins with similar names, functions and abundance relationship between MS and control, e.g. immunoglobulin proteins, cadherin proteins, receptor-type tyrosine-protein phosphatases, and SLIT and NTRK-like proteins. These proteins are likely to be involved in the same biological processes, and therefore developing individual PRM assays for all these proteins is probably not necessary, as recent studies indicate that such related proteins are most-often affected in the same manner [4, 5].

**Fig. 2:**
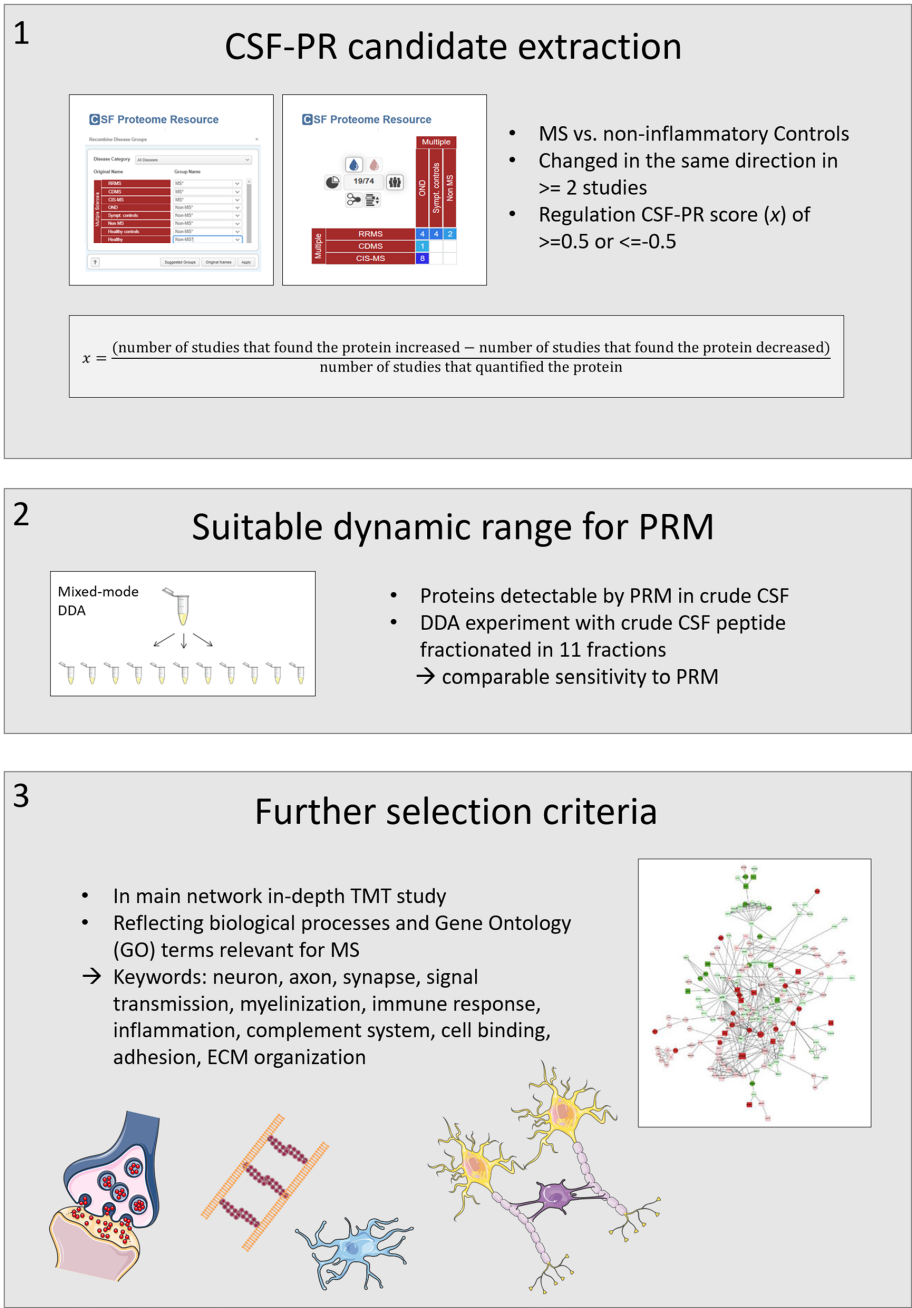
The main steps in the identification and selection of biomarker candidate proteins for inclusion in the PRM assays. Screenshots are from CSF-PR [14], PPI network is from [5] and other figures are from Servier Medical Art licensed under a Creative Commons Attribution 3.0 Unported License. TMT = tandem mass tag, DDA = data dependent acquisition, MM-RP AX = mixed-mode reversed-phase anion exchange [18], GO = gene ontology, ECM = Extracellular matrix

A representative selection of the 120 proteins from Additional file 3: Table S3 was made based on available information from CSF-PR, i.e. the number and proportion of studies where changes between MS and Non-MS was observed. Additionally, the large network of interacting and significantly changed proteins between MS and other neurological diseases (OND) generated in our recent publication [5] was utilized to select one or two proteins as representatives of the various biological processes likely to be affected in MS. These processes include e.g. (i) inflammation—a hallmark of the MS disease, (ii) extracellular matrix organization proteins—providing structure and support for developing neurons (e.g. collagens and proteoglycans), (iii) ephrin proteins—involved in neuron development, myelination and axonal guidance, and (iv) cadherins—cell adhesion proteins known to be involved in de- and re-myelination. Additional proteins found especially interesting based on keywords in UniProt [17] or our own previous experiments were also included. All the steps in the protein selection process is outlined in Fig. 2, and the 25 selected proteins are shown in Table 1.

**Table 1:** The 25 proteins selected for assay development

Accession	Name	Network*	CSF-PR score**	Datasets***	Selected keywords from UniProt and Gene Ontology
P51693	Amyloid-like protein 1	Yes	− 50	1 ↑ [56] 1 – [57] 4 ↓ [4, 5, 56]	Postsynaptic function, neurite outgrowth, neuronal apoptosis
P61769	Beta-2-microglobulin	Yes	60	3 ↑ [4, 5] 2 – [25, 57]	Component of the class I MHC, antigen presentation, innate immune response
P55290	Cadherin-13	Yes	− 75	3 ↓ [4, 5] 1 – [25]	Cell adhesion, negative regulator of neural cell growth
P16070	CD44 antigen	Yes	75	3 ↑ [4, 5] 1 – [25]	Mediates cell–cell and cell–matrix interactions, cell migration
P36222	Chitinase-3-like protein 1		70	8 ↑ [4, 5, 24, 25, 56] 1 – [22] 1 ↓ [56]	Lectin that binds glycans, no chitinase activity, inflammatory response, macrophage differentiation
Q15782	Chitinase-3-like protein 2		100	6 ↑ [4, 5, 25]	Lectin that binds glycans, no chitinase activity, carbohydrate metabolic process
P10645	Chromogranin-A		− 50	4 ↓ [4, 5, 22] 4 – [2, 25, 57]	Innate immune response, defence response (fungus, bacterium), negative regulation of neuron death
P12111	Collagen alpha-3(VI) chain	Yes	50	2 ↑ [4, 5] 2 – [4, 25]	Cell binding/adhesion, extracellular matrix organization
P02747	Complement C1q subcomponent subunit C	Yes	100	3 ↑ [4, 5]	Complement system, immune response
P00736	Complement C1r subcomponent	Yes	75	3 ↑ [4, 5] 1 – [25]	Complement system, immune response
P54764	Ephrin type-A receptor 4	Yes	− 100	5 ↓ [4, 5, 56]	RTK signalling, promiscuous, prevents axonal regeneration, cell adhesion, cell signalling, repair after injury in the nervous system, axonal guiding
Q6MZW2	Follistatin-related protein 4		− 75	3 ↓ [4, 5] 1 – [25]	Negative regulation of dendritic spine development and collateral sprouting
P48058	Glutamate receptor 4	Yes	− 50	2 ↓ [4, 5] 2 – [4, 25]	Excitatory synaptic transmission
P01591	Immunoglobulin J chain		67	2 ↑ [4], 1 – [5]	Links monomers of IgM or IgA, antigen binding, immune response
Q92876	Kallikrein-6	Yes	− 60	1 ↑ [56] 2 − [–2, 25] 7 ↓ [2, 4, 5, 22, 56, 57]	Serine protease, Indicated in AD, regulation of axon outgrowth after injury, myelination
P32004	Neural cell adhesion molecule L1	Yes	− 75	3 ↓ [4, 5] 1 – [25]	Nervous system development, neuron–neuron adhesion, neuronal migration, axonal growth, synaptogenesis
Q9ULB1	Neurexin-1		67	2 ↓ [4, 5] 1 – [4]	Cell surface protein, cell–cell interactions, axon guidance, signal transmission, neurotransmitter release
Q9P2S2	Neurexin-2		− 75	3 ↓ [4, 5] 1 – [25]	Neuronal cell surface protein, cell recognition, adhesion, signalling
Q92823	Neuronal cell adhesion molecule		− 75	3 ↓ [4, 5] 1 – [25]	Neurite outgrowth. cell–cell contacts between Schwann cells and axons. formation and maintenance of the nodes of Ranvier on myelinated axons.
Q99983	Osteomodulin	Yes	75	3 ↑ [4, 5] 1 – [25]	Biomineralization processes, cell adhesion, extracellular matrix
Q9UHG2	ProSAAS	Yes	− 75	3 ↓ [4, 5] 1 – [25]	Control of the neuroendocrine secretory pathway.
P23468	Receptor-type tyrosine-protein phosphatase delta	Yes	− 100	2 ↓ [4, 5]	Phosphatase, pre- and post-synaptic differentiation of neurons
O00584	Ribonuclease T2		100	3 ↑ [4, 5]	Lysosomal degradation of ribosomal RNA
P13521	Secretogranin-2		− 57	4 ↓ [4, 5, 22] 3 – [2, 25]	Neuroendocrine secretory granule protein
Q6UXD5	Seizure 6-like protein 2		− 50	2 ↓ [4, 5] 2 – [4, 25]	Specialized ER function in neurons?

Relevant details for the selected proteins, such as whether or not they were found in the main protein–protein interaction network in our recent in-depth discovery study [5], their CSF-PR score, studies that found them increased or decreased in MS vs. Non-MS and selected gene ontology terms and keywords related to their function. Arrows pointing down: decreased abundance in MS; arrows pointing up: increased abundance in MS; Dash: no change in abundance between MS and Non-MS

* Proteins found in the main protein interaction network from [4]

** The score for MS vs. Non-MS calculated by CSF-PR according to the equation described in “Materials and methods” section

*** Multiple datasets can be from the same paper

### Peptide selection

Selecting peptides to represent the proteins under investigation, so-called surrogate or signature peptides, is a crucial step in the development of a targeted proteomics assay, and a number of criteria determines if a peptide is suitable [19, 20]. Here, the initial peptide selection was done mainly based on peptide data available from CSF-PR combined with general guidelines for selecting peptides for targeted proteomics [6, 19–21]. As a rule, three- to four peptides were selected per protein and the corresponding isotopically heavy labelled versions were ordered. However, not all peptides were found with an acceptable signal in the MS/MS analysis, hence, some of the proteins are only represented by a single peptide. For one protein (chitinase-3-like protein 1), more than three heavy peptides were ordered given that this protein has been reported as particularly interesting in relation to MS [22–28], and we had previously experienced that this protein could be challenging to quantify (data not shown). In total, 72 peptides were selected to represent the 25 proteins (Table 2). Further testing was performed to determine whether they were truly suitable as protein surrogates, as outlined below.

**Table 2:** The 72 signature peptides selected for the 25 proteins

Accession	Protein name	# Peptides	Peptide sequence(s)
P51693	Amyloid-like protein 1	3	WEPDPQR
			FQVHTHLQVIEER
			GFPFHSSEIQR
P61769	Beta-2-microglobulin	2	VEHSDLSFSK
			VNHVTLSQPK
P55290	Cadherin-13	3	YEVSSPYFK
			VNSDGGLVALR
			INENTGSVSVTR
P16070	CD44 antigen	3	FAGVFHVEK
			ALSIGFETCR
			YGFIEGHVVIPR
P36222	Chitinase-3-like protein 1	7	EGDGSCFPDALDR
			TLLSVGGWNFGSQR
			GTTGHHSPLFR
			EAGTLAYYEICDFLR
			ILGQQVPYATK
			GNQWVGYDDQESVK
			FPLTNAIK
Q15782	Chitinase-3-like protein 2	2	LVCYFTNWSQDR
			LLLTAGVSAGR
P10645	Chromogranin-A	3	ILSILR
			SGELEQEEER
			EDSLEAGLPLQVR
P12111	Collagen alpha-3(VI) chain	2	EVYTFASEPNDVFFK
			WYYDPNTK
P02747	Complement C1q subcomponent subunit C	3	QTHQPPAPNSLIR
			FNAVLTNPQGDYDTSTGK
			TNQVNSGGVLLR
P00736	Complement C1r subcomponent	4	TLDEFTIIQNLQPQYQFR
			NLPNGDFR
			ESEQGVYTCTAQGIWK
			LPVANPQACENWLR
P54764	Ephrin type-A receptor 4	3	VYPANEVTLLDSR
			NLAQFPDTITGADTSSLVEVR
			GLNPLTSYVFHVR
Q6MZW2	Follistatin-related protein 4	3	GPDVGVGESQAEEPR
			FDDYNSDSSLTLR
			VLQSIGVDPLPAK
P48058	Glutamate receptor 4	3	NTDQEYTAFR
			LQNILEQIVSVGK
			EYPGSETPPK
P01591	Immunoglobulin J chain	2	SSEDPNEDIVER
			IIVPLNNR
Q92876	Kallikrein-6	3	LSELIQPLPLER
			TADGDFPDTIQCAYIHLVSR
			DSCQGDSGGPLVCGDHLR
P32004	Neural cell adhesion molecule L1	3	INGIPVEELAK
			AQLLVVGSPGPVPR
			EGPGEAIVR
Q9ULB1	Neurexin-1	3	DLFIDGQSK
			SDLYIGGVAK
			LPDLISDALFCNGQIER
Q9P2S2	Neurexin-2	3	LSALTLSTVK
			GATADPLCAPAR
			AIVADPVTFK
Q92823	Neuronal cell adhesion molecule	3	AETYEGVYQCTAR
			SLPSEASEQYLTK
			VFNTPEGVPSAPSSLK
Q99983	Osteomodulin	2	IDYGVFAK
			LLLGYNEISK
Q9UHG2	ProSAAS	1	ALAHLLEAER
P23468	Receptor-type tyrosine-protein phosphatase delta	3	SPQGLGASTAEISAR
			ILYDDGK
			SYSFVLTNR
O00584	Ribonuclease T2	2	ELDLNSVLLK
			VYGVIPK
P13521	Secretogranin-2	3	DQLSDDVSK
			TSYFPNPYNQEK
			VLEYLNQEK
Q6UXD5	Seizure 6-like protein 2	3	VSLDEDNDR
			FEAFEEDR
			TASDAGFPVGSHVQYR

### Peptide stability testing

**Fig. 3:**
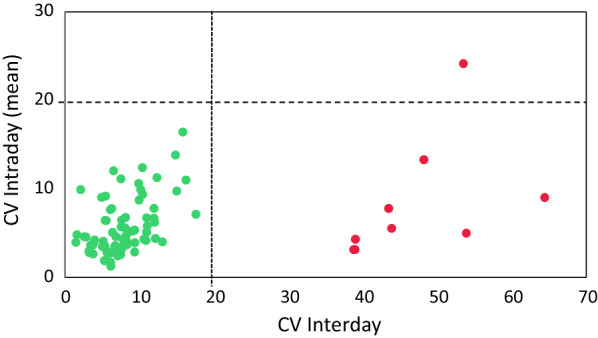
Inter- (*x*-axis) and intraday (mean, *y*-axis) CV for each peptide illustrated as green (CV less than 20%) and red (CV greater than 20%) dots. Failing peptides (red) are from the protein chitinase-3-like protein 1 (failed by inter-day, below horizontal line) and Seizure-6-like protein 1

PRM assays ought to have low intra- and inter-day variation in order to allow comparable quantitative measurements over time. To test this, PRM experiments with two replicates were processed each day across 5 days, and the intra- and inter-day coefficient of variation (CV) was calculated for all the 72 peptides. Most of the peptides displayed an intra- and inter-day CV of less than 20% (Fig. 3). Only eight of the initial 72 peptides had a CV above 20%, with seven from chitinase-3-like protein 1 (CH3L1), failing only the inter-day CV, and one from Seizure 6-like protein 2 (SEZ6L2), failing both intra- and inter-day CV. Notably, none of the peptides from CH3L1 showed an acceptable inter-day CV. Peptides not fulfilling the intra- and inter-day CV limits were discarded, resulting in 64 peptides from 24 proteins retained. Detailed results from this experiment can be found in Additional file 5: Table S5.

### Peptide digestion testing

In order to create assays for absolute protein concentrations in CSF samples, it is important to investigate how the trypsin incubation time affects the quantitative results. The main question is whether the detected amount of a peptide continues to increase after completing a standard trypsin digestion protocol with 16 h digestion time (see “Materials and methods” section), as then the absolute concentration of the corresponding protein cannot be determined via such a standard digestion protocol using the given peptide.

The experiment investigated five different digestion times (1, 5, 16, 24 and 30 h), each with three replicates, and was repeated three times. A peptide was considered stably digested after 16 h if the percentage change from 16 to 24 h and from 16 to 30 h was both less than 20%. In addition, the resulting peaks had to be satisfactory with regards to intensity, interference and shape, evaluated through the Skyline [29] data analysis. A total of 44 peptides, with at least one peptide from each of the 24 proteins, passed the digestion test. In other words, 20 peptides, but no proteins, were discarded based on this test.

**Fig. 4:**
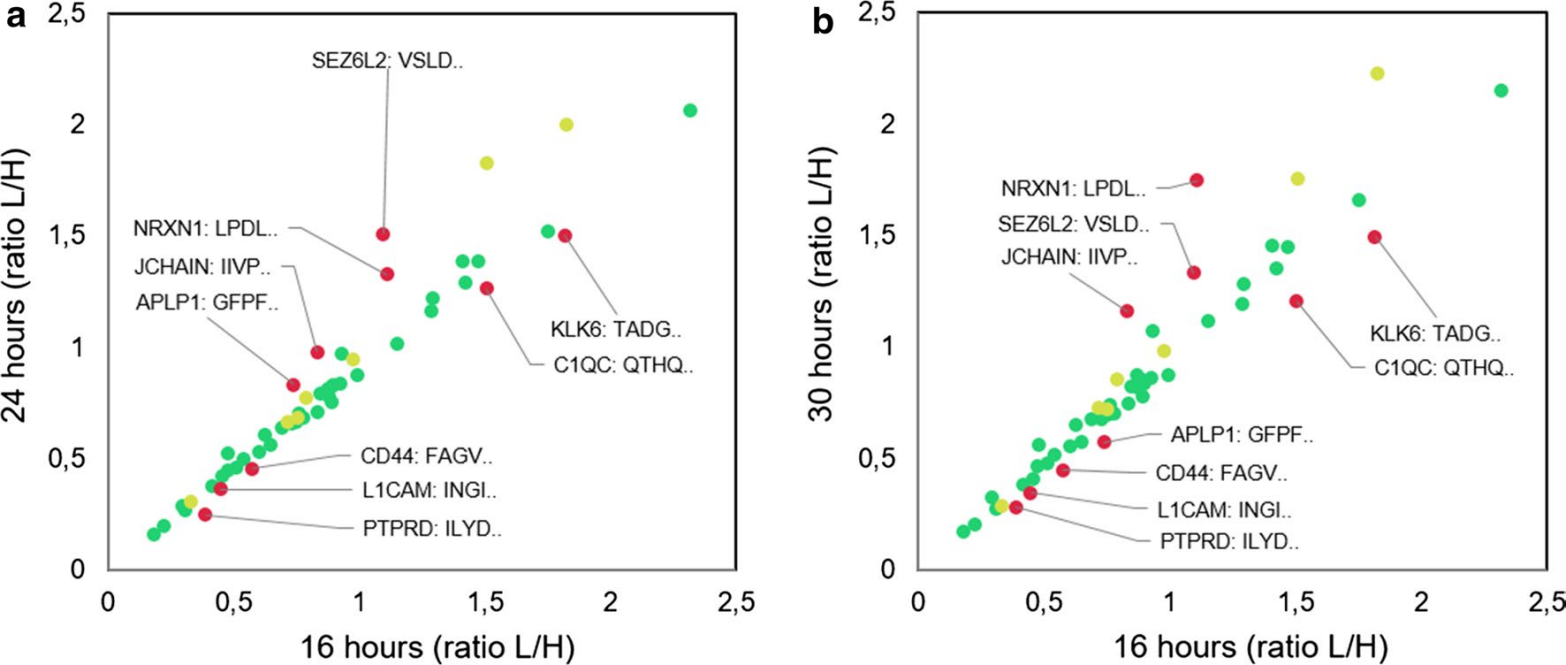
Peptide amounts at different trypsin digestion times. Peptide amounts (mean ratio light/heavy) of the tested peptides at 16 h compared to 24 h (**a**) and 30 h (**b**). Green dots represent peptides with < 20% change after 16 h of digestion and CV < 20% between replicates, yellow dots represent peptides with < 20% change after 16 h digestion, but with CV > 20% between replicates and red dots represent peptides with > 20% change after 16 h digestion. Protein short name and the four first amino acids in the peptide sequence is shown for all peptides failing this test. The two peptides AQLLVVGSPGPVPR and ELDLNSVLLK are not included in this plot due to very high values compared to the rest. Measured abundance change at all time points are available in Additional file 8: Fig. S2

**Fig. 5:**
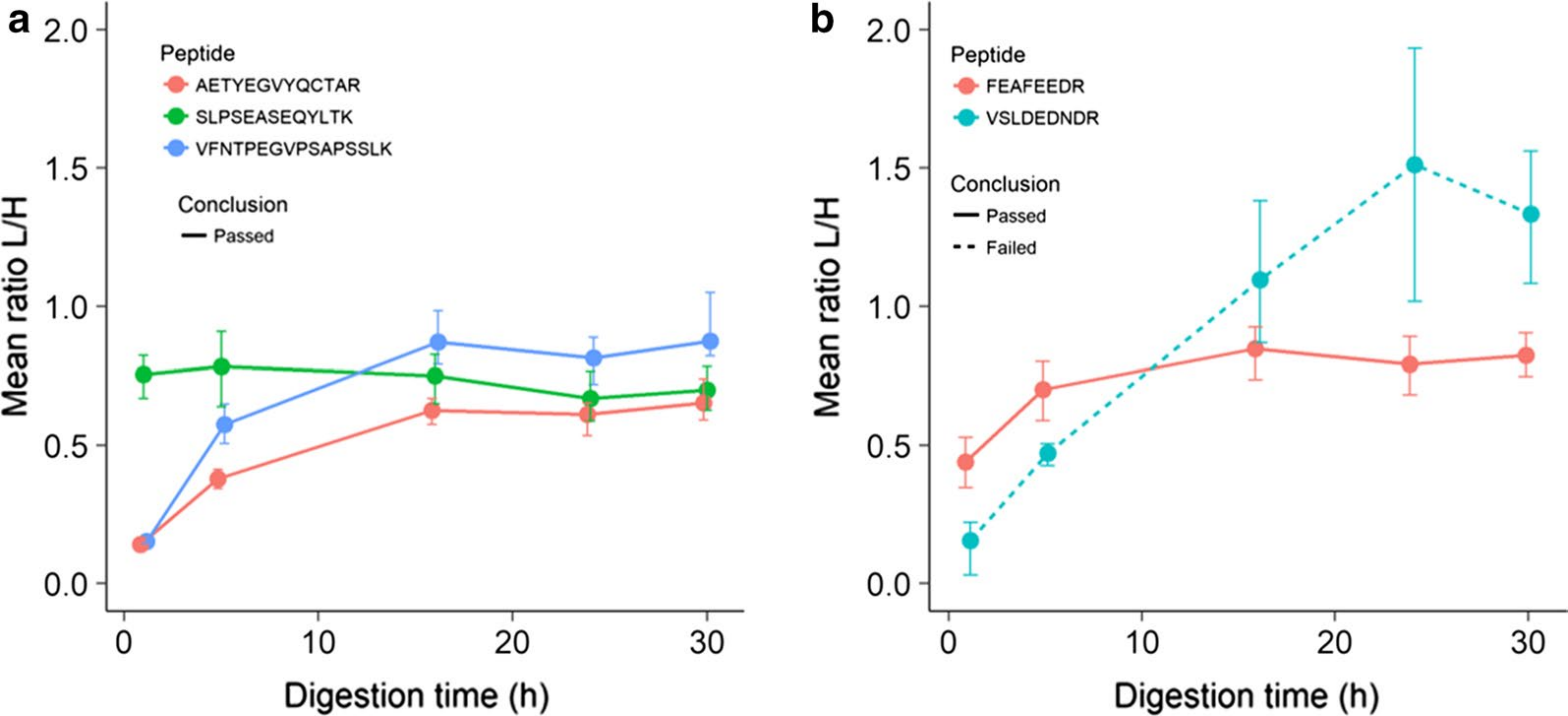
Peptide digestion profile examples. Peptide amount (mean ratio light/heavy) at all tested digestion times for the peptides representing Neuronal cell adhesion protein (**a** all peptides passed) and Seizure-6-like protein 2 (**b** one peptide passed, one failed). Error bars represent min and max values measured. Similar digestion profiles of all tested peptides are available in Additional file 7: Fig. S1. The figure was created using R (http://www.R-project.org.) and ggplot2 (https://ggplot2.tidyverse.org)

How the peptides changed (ratio light/heavy) after 16 h is illustrated in Fig. 4a, b, where red dots represent the peptides who failed the test. Examples of observed peptide profiles for two selected proteins are shown in Fig. 5a, b.

As can be seen from Fig. 5a, all of the three peptides from neuronal cell adhesion molecule show limited increase after 16 h of digestion, i.e. they all passed the test. However, some proteins demonstrated an increase in peptide amount after 16 h and/or a varying digestion profile for the different peptides. As an example, we see that peptide SLPSEASEQYLTK in Fig. 5a appears to be readily digested already after 1 h. Other proteins had some peptides passing and some failing the digestion test. In Fig. 5b, we see that one peptide for the protein Seizure-6-like protein 2 increase in amount up to 16 h of digestion, and then no increase beyond 20% is found (peptide passed), while a different peptide from the same protein continue to increase after 16 h, thereby failing the test, notably with a high variation in the minimum and maximum values.

All the data from this experiment is available in Additional file 6: Table S6 and the complete digestion profile for all peptides can be found in Additional file 7: Fig. S1 and peptide abundance change at all measured time points are in Additional file 8: Fig. S2.

### RRMS vs non-inflammatory controls

To test whether the changes indicated in CSF-PR between MS and Non-MS could be reproduced, a small PRM study was conducted using six pools of CSF from MS (three pools of seven RRMS patients) and control (three pools of seven OND patients) patients. These pools have previously been analysed in-depth by shotgun TMT-based proteomics [5], and were selected to test how well the optimized PRM assays reflect the differences between the two patient categories.

**Fig. 6:**
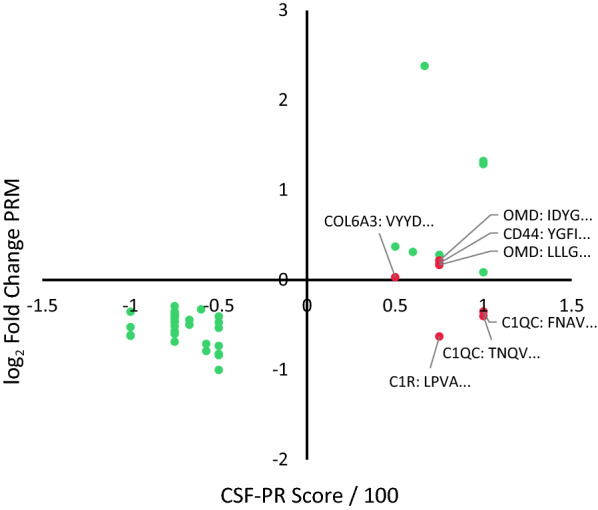
CSF-PR score (MS vs. Non-MS) compared to fold change from PRM comparison study (RRMS vs. OND). Comparison of the score found from CSF-PR (x-axis, score/100), representing the direction of change between MS and Non-MS in the literature, and the fold change (y-axis, log_2_ transformed) found in our PRM study. Green dots represent peptides that were found significantly changed in the same direction in the literature and in the PRM study (passed) and red dots represent peptides that either were not significant or were significantly changed, but in opposite direction compared to the literature (failed). Protein short name and the four first amino acids in the peptide sequence is shown for all peptides failing this test

The majority of the 44 tested peptides showed similar regulation trends as previously reported in the literature (Fig. 6), but a couple of the peptides were not found significantly changed in this study (Osteomodulin—two peptides, Complement C1q subcomponent subunit C—one peptide, Collagen alpha-3(VI) chain—one peptide, CD44 antigen—one peptide), and two peptides showed the opposite direction of regulation compared to CSF-PR (Complement C1q subcomponent subunit C—one peptide, Complement C1r subcomponent—one peptide). These were therefore discarded from further assay development. In conclusion, seven of 44 tested peptides failed this test.

We concluded that the remaining 37 peptides were suitable to reflect the previously reported regulations, and therefore represent the most promising biomarkers for MS. The complete results from this test is found in Additional file 9: Table S7.

### Final assay peptides

After all the steps detailed above, we finally arrived at a list of 37 peptides from 21 proteins for which promising absolute quantitative PRM assays could be developed (Table 3). These represent the best surrogates to precisely and reproducibly quantitate proteins affected by MS. A complete table of all the tested peptides, important results and data from each experiment is collected in Additional file 10: Table S8, and a protein level overview of the number of peptides passing the various tests can be found in Additional file 11: Table S9.

**Table 3:** The most promising biomarker candidate proteins and peptides

Accession	Protein name	Peptide sequence(s)	Highest in	Cal.curve	MinLin (fmol/µl)	MaxLin (fmol/µl)
P51693	Amyloid-like protein 1	WEPDPQR	Control	Yes	0.525	560
		FQVHTHLQVIEER	Control	No		
P61769	Beta-2-microglobulin	VNHVTLSQPK	MS	No		
P55290	Cadherin-13	YEVSSPYFK	Control	Yes	0.15	160
		INENTGSVSVTR	Control	No		
P16070	CD44 antigen	ALSIGFETCR	MS	Yes	0.105	112
Q15782	Chitinase-3-like protein 2	LVCYFTNWSQDR	MS	Yes	0.045	48
		LLLTAGVSAGR	MS	Yes	0.045	48
P10645	Chromogranin-A	ILSILR	Control	No		
		SGELEQEEER	Control	Yes	0.75	800
		EDSLEAGLPLQVR	Control	No		
P12111	Collagen alpha-3(VI) chain	EVYTFASEPNDVFFK	MS	No		
P54764	Ephrin type-A receptor 4	VYPANEVTLLDSR	Control	Yes	0.075	80
		NLAQFPDTITGADTSSLVEVR	Control	No		
Q6MZW2	Follistatin-related protein 4	GPDVGVGESQAEEPR	Control	No		
		FDDYNSDSSLTLR	Control	No		
		VLQSIGVDPLPAK	Control	Yes	0.045	48
P48058	Glutamate receptor 4	NTDQEYTAFR	Control	Yes	0.09	96
P01591	Immunoglobulin J chain	SSEDPNEDIVER	MS	No		
Q92876	Kallikrein-6	DSCQGDSGGPLVCGDHLR	Control	Yes	0.15	160
P32004	Neural cell adhesion molecule L1	AQLLVVGSPGPVPR	Control	Yes	0.045	48
		EGPGEAIVR	Control	No		
Q9ULB1	Neurexin-1	DLFIDGQSK	Control	No		
		SDLYIGGVAK	Control	Yes	0.045	48
Q9P2S2	Neurexin-2	LSALTLSTVK	Control	Yes	0.045	48
		GATADPLCAPAR	Control	No		
		AIVADPVTFK	Control	No		
Q92823	Neuronal cell adhesion molecule	AETYEGVYQCTAR	Control	No		
		SLPSEASEQYLTK	Control	Yes	0.15	160
		VFNTPEGVPSAPSSLK	Control	No		
Q9UHG2	ProSAAS	ALAHLLEAER	Control	No		
P23468	Receptor-type tyrosine-protein phosphatase delta	SPQGLGASTAEISAR	Control	No		
		SYSFVLTNR	Control	Yes	0.045	48
O00584	Ribonuclease T2	VYGVIPK	MS	No		
P13521	Secretogranin-2	DQLSDDVSK	Control	Yes	0.045	48
		VLEYLNQEK	Control	Yes	0.18	192
Q6UXD5	Seizure 6-like protein 2	FEAFEEDR	Control	No		

Proteins and peptides passing all quality controls described in this study, thereby representing the most promising biomarker candidates for PRM assays. The table also shows if the protein is highest in MS or control, if calibration curves have been developed, and, if so, its linear area

Cal.curves: Calibration curves. MinLin: Lowest theoretical concentration that will be used for quantitation. MaxLin: Maximum theoretical concentration that will be used for quantitation

### Calibration curves

**Fig. 7:**
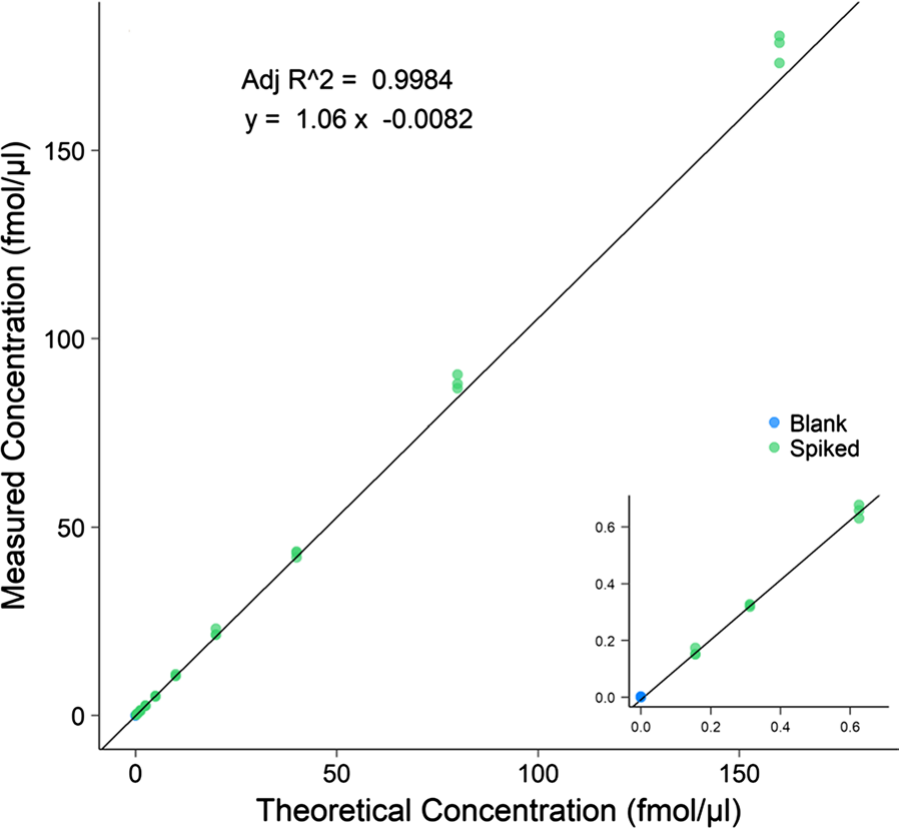
Calibration curve for the peptide YEVSSPYFK from the protein Cadherin-13 in rat plasma. Blank values (blue) indicates abundance of background without spike-in of endogenous peptide but spike-in of heavy. Spiked samples (green) have both endogenous and heavy spike in. The linearity of the lowest concentrations is shown in the smaller graph for increased visibility. The experiments were conducted in trypsinated triplicates. The figure was created using R (http://www.R-project.org) and ggplot2 (https://ggplot2.tidyverse.org)

To ensure that the peptides could be accurately quantified by PRM mass spectrometry around the level of its observed concentration in CSF, calibration curves determining the linear areas of quantification have to be determined. Calibration curves have so far been generated for 17 of the peptides passing the initial testing and the work is ongoing. Rat plasma was used as matrix with varying amounts of synthetic light peptide and stable spike-in of heavy surrogate peptide. The linear area was determined by weighed least squares regression. For further details see “Materials and methods” section. An example of a calibration curve is shown in Fig. 7 and all of the calibration curves developed so far are provided in Additional file 12: Fig. S3 and additional details related to slope, intercept and linear areas are in Additional file 10: Table S8.

## Discussion

The verification of biomarker candidates in CSF from discovery studies has been challenging due to the many issues pointed out in the introduction. One of the major bottlenecks has been to simultaneously measure a larger number of proteins in a high number of samples in a reproducible fashion and over time. In our view there is a need to develop well-described high-quality assays able to generate reproducible data over time, and ideally also between laboratories, in order to achieve efficient biomarker verification in CSF.

Recently, there has been at least two publications going in the direction of generating high-quality PRM assays for CSF-proteins; one describing assays for 30 brain proteins [30], and another assays for monitoring a set of defined biological process [31]. In our study, we have contributed towards this idea by developing 37 well-described high-quality PRM peptide assays representing 21 proteins found to be affected in multiple sclerosis across multiple studies. Our goal is that these assays can be used to generate comparable data over time and provide the possibility to analyse and compare the protein levels in a large number of patient samples in a long-term perspective.

An important aspect of this work is that the biomarker candidates have been selected based on data from several studies using the online database CSF-PR. This approach is likely to provide less false positive candidates as more data, most often from several research groups, is used in the selection. Furthermore, the surrogate peptides have been selected based on quantitative information available from CSF-PR (when available), indicating that the particular peptide is actually regulated in the target protein. In sum, we argue that our approach is a step forward in increasing the effectiveness of verifying biomarker candidates in CSF.

### Selection of proteins and peptides—what is important to consider?

Using CSF-PR as a starting point for selecting proteins affected by MS differs substantially from using a single experiment as the basis for selection, and the 133 proteins initially identified thus represents the proteins collectively reported to be affected by MS from the mass spectrometry proteomics literature. In our view, this approach increase the chance of including the most relevant proteins and those more likely affected by MS, compared to basing the selection on a single study.

Next, we wanted to make sure that all of the proteins included in the assay development was possible to quantify in a PRM experiment without the need for protein depletion or peptide fractionation, as these steps both have their drawbacks. Targeted immunoaffinity depletion of high-abundant proteins is a useful way to increase the chance of measuring low-abundant proteins. Depletion is however a debated approach in biomarker studies, given that proteins not targeted by the depletion column may be co-depleted due to unspecific binding and protein interactions, potentially introducing a bias already at an early stage in the sample preparation [32–34]. As for peptide fractionation, this step will increase the analysis time, cost and complexity, and is therefore not desirable.

To arrive at a more manageable number, and as a demonstration of our suggested workflow, we selected 25 proteins. These proteins will of course not reflect all off the disease-affected processes represented by the complete list of 120 proteins, nor are they meant to represent a final list of biomarker candidates for multiple sclerosis. However, they do cover a range of functions and processes relevant in the MS disease as summarized in Table 1.

### Creating PRM assays for all peptides from all potentially interesting CSF-proteins would be preferable, but as there will always be a cost vs. benefit consideration this is not realistic

**Fig. 8:**
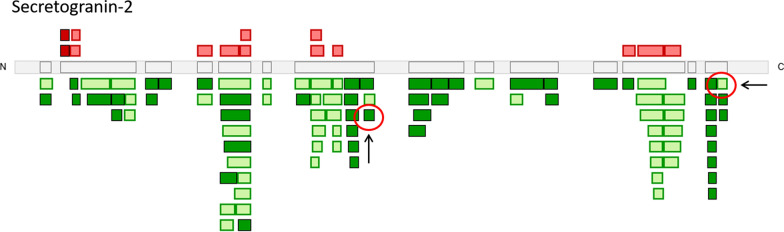
Illustration of the peptide quantitative data from one dataset in CSF-PR for the protein Secretogranin-2. Quantified peptide sequences are displayed as green or red boxes, covering various segments of the protein sequence (grey) from N- to C-terminal. Dark red and dark green indicate peptides that are significantly different between the compared disease groups, and light red and light green indicate non-significant peptides. Arrows and red circles indicate selected peptides for this particular protein in the assay development

Using the peptide level quantitative information available in CSF-PR as part of the surrogate peptide selection was also considered important. For the disease-affected proteins we observed that not all of the peptides were regulated in the discovery data, and that some peptides were also regulated in the opposite direction (Fig. 8). We suggest inspecting and using the peptide level data available in CSF-PR to select peptides that are observed as significantly changed, thus increasing the chance of the peptide actually representing the regulation reported at the protein level.

Different peptides from the same protein may show different (relative) abundance due to: (i) peptides mapping to multiple proteins or proteoforms with different regulation status, (ii) some peptides can be less suitable for mass spectrometry or in too low amounts for stable and accurate quantitation, and (iii) certain peptides have varying degrees of post-translational modifications, resulting in unstable concentration for the unmodified form. In addition, it is important to consider the general guidelines for peptide selection in targeted proteomics, i.e. to avoid non-unique peptides and peptides prone to mis-cleavages.

### Peptide stability testing—most peptides are stable across runs

A large majority of the tested peptides passed our stability test, indicating that they are suitable for PRM monitoring robustly over time. We can conclude that they are in the appropriate concentration range in CSF for the method to consistently provide a sufficient signal for stable measurements.

#### Chitinase-3-like protein 1 peptides give unstable measurements over time

Chitinase-3-like protein 1 (CH3L1) has been linked to several neurological diseases [23, 26, 35–39], including MS [22, 24, 25, 27, 28]. However, it seems that this protein is not ideal for an absolute targeted assay, due to the unstable peptide measurements across runs. One explanation is that CH3L1 is low-abundant in many patient categories used in testing and as controls, but more abundant in MS patients. This may explain why several discovery studies have found it regulated [5, 24, 25, 28] compared to controls and why we find high variations in this stability test using CSF from Control (OND) patients. Some peptides for CH3L1 were not far from being acceptable in terms of variation, having inter-day CV values between 20 and 30%. Due to the potential importance of this protein one could consider including PRM assays for these peptides, but then taking into account that the variation in the data is larger.

### Peptide digestion testing—most peptides show no increase after 16 h

Considering our goal of creating PRM assays able to measure absolute protein amounts, we found that it was crucial to examine the digestion status after the standard 16 h of trypsin incubation. This is not a standard test for PRM assays, but in order for an assay to come as close as possible to reflect the absolute protein amount, we found it essential. For most proteins, digestion times of 16 h were sufficient, in that no significant increase (or decrease) in peptide amount was observed after prolonged incubation. But there were a couple of peptides increasing also after 16 h of digestion, and nine proteins having some peptides passing and some failing the digestion test (Fig. 4b).

When examining the full profile of peptide amount (L/H) measured after various trypsin incubation times (Additional file 7: Fig. S1), we also note that certain peptides show a decrease already before 16 h of digestion. Although, no decrease beyond 20% was observed before 16 h, this may still indicate that some undesired peptide degradation is occurring already before 16 h. Such “fast digesting” peptides should ideally have shorter trypsin incubation times. For the peptides where the digestion was not completed after 16 h, one could envision longer digestion times in order to reach complete digestion, or consider testing other digestion conditions. However, applying individual trypsin digestion times and conditions for a large number of peptides in assays run over time is tedious and unrealistic. The peptides where the digestion was not complete after 16 h are not suitable for absolute quantification, but the assays could still be used for relative quantitation if trypsin incubation times are equally long between experiments. An alternative would be to search for other peptides better representing these proteins when using 16 h digestion time.

The overall results from the digestion testing show that each peptide can have specific physiochemical properties affecting the digestion process and demonstrate the necessity of obtaining peptide digestion profiles for each individual peptide. Another approach could be to use isotopically labelled proteins as internal standards, instead of peptides, in which case digestion variability would be adjusted for by the internal standard. This is however a quite costly approach.

### PRM RRMS vs control—confirmation of previously found changes

This small PRM study was designed to investigate whether the selected peptides could reproduce the previously reported quantitative differences between MS and controls. As we had merged some of the disease sub-categories when performing the initial CSF-PR search, it was not possible to find identical MS and control groups. We concluded that using relapsing–remitting MS (RRMS) and OND controls, was a good choice for this experiment.

Most of the peptides also passed this test, but seven peptides did not show the same significant difference between RRMS and OND as reported between MS and Non-MS in the CSF-PR publications [14], either because the difference was not significant or they showed the opposite change. One reason may be that the number of patients included in both the PRM study described here and the studies in CSF-PR is not large enough to eliminate the biological variation as a factor, creating false positive biomarker candidates. The patient groups used were also not identical, which could result in variation in differentially abundant proteins.

Another reason for the discrepancy may be that many of the studies in CSF-PR used depletion of high-abundant proteins, which on purpose was not performed in our PRM pilot experiment. Depletion could potentially affect the protein quantitation and thereby the resulting differentially abundant proteins as variation is introduced, as discussed above. This is particularly relevant if the number of patients is low. Even though the seven rejected peptides did not pass this test, they could still prove valuable upon more thorough testing with larger patient numbers. In the current study, the 37 peptides displaying similar differential abundance as previously reported were prioritized.

### Development of calibration curves—linearity down to the highest dilution point

Calibration curves were generated in rat plasma as it is a somewhat similar matrix to CSF and from a different species (non-human CSF was not possible to obtain), so that there would be no endogenous presence of analyte signal in the matrix, which would add to the spiked signal [40]. The calibration curves displayed a high degree of linearity down to the highest dilution point, with adjusted R^2^ values all over 0.99. Ideally, the calibration curves would include endogenous analyte concentrations so that the signal would be indistinguishable from the background, yielding a hockey-stick shape of the curve. The % CV of the replicates of the lowest endogenous peptide concentrations was less than 20% for all but one peptide, indicating that the limit of quantification (LOQ) was not reached for these. As the analyte concentrations rarely varies more than the span covered by the linear curve, the assays were deemed sufficient for our purposes, and the concentration span between the lowest and highest measured endogenous concentration will be used for absolute quantitation.

### Relevance for multiple sclerosis and other neurological disorders

The assays have been developed to monitor processes affected by MS, but through CSF-PR, we find that several of the protein candidates are also found changed in Alzheimer’s and Parkinson’s disease. This is the case for nine of the 21 proteins having peptides that passed all test (CD44 [41], Follistatin-related protein 4 [41], Secretogranin-2 [31, 42] ProSAAS [42], Neurexin-1 [31], Cadherin-13 [43], Kallikrein-6 [44], Amyloid-like protein 1 [44] and Ephrin type-A receptor 4 [44]). It indicates, not surprisingly, that many of the processes affected by MS are also affected by other neurological disorders, and are thus not specific to MS. Which in turn can mean that the diagnostic value of these particular proteins is limited, however they could still be very valuable as biomarkers for disease status, treatment effect and prognosis.

Validation in larger cohorts using the developed assays is necessary to determine the value of the proteins as biomarkers in a clinical setting. After validation of a subset of the proteins using PRM, one likely way to implement the measurement of the proteins into the clinic would be to develop ELISA-assays for the most valuable proteins. In the future the PRM-assays could perhaps also be used directly in the clinic.

It is also expected that these assays will be useful in shedding light on the disease status for other diseases where similar processes are affected. The remaining 12 proteins with peptides that passed all tests are however only changed in the MS categories in CSF-PR. These proteins may therefore be the most useful for diagnostic purposes and monitoring of processes occurring specifically in MS patients. For more details, the proteins can be searched and available data investigated in CSF-PR.

## Conclusion

In this study, we have developed 37 robust PRM peptide assays for 21 CSF proteins likely affected by MS. The selected proteins cover many of the pathways and processes recently reported to be affected in MS, but also in other neurological diseases such as Alzheimer’s and Parkinson’s disease. The peptides chosen as protein surrogates are quantifiable without the need for depletion, fractionation or enrichment prior to mass spectrometry. Due to the documented inter- and intra-day stability of the assays and the digestion stability, comparable quantitative values over time is expected. This allows for large-scale analyses of patient samples to reveal the relationship between the monitored MS-affected processes, disease progression and treatment response, and results from future large-scale patient analyses using these assays are expected to aid in treatment decisions.

These well-documented absolute quantitative assays could also be adopted by other laboratories and have the potential to generate comparable quantitative measurements between laboratories. To explore this potential future, inter-laboratory comparisons must be conducted.

We recommend that the presented workflow should be used as a general guideline for the development of targeted PRM biomarker assay in CSF, and consider this work to be a contribution towards standardizing CSF protein quantification allowing us to move from non-comparable data between single experiments to accumulation of reproducible quantitative data over time. In our view this is essential in order to enable the analyses of large enough patient cohorts to reveal disease-related changes in the CSF proteome related to disease status and progression.

## Materials and methods

### Biological material

#### Cerebrospinal fluid

Human cerebrospinal fluid (CSF) was obtained by diagnostic lumbar puncture, according to the standardized protocol for collection and biobanking [45]. Patients gave written consent and the study was approved by the Regional Committee for Medical Research Ethics of Western Norway. Various pools of CSF were used in the experiments described in this paper, mainly due to the limited availability of CSF samples to use for assay optimization and testing. Details about the pools can be found in Table 4 and in [5]. The same pools as in [5] were used for the MS vs. Control PRM study (MS and OND pools, here: Pools 3–8). The pool used for the DDA proteome depth and peptide stability experiment consisted of various OND patients (Pool 2), the pool used in the digestion test consisted of 3 OIND patients (Pool 1, all with myelitis).

**Table 4:** Overview of cerebrospinal fluid pools used in the various experiments

Name	#Patients each pool	Female/Male	Disease category	Average age	Used in experiment
Pool 1	3	2/1	OIND	35.3	Peptide digestion test
Pool 2	N/A	N/A	OND	N/A	DDA + peptide stability test
Pool 3–5	7	18/3	RRMS	36.8	PRM RRMS vs. control
Pool 6–8	7	18/3	OND	35.4	PRM RRMS vs. control

RRMS: Relapsing-remitting multiple sclerosis; OIND: Other inflammatory neurological diseases; OND: Other neurological diseases; DDA: data-dependent acquisition

#### Rat plasma

Rat plasma (P2516, Sigma Aldrich) was used to construct calibration curves for high-purity peptides. The purchased rat plasma contained lyophilized material derived from 1 ml pooled and filtrated rat blood with the addition of anticoagulant, 3.8% trisodium citrate. The concentration of the rat plasma was estimated by BCA, and diluted in 1xPBS to a final concentration of 0.5 µg/µl prior to trypsination.

### Literature curation using CSF-PR

We used CSF-PR (https://proteomics.uib.no/csf-pr) to extract biomarker candidates between MS and non-inflammatory control patients (Non-MS) as of August 2017. To specifically extract quantitative data relevant to this comparison, we first merged certain MS and control subcategories listed in CSF-PR as follows: RRMS (relapsing-remitting MS), CDMS (clinically definite MS) and CIS-MS (clinically isolated syndrome with conversion to MS) were merged to the general category “MS” and the subcategories OND (other neurologically controls), symptomatic controls, Non-MS, healthy and healthy controls were all merged to the general category “Non-MS”. In this way we identified protein data from all papers in the resource comparing MS to non-inflammatory controls. The protein table with the quantitative data from these studies contained thousands of proteins, so we applied some selection criteria by using the table filtering options in CSF-PR before exporting the protein list: (i) proteins quantified in at least two studies and (ii) having a CSF-PR score () of >=0.5 (50%) or < = − 0.5 (− 50%) according to the equation used in CSF-PR for summarizing overall reported protein regulation (see below, *×*100 for %), indicating that each protein was increased or decreased in at least 50% when averaging the results from all studies.

This resulted in 194 proteins, which were exported from CSF-PR, and further analysis was performed using Excel. To identify the most consistently changed proteins, we applied an additional criterion that (iii) proteins were found changed in the same direction (up or down) in at least two studies. This reduced the list to 133 proteins (Additional file 1: Table S1), representing the most promising and consistently reported biomarker candidates for MS. A separate list of proteins that were significantly changed between MS and Non-MS, but quantified in only one study in CSF-PR was also created (Additional file 2: Table S2).

### CSF sample preparation—general

Protein concentration in the CSF pools was measured by the QubitTM fluormeter (InvitrogenTM, Thermo Scientific) and the Qubit™ protein assay kit (InvitrogenTM, Thermo Scientific), following the manufacturers protocol. CSF samples were lyophilised at 30 °C in an Acid-Resistant CentriVapTM Concentrator System (LabconcoTM), and dissolved in 20 µl freshly made Urea solution (8 M Urea/20 mM methylamine (Sigma Aldrich)). All CSF samples were in-solution digested as previously described [4] using trypsin porcine (Promega, art. V5111) added to samples in a 1:50 relationship, and desalted using OASIS ^®^ HLB µElution plates 30 µm (Waters Corp, Millford, MA, USA) according to the manufacturer’s instructions. Samples were vacuum dried following desalting, and dissolved in 2% ACN, 0.1% TFA to a concentration of approximately 0.5 µg/µL for the MS analysis. About 0.5 µg were injected if not otherwise stated.

#### Preparation and spike-in of synthetic peptides

All isotopic labelled peptides (IS peptides) used as internal standards were purchased from Thermo Scientific at crude (unknown purity) and AQUA Ultimate (> 95% purity) quality for peptide testing and AQUA Ultimate quality only for calibration curves, whilst synthetic light peptides (SpikeTides) were acquired from JPT. Heavy labelled peptides have been C-terminally modified with 13C and 15 N isotope arginine or lysine. The synthetic heavy peptides were added to the samples before the desalting step, and the synthetic light peptides from JPT used to make calibration curves were added prior to digestion as they contain a tag that needs to be enzymatically released. Heavy peptides were spiked to the samples in an approximate 1:1 relationship between the heavy IS peptide and the endogenous analyte, which was estimated from initial peptide tests (data not shown). Notably, the lowest endogenous concentration was adjusted to 3 fmol/µg. Spike-in for calibration curve development is described under “calibration curve” section.

### PRM mass spectrometry—general for all PRM experiments

The separation of peptides was performed by an Ultimate™ 3000 RSLCnano System (Thermo Fisher Scientific™) with an Acclaim PepMap™ 100 trap column (diameter width at 75 µm × 2 cm nanoviper C18 column, with particle size 3 µm and length at 100 A) and 5 µL 0.1% TFA solution. Peptides were separated on an analytical column PepMapTM RSLC C18 (diameter width 75 µm × 50 cm, particle size at 2 µm and 100 A in length) with the combination of 95% solvent A (0.1% FA) and 5% solvent B (100% ACN, 0.1% FA) with a flow rate of 200 µl/min. The column gradient for peptide elution went from 0 to 5 min with 5% solvent B, then an increase at 5–5.5 min to 8% of solvent B, 5.5–140 min 35% B, 140–155 min 90% B and 155–170 min 90% B. At 170–175 min solvent B decreased to 5% B and held at 5% solvent B from 175 to 190 min. Column temperature was specified to be 35 °C, whilst the auto sampler had a temperature of 4 °C. Ionization of samples occurred with an Easy-Spray™ (Thermo Scientific) ion source, with a spray voltage at 1.8 kV. The capillary temperature was set at 250 °C, heater temperature at 350 °C, whilst the S-lens RF value were at 60. Sheath and auxiliary gases were not used. As a result of the ion source, samples were obtained in a positive ionization mode.

### Mass spectrometry analysis

The method duration was 195 min (runtime 10–175 min). The mass spectrometer was operated in PRM scheduled mode and switched between full scan MS1 between every 12th PRM MS2 scan. The instrument was controlled through Q Exactive HF Tune 2.4 and Xcalibur 3.0. MS1 spectra were acquired in profile mode in the scan range of 375–1500 m/z with resolution of 15,000, automatic gain control (AGC) target of 3e6, and a maximum injection time (IT) of 15 ms. The target peptides on the inclusion list were sequentially isolated for higher-energy collision dissociation (HCD) fragmentation and MS2 acquisition to a normalized HCD collision energy of 28%, target AGC value of 1e5, resolution R = 15 000, and IT of 100 ms. The precursor isolation window was set to 1.6 m/z with no isolation offset or dynamic exclusion. Lock-mass (445.12003 m/z) internal calibration was used.

### Skyline analysis

Skyline [29] was used for the creation of inclusion lists prior to PRM-mass spectrometry analysis and for data refinement of the PRM-mass spectrometry data. Skyline settings were overall kept at default, or updated depending on the parameters in the mass spectrometry analysis used to acquire data. Notably, structural modifications were specified with carbamidomethyl (C) and isotope modification “label: 13C(6) 15 N(2) (C-term K)” and label: “13C(6) 15 N(4) (C-term R)”. Both 2+ and 3+ charged precursors and b- and y-ions were investigated in the stability test experiment, while in the following (digestion, MS vs. OND and calibration curves), only 2+ precursors and y-ions were used, as these most often gave the best signal. Detailed Skyline settings for each experiment, e.g. the peptide and transition settings and filters, can be inspected in the Skyline documents uploaded to Panorama Public (https://panoramaweb.org/PRM_Assay_CSF.url).

The peak signal for each peptide was determined by the Skyline peak picking algorithm, and manually verified or re-integrated based on the fragment pattern of the peptide, elution profile and simultaneous retention time of the endogenous and the IS peptide. Spectral libraries from CSF samples generated on the same Q Exactive HF instrument were used as a reference to make sure the correct peak for the various peptides were chosen. The three fragments with the highest intensity, low interference, and mass error less than 10 ppm was selected for quantitation. Additional file 13: Fig. S4A and B shows examples of typical transitions used in the assay. All other transitions can be inspected in Panorama Public (https://panoramaweb.org/PRM_Assay_CSF.url) where the Skyline documents from all experiments can be downloaded.

Notably, for most peptides, one to three of the transitions were significantly more intense compared to the rest, only the top three where therefore chosen for quantification. A typical example of this is illustrated in Additional file 13: Fig. S4C and D. For some peptides in certain tests or replicates, only two transitions were used for quantitation, due to missing data or bad peaks in specific replicates. These were mainly from very low abundant proteins and/or from peptides with only low intensity transitions. The area under the curve, excluding background, were summed to give one peak area value for each peptide.

Furthermore, the endogenous peak area was divided by the peak area of the heavy internal standard peptide to generate a ratio to standard which was used for quantitation. From Skyline, a.csv file was exported containing the quantitative data needed for follow-up processing in Microsoft Excel or R. To determine the difference between the two patient groups in the final PRM experiment, an unpaired two tailed, homoscedastic student’s *t* test was performed using Microsoft Excel. A *p*-value of ≤ 0.05 was used to determine a significant difference.

### CSF protein depth investigation

We tested the identification of CSF proteins from a 20 µg un-depleted CSF sample (pool 2) subjected to peptide fractionation into 11 fractions following trypsin digestion (as described above). Peptide fractionation was performed by mixed mode reversed phase-anion exchange chromatography (MM) [18] on a Promix MP column (MP10.250.0530, 1.0 × 250 mm, 5 μm, 300 Å, Sielc Technologies), as previously described [33].

### Data dependent acquisition mass spectrometry analysis

Approximately 0.5 μg of peptides from each fraction was injected into the same LC system, trap column and mass spectrometer as described above. However, a 25 cm analytical column (PepMap RSLC, 25 cm × 75 μm i.d. EASY-spray column, packed with 2 μm C18 beads (Thermo Scientific)) was used (flow rate of 0.250 μL/min). Solvent A and B was the same as above as was the other MS general instrumental parameters related to ionization, voltage, temperature etc.

The mass spectrometer was operated in data-dependent acquisition mode to automatically switch between full scan MS1 and MS2 acquisition. The method duration was 120 min (runtime 8-105 min). The instrument was controlled through Q Exactive HF Tune 2.4 and Xcalibur 3.0. MS spectra were acquired in the scan range of 375–1500 m/z with resolution of 60 000, automatic gain control (AGC) target of 3e6, and a maximum injection time (IT) of 25 ms. The 12 most intense eluting peptides above intensity threshold 5e4, and charge states two or higher, were sequentially isolated for higher-energy collision dissociation (HCD) fragmentation and MS2 acquisition to a normalized HCD collision energy of 28%, target AGC value of 1e5, resolution R = 60,000, and IT of 110 ms. The precursor isolation window was set to 1.6 m/z with an isolation offset of 0.3 m/z and a dynamic exclusion of 20 s. Lock-mass (445.12003 m/z) internal calibration was used and isotope exclusion was active.

### Data processing

All raw files were converted to mgf using ProteoWizard [46] and searched using X! Tandem [47], MyriMatch [48] and MS Amanda [49] via SearchGUI (v2.1.3) [50] against the homo sapiens complement of the UniProt/SwissProt reviewed database downloaded October 2015 (20 196 entries) [17] with the reversed version of every sequence added as decoys. The search settings were: carbamidomethylation of C; oxidation of M as variable modification; trypsin as enzyme with a maximum of two missed cleavages; precursor charge 2–5; peptide length 6–30; precursor mass tolerance 10 ppm and fragment mass tolerance 0.005 Dalton. All other settings were left as the defaults. The search engine results were combined and assembled in PeptideShaker [51] (v1.1.2). Hits were thresholded to retain only the best scoring until a false discovery rate (FDR) of 1% was reached, estimated using the distribution of target and decoy hits [52].

### Peptide stability test

To test the intra- and inter-day variability of measurements for the peptides, we analysed aliquots from the same CSF samples (pool of OND patients) at two different time points at the same day across 5 days. Two 10 µg aliquotes of CSF-pool 2 were in-solution digested, spiked, desalted, dried and stored in − 20 °C. This was repeated on five different days, and all samples were analysed by PRM MS as described above. Data was inspected and refined in Skyline as described above, and in Excel, the intra- and inter-day variation for each peptide (2+ and 3+ separately) was calculated on the exported total area ratio (light/heavy). This value for each peptide was compared between samples prepared on the same day (intra-day) and between each of the three sample sets (inter-day). Peptides with intra- and inter-day CV less than 20% was considered reproducible.

### Peptide digestion test

Fifteen 10 µg aliquots of CSF-pool 1 was in-solution digested, spiked, and desalted as described above. Trypsination was however, stopped at five different time points (1, 5, 16, 24 and 30 h), and three replicates was stopped at each time point. This experiment was repeated three times (across three different weeks). Data was refined in Skyline, as described above and the percentage change in ratio to standard was calculated for each peptide between 16 to 24 h and 16 to 30 h. A peptide was considered stably trypsinated after 16 h if the percentage change from 16 to 14 h, and 16 to 30 h was less than 20%. Individual protein plots showing ratios at all time points for all peptides were generated using R (http://www.R-project.org). The plots were generated using the graphics package ggplot2 (https://ggplot2.tidyverse.org) (Additional file 7: Fig. S1).

### PRM RRMS vs control

Samples from six CSF-pools were used in this experiment. The samples were crude 100 µg aliquotes from the experiment described in [5], which was three pools of MS patients and three pools of OND patients (pools 3-8). The samples were purified and concentrated using 3 kDa ultracentrifugation filters as described in [53], before in-solution digestion and Oasis desalting as described above, except a 10 mg plate was used, as in [53], due to the high protein amount (100 µg). The eluate after the desalting was divided to 5 µg aliquots and two aliquots (replicates) from each pool was used for this experiment. The aliquots were spiked with heavy peptides, dried and dissolved in 2% ACN, 0.1% TFA to approximately 0.5 µg/µL. Approximately 1 µg sample was injected for MS analysis and analysed by PRM as described, except MS2 resolution was 30,000. The ratio to standard was used to calculate fold change and significance between groups.

### Development of calibration curves

#### Calibration curve generation in rat plasma

The calibration curves were made in rat plasma by preparing a dilution series of synthetic normal mass (light) peptides based on the estimated endogenous concentration of each peptide in CSF Pool 2. An 11-point dilution curve was centered around this estimate, so that the varying analyte spanned 32 times the endogenous concentration and 32 times less than the estimated endogenous concentration. The dilution series was prepared for the synthetic light peptides for analysis in rat plasma (dilutions prepared in 8 M Urea/20 mM methylamine directly prior to trypsination), Additionally, a mix of heavy AQUA peptides in levels 1:1 with endogenous peptide was generated in 5% ACN. Eleven 10 µg aliquots of rat plasma were added synthetic light peptides in different dilutions. In addition, one sample without added light peptides and was used as a blank. The twelve samples were in-solution trypsin digested, and equal amounts of the AQUA heavy peptides mix were added to each sample prior to desalting. This procedure was performed in trypsinated triplicates.

#### Mass spectrometry analysis

For the calibration curve experiment, some optimized parameters were used in the PRM analysis. Peptides were separated on an analytical column PepMapTM RSLC C18 (diameter width 75 μm × 25 cm, particle size at 2 μm and 100 A in length) with the combination of 95% solvent A (0.1% FA) and 5% solvent B (100% ACN, 0.1% FA) with a flow rate of 250 μl/min. The column gradient for peptide elution went from 0 to 5 min with 5% solvent B, then an increase at 5–5.5 min to 7% of solvent B, 5.5–65 min 22% B, 65–87 min 35% B, 87–92 min 90% B and 92–102 min with 90% B. At 102–105 min solvent B decreased to 5% solvent B and held a 5% solvent B from 105 to 120 min. The method duration for calibration curve runs was 120 min (runtime 10–110 min). The mass spectrometer was operated and MS spectra acquired as described above for PRM analysis, except MS2 spectra were acquired with optimized collision energies, resolution R = 60,000 at 200 m/z, IT of 118 ms, AGC target value at 2e5 and precursor isolation window was set to 0.7 m/z. All other parameters related to the LC and MS instrumentation and settings were as described above for general PRM experiments.

#### Calibration curve development in R

Following data refinement in Skyline, the ratio to standard values were exported for analysis in the programming language R (http://www.R-project.org). For the peptides measured in rat plasma, the measured ratio to standard was multiplied with the spike-in level to give the measured concentration at each dilution point and was plotted against the theoretical concentration. Notably, as more variation is common in the high concentration measurements, the linear regression was weighted with 1/sd^2 to limit the impact of the points with the highest variability on the regression equation [40]. The slope, intercept and the lowest and highest theoretical concentration points of the linear curve was exported. The plots were generated using the graphics package ggplot2 (https://ggplot2.tidyverse.org).

## Supplementary information

**Additional file 1: Table S1.** 133 proteins significantly changed (=/± 50%) between MS and Non-MS from CSF-PR. Quantified in minimum two studies, and changed in same direction in minimum two studies.

**Additional file 2: Table S2.** 287 proteins significantly changed between MS and Non-MS, but that were quantified in only one study in CSF-PR.

**Additional file 3: Table S3.** 120 proteins from Table S1 found in the DDA protein depth experiment.

**Additional file 4: Table S4.** All 1194 identified proteins from DDA protein depth experiment.

**Additional file 5: Table S5.** Data from the peptide stability experiment.

**Additional file 6: Table** **S6.** Data from the trypsin digestion experiment.

**Additional file 7: Fig.** **S1**. Digestion profile plot across all time points for all peptides.

**Additional file 8: Fig.** **S2.** Scatter plots comparing peptide amount (ratio L/H) at all times points tested in the digestion experiment.

**Additional file 9: Table** **S7.** Data from the PRM RRMS vs. OND experiment.

**Additional file 10: Table S8.** Full table of all tested peptides with essential results from each test and whether they passed or failed the tests.

**Additional file 11: Table** **S9.** Overview of the tests for stability, digestion and MS vs. OND experiments, for the 25 proteins. The colours and numbers refer to the peptides passing the specific test. Green: protein passed with two or more peptides; yellow: passed with one peptide; and, red: either no peptides passed (0) or none were tested (-).

**Additional file 12: Fig.** **S3.** Calibration curves for 17 assay peptides that passed all quality control tests.

**Additional file 13: Fig.** **S4.** Representative transition peaks from the Skyline analysis. **A** and **B** show typical examples of used transitions. The transition intensity, integration limits, retention time and mass error (ppm) is illustrated. **C** and **D** show examples of how 1-3 transitions were often much higher than the rest. Peak smoothing (Savitzky-Golay) was used in Skyline, which notably does not affect the quantification.

## Data Availability

The mass spectrometry proteomics data from the DDA experiment have been deposited to the ProteomeXchange Consortium via the PRIDE [54] partner repository with the dataset identifier PXD013449 and 10.6019/pxd013449. All other mass spectrometry data are targeted PRM data, and Skyline documents and raw files are available through Panorama Public [55] at https://panoramaweb.org/PRM_Assay_CSF.url and at ProteomeXchange with dataset identifier PXD017281.

## Abbreviations

ACN: Acetonitrile
AD: Alzheimer’s disease
AGC: Automatic gain control
CDMS: Clinically definite multiple sclerosis
CH3L1: Chitinase-2-like protein 2
CIS-MS: Clinically isolated syndrome with conversion to multiple sclerosis
CSF: Cerebrospinal fluid
CSF-PR: CSF Proteome Resource
CV: Coefficient of variation
DDA: Data dependent acquisition
FDR: False discovery rate
HCD: Higher-energy collision induced dissociation
IS: Internal standard
IT: Injection time
LC: Liquid chromatography
LOQ: Limit of quantitation
MM: Mixed-mode reversed phase anion exchange
MS: Mass spectrometry
MS: Multiple sclerosis
OIND: Other inflammatory neurological diseases
OND: Other neurological diseases
PD: Parkinson’s disease
PRM: Parallel reaction monitoring
RRMS: Relapsing–remitting multiple sclerosis
SRM: Selected reaction monitoring

## Availability of data and materials

The .raw files and datasets supporting the conclusions of this article are available in the PRIDE repository (ID: PXD013449 and PXD017281) and in Panorama Public (https://panoramaweb.org/PRM_Assay_CSF.url).
