# Supplementary material for: Development of robust targeted proteomics assays for cerebrospinal fluid biomarkers in multiple sclerosis
Source: Clin Proteomics. 2020 Sep 18;17:33. doi: 10.1186/s12014-020-09296-5 (PMC7499868; doi:10.1186/s12014-020-09296-5)
Supplement: Supplementary file 7 — Additional file 7: Fig. S1. Digestion profile plot across all time points for all peptides. [file 12014_2020_9296_MOESM7_ESM.pdf]

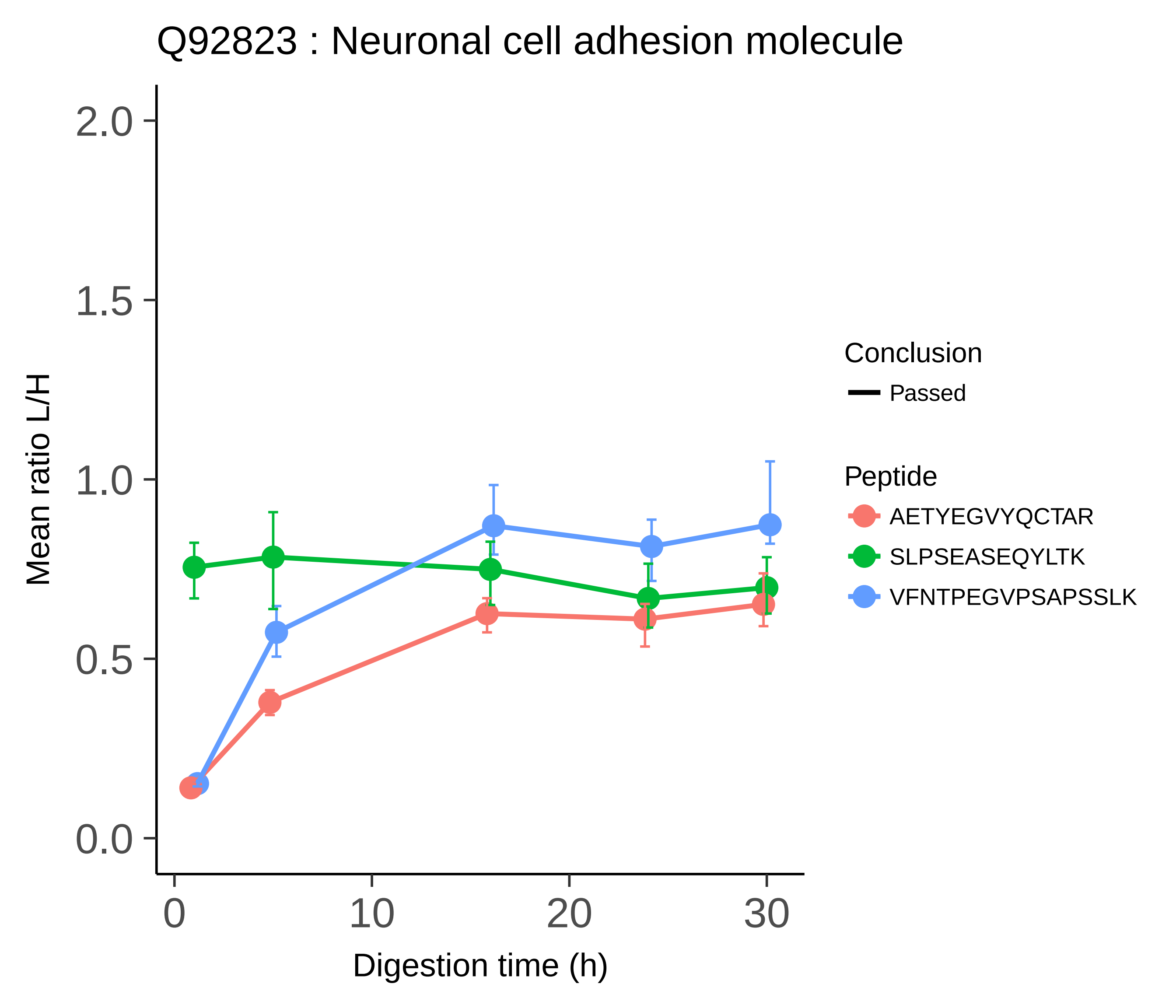
Q92823 : Neuronal cell adhesion molecule
Mean ratio L/H
2.0
1.5
1.0
0.5
0.0
0
10
20
30
Digestion time (h)
Conclusion
Passed
Peptide
AETYEGVYQCTAR
SLPSEASEQYLTK
VFNTPEGVPSAPSSLK

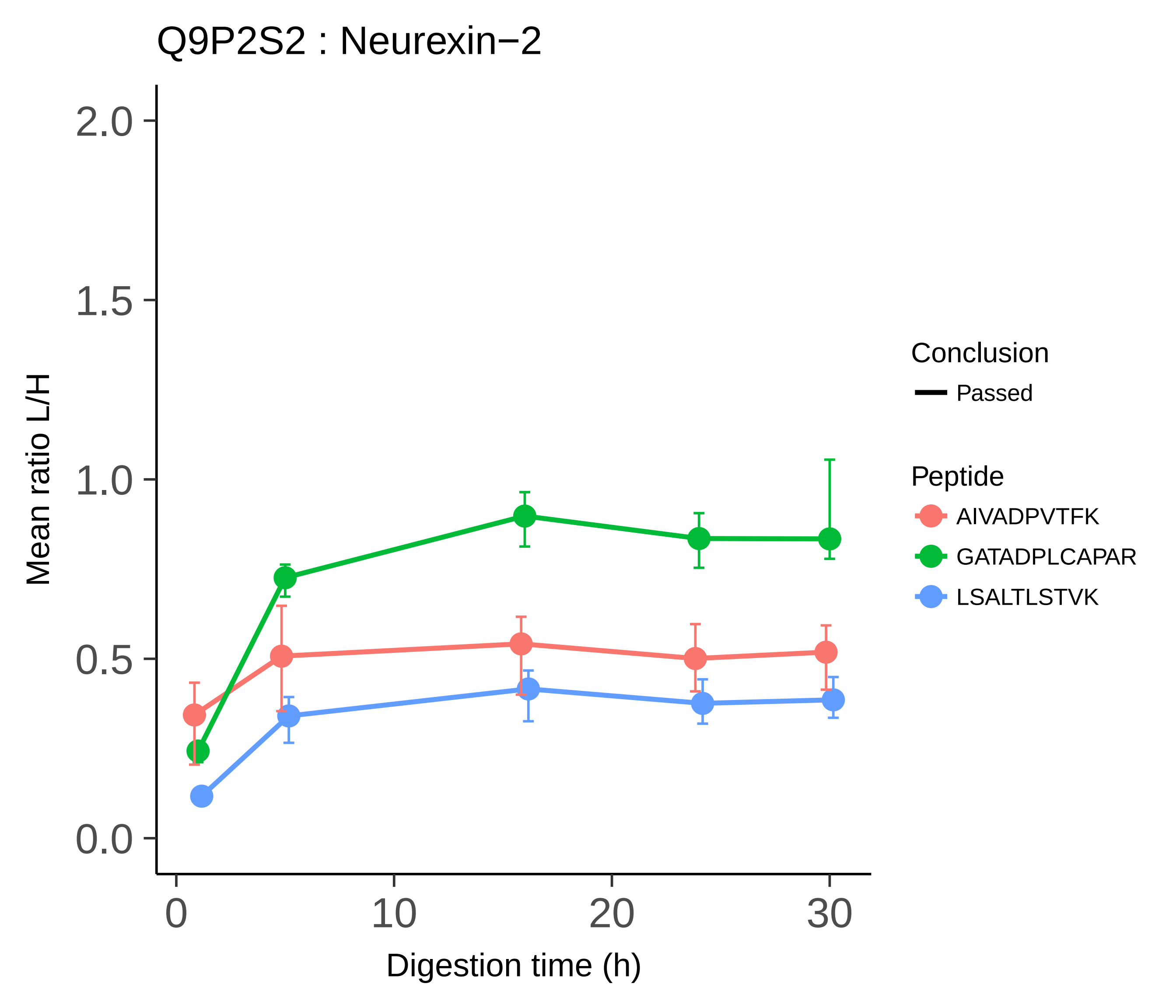

Q9P2S2 : Neurexin−2
Mean ratio L/H
0.0
0.5
1.0
1.5
2.0
0
10
20
30
Digestion time (h)
Conclusion
Passed
Peptide
AIVADPVTFK
GATADPLCAPAR
LSALTLSTVK

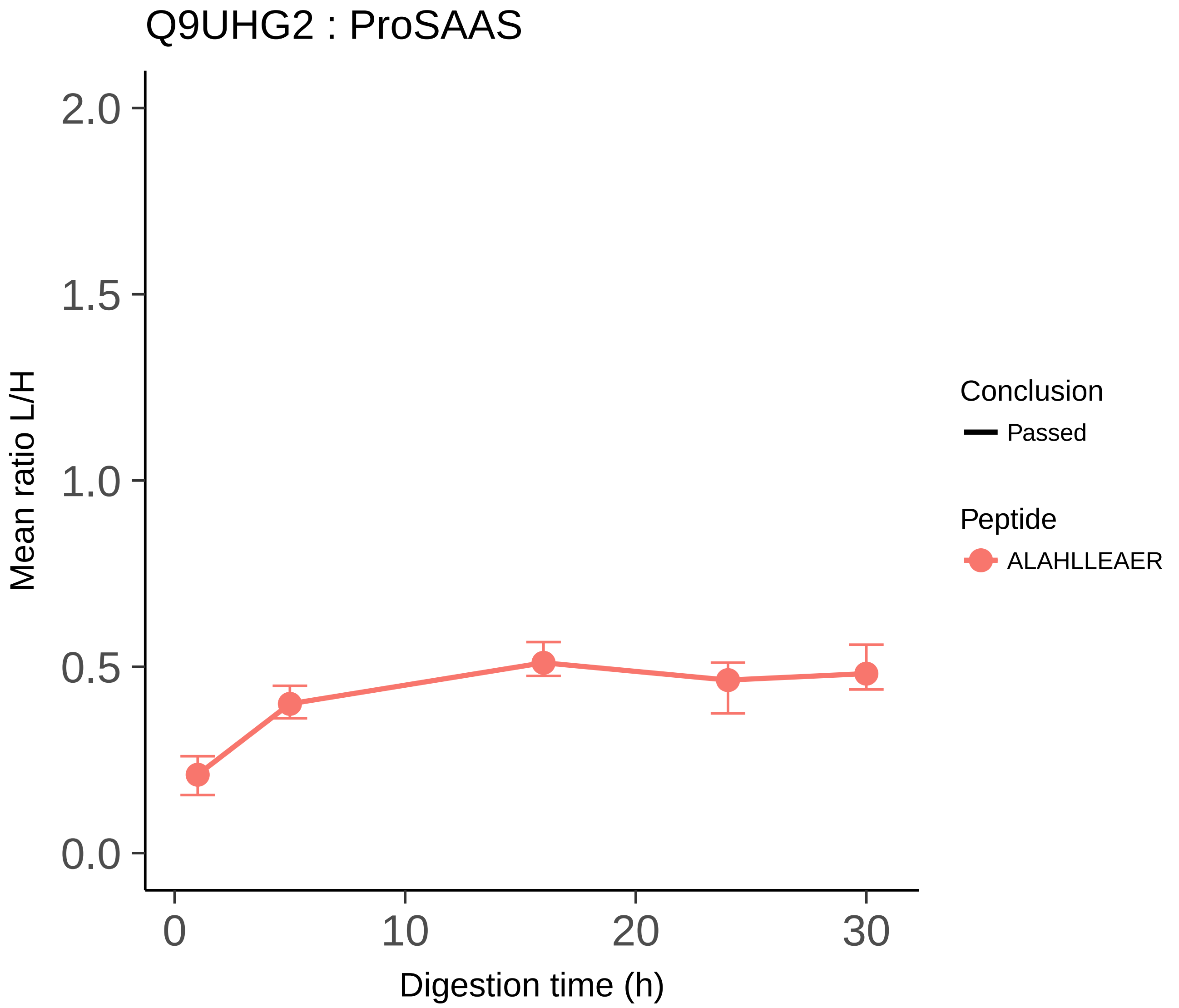
Q9UHG2 : ProSAAS
Mean ratio L/H
2.0
1.5
1.0
0.5
0.0
0
10
20
30
Digestion time (h)
Conclusion
Passed
Peptide
ALAHLLEAER

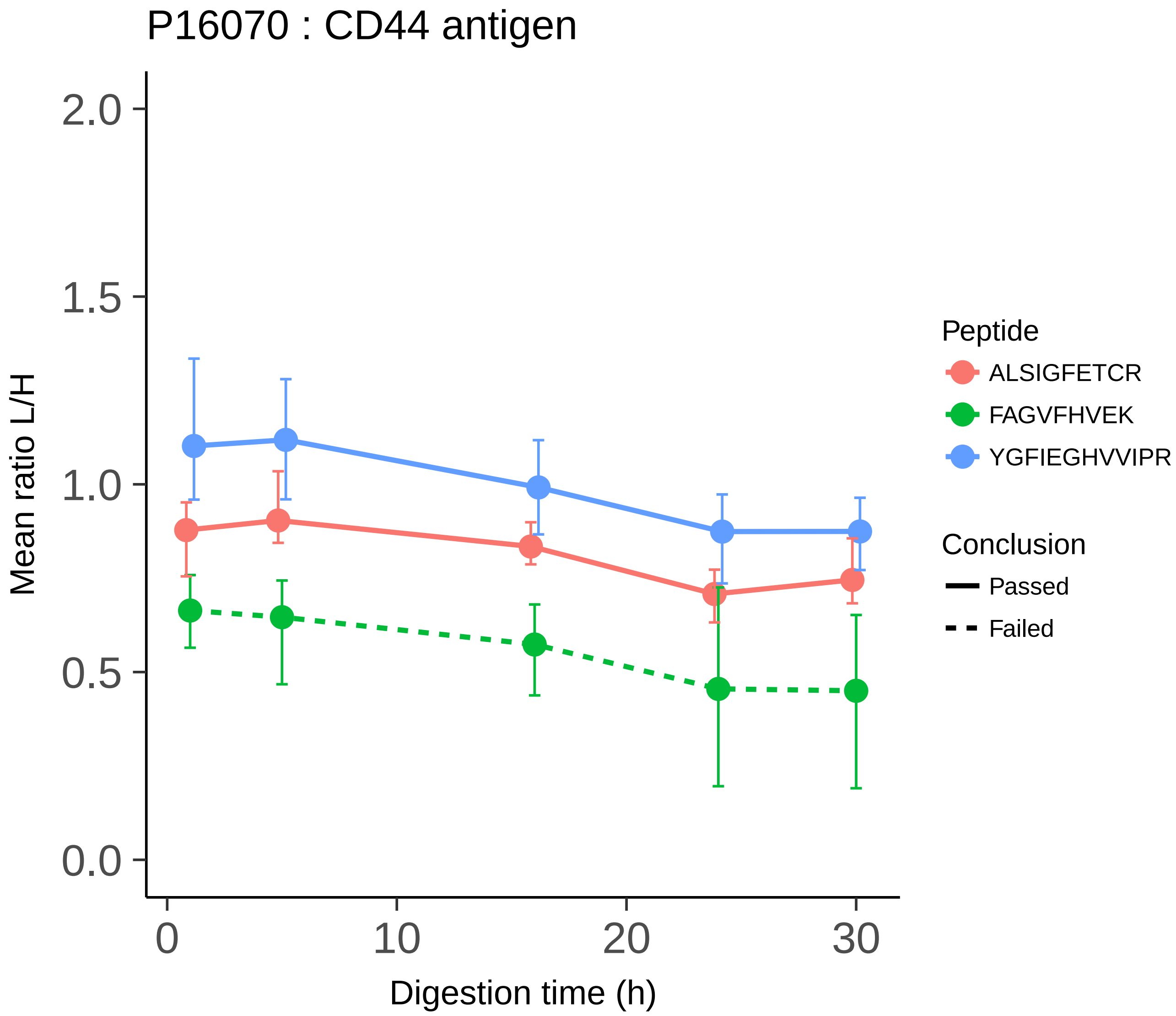

P16070 : CD44 antigen
Mean ratio L/H
Digestion time (h)
0.0
0.5
1.0
1.5
2.0
0
10
20
30
Peptide
ALSIGFETCR
FAGVFHVEK
YGFIEGHVVIPR
Conclusion
Passed
Failed

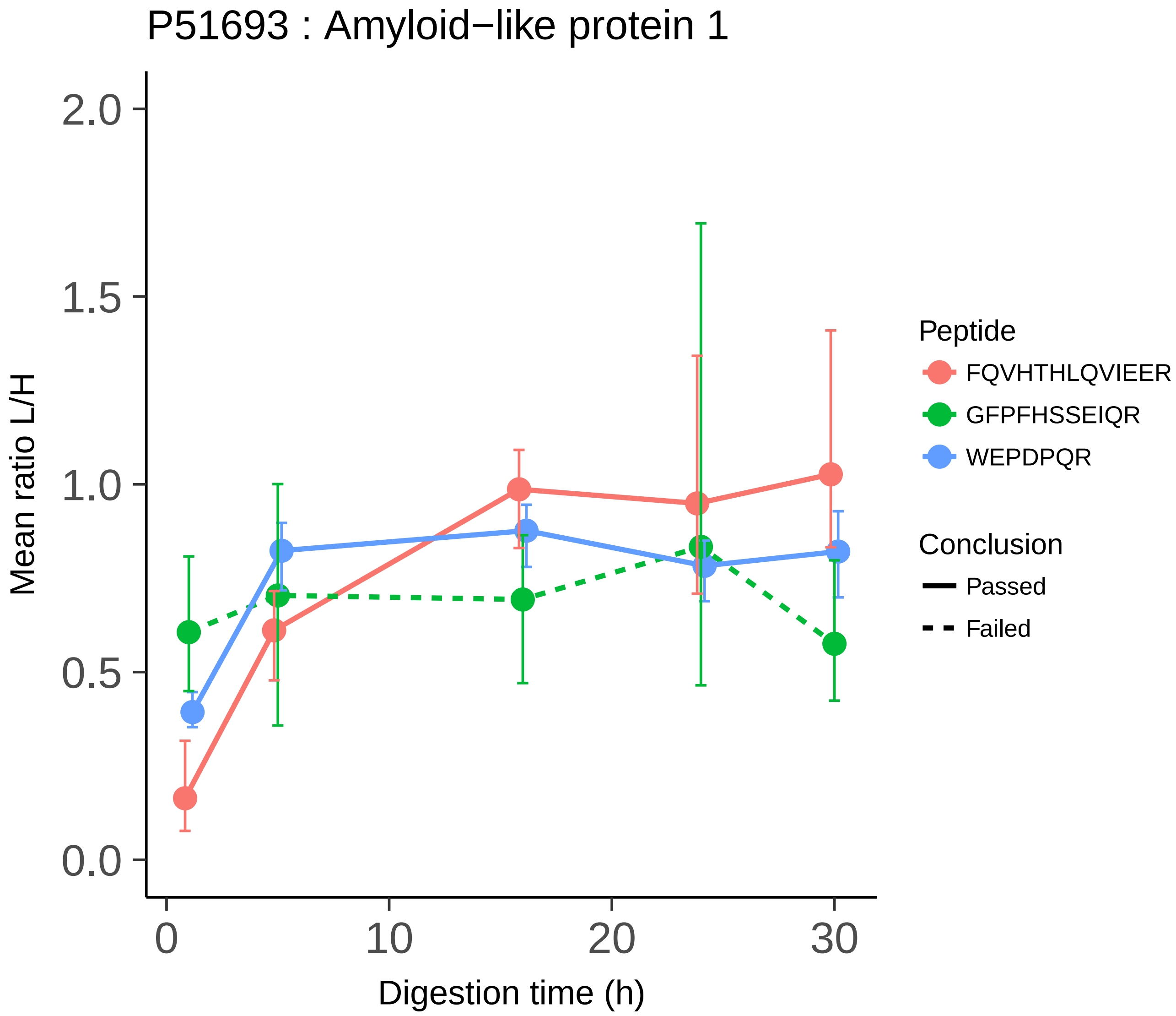
P51693 : Amyloid−like protein 1
Mean ratio L/H
0.0
0.5
1.0
1.5
2.0
0
10
20
30
Digestion time (h)
Peptide
FQVHTHLQVIEER
GFPFHSSEIQR
WEPDPQR
Conclusion
Passed
Failed

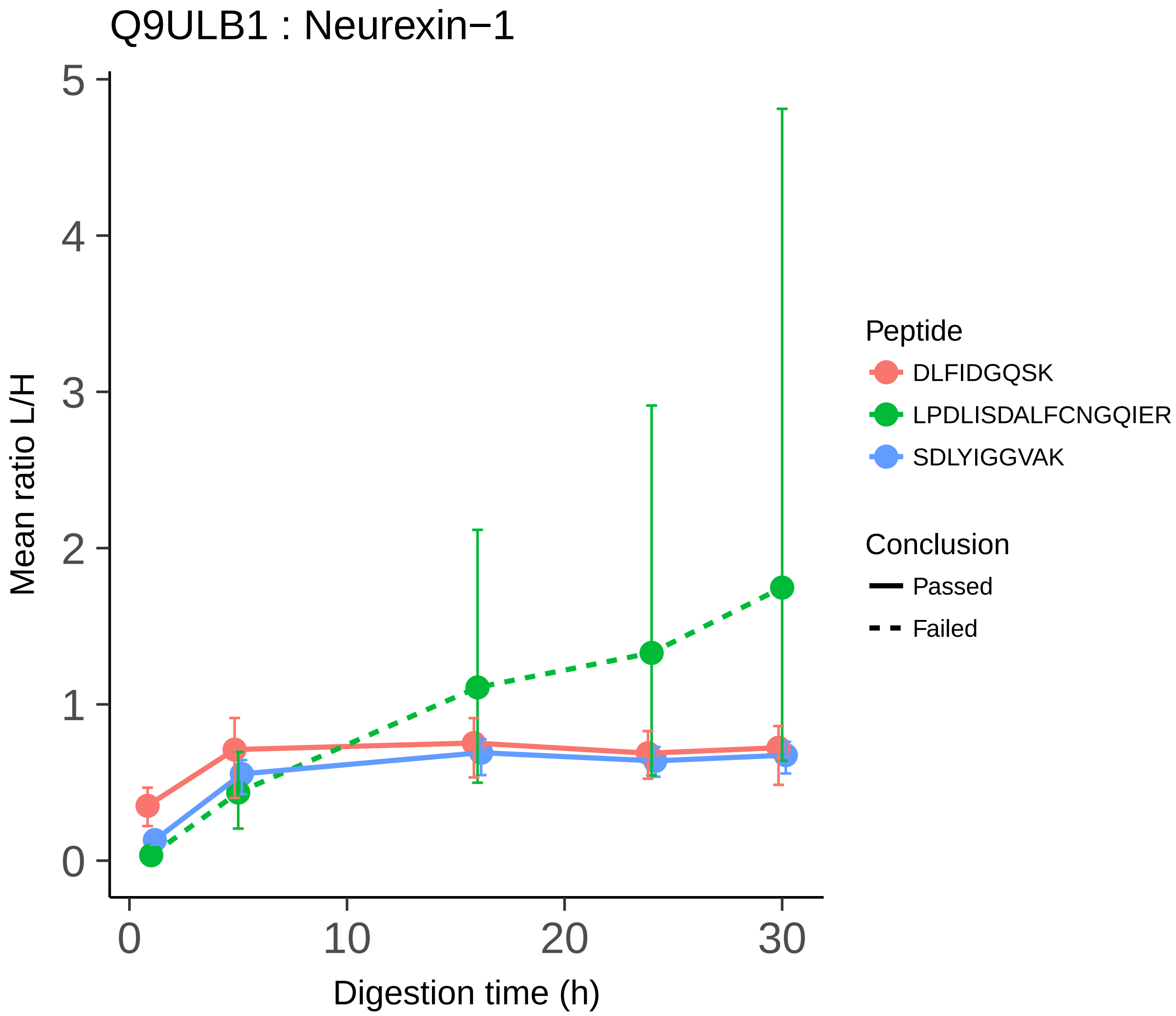
Q9ULB1 : Neurexin−1
Mean ratio L/H
0
1
2
3
4
5
0
10
20
30
Digestion time (h)
Peptide
DLFIDGQSK
LPDLISDALFCNGQIER
SDLYIGGVAK
Conclusion
Passed
Failed

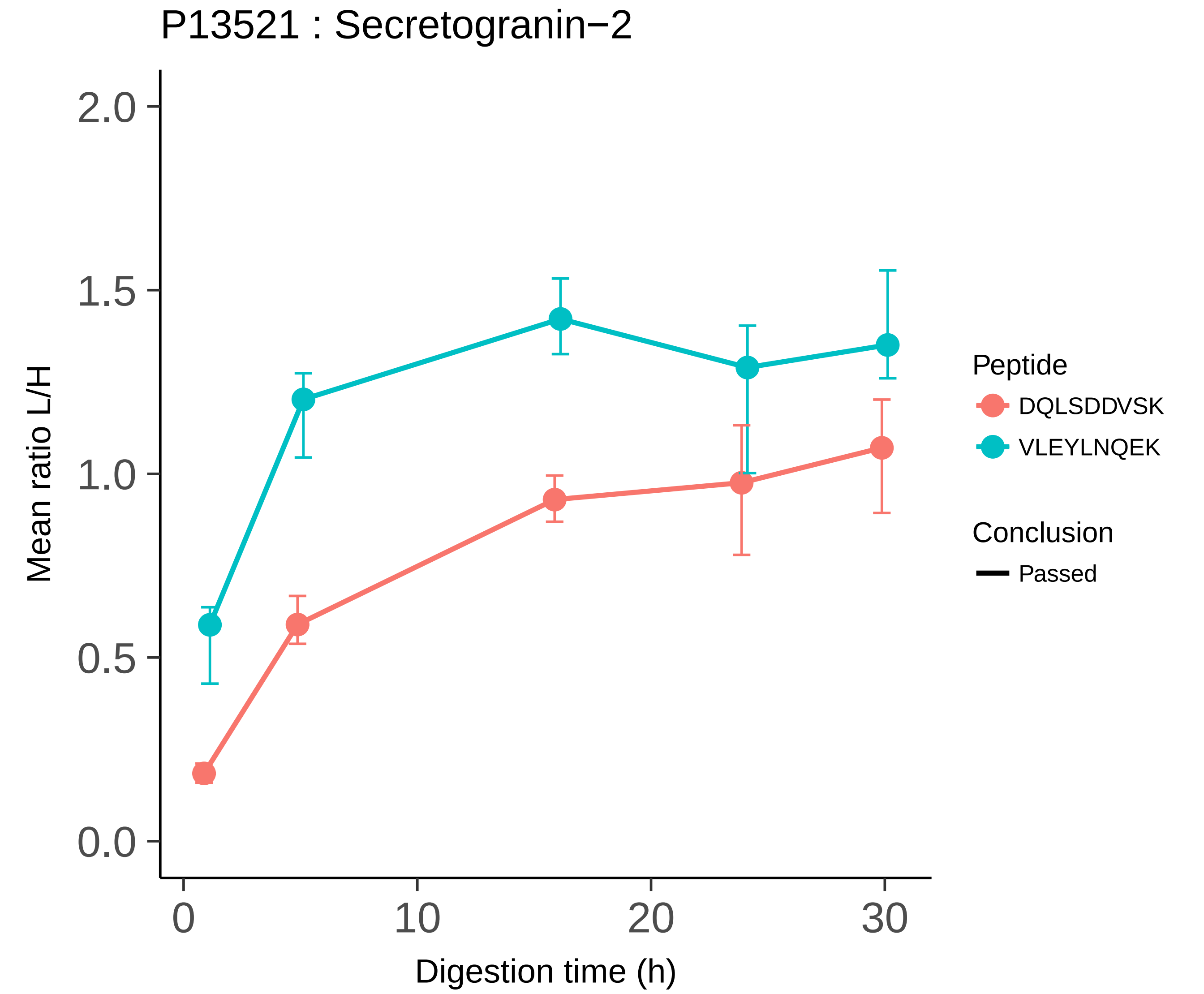
P13521 : Secretogranin−2
Mean ratio L/H
2.0
1.5
1.0
0.5
0.0
0
10
20
30
Digestion time (h)
Peptide
DQLSDDVSK
VLEYLNQEK
Conclusion
Passed

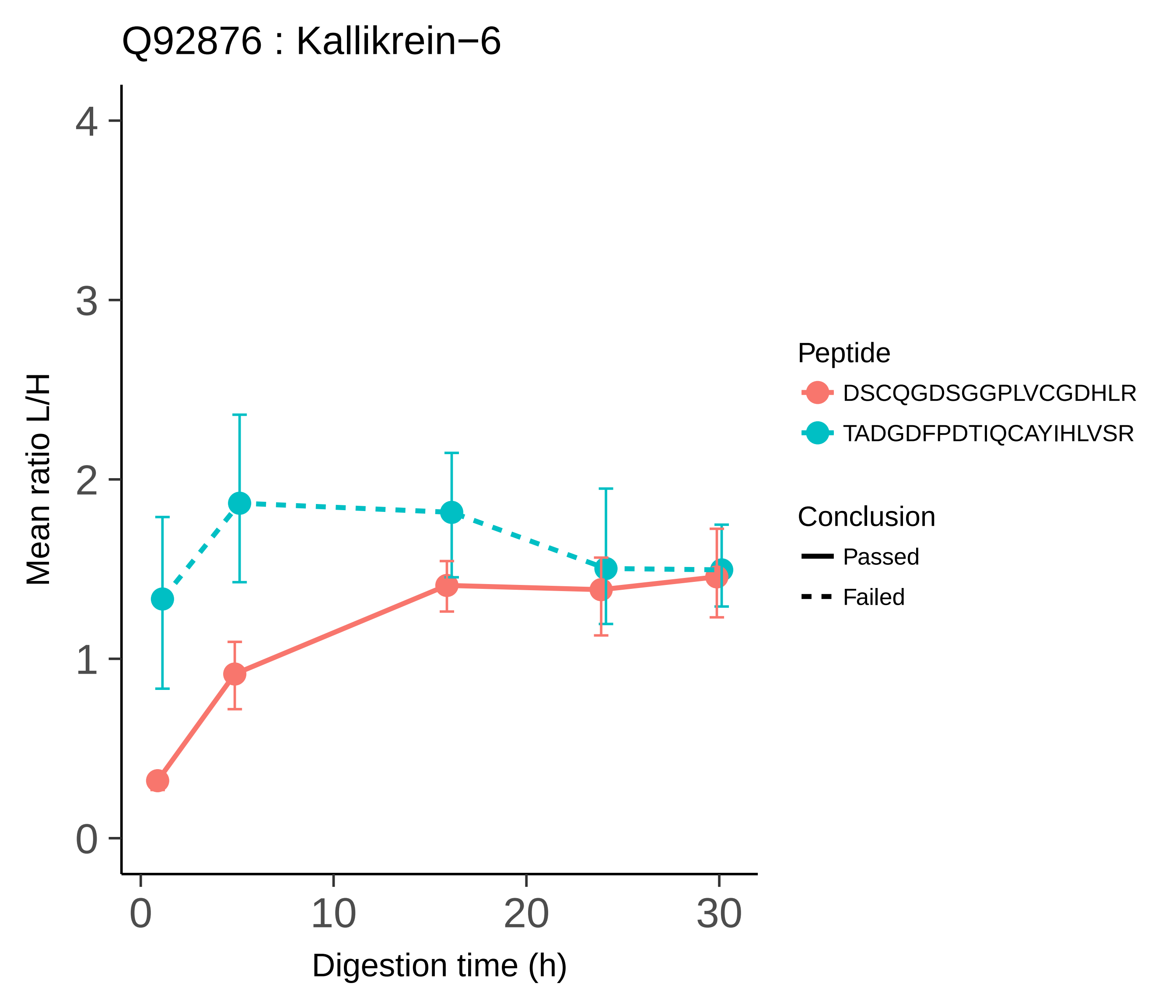
Q92876 : Kallikrein−6
Mean ratio L/H
Digestion time (h)
0
1
2
3
4
0
10
20
30
Peptide
DSCQGDSGGPLVCGDHLR
TADGDFPDTIQCAYIHLVSR
Conclusion
Passed
Failed

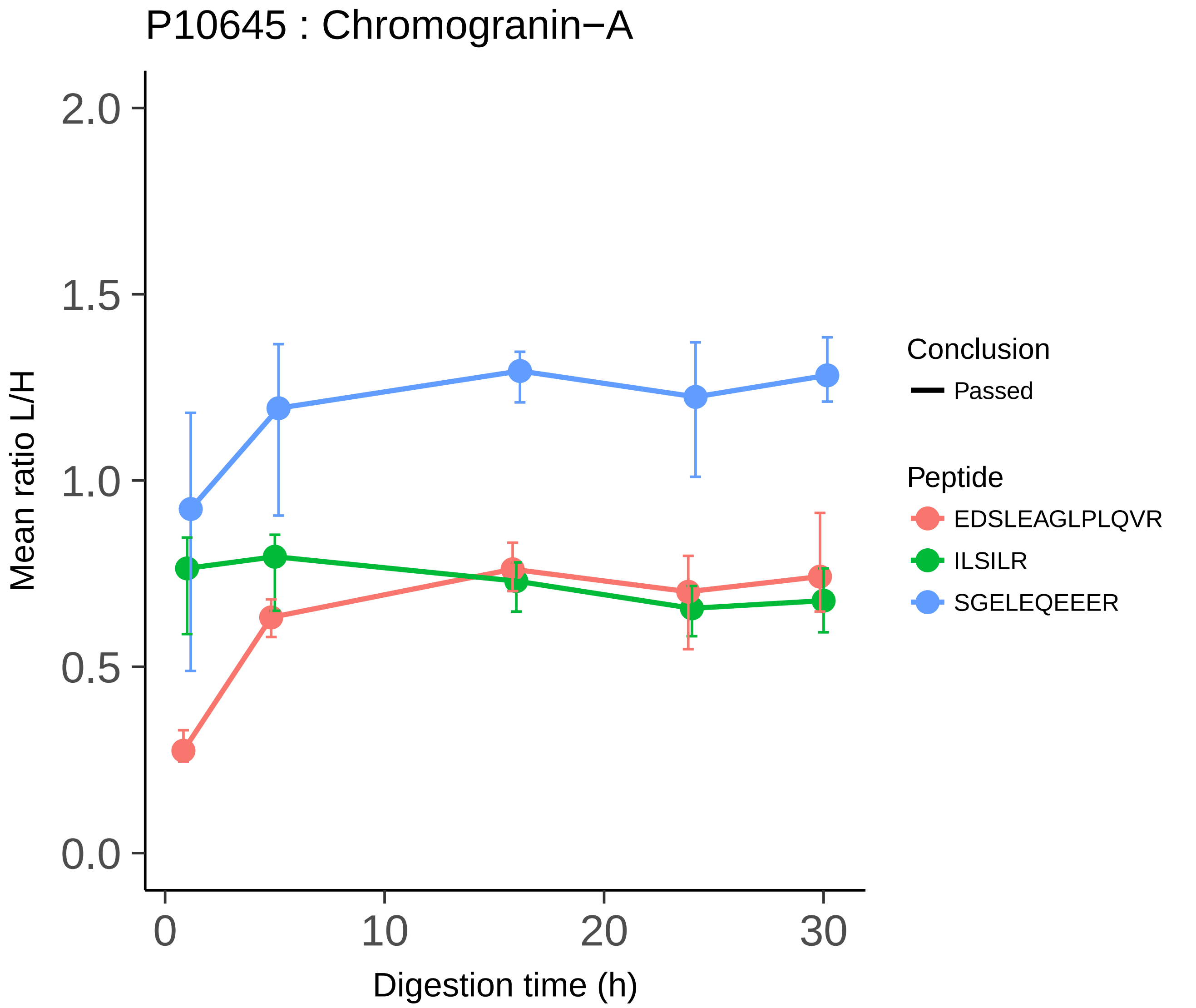
P10645 : Chromogranin−A
Mean ratio L/H
2.0
1.5
1.0
0.5
0.0
0
10
20
30
Digestion time (h)
Conclusion
Passed
Peptide
EDSLEAGLPLQVR
ILSILR
SGELEQEEER

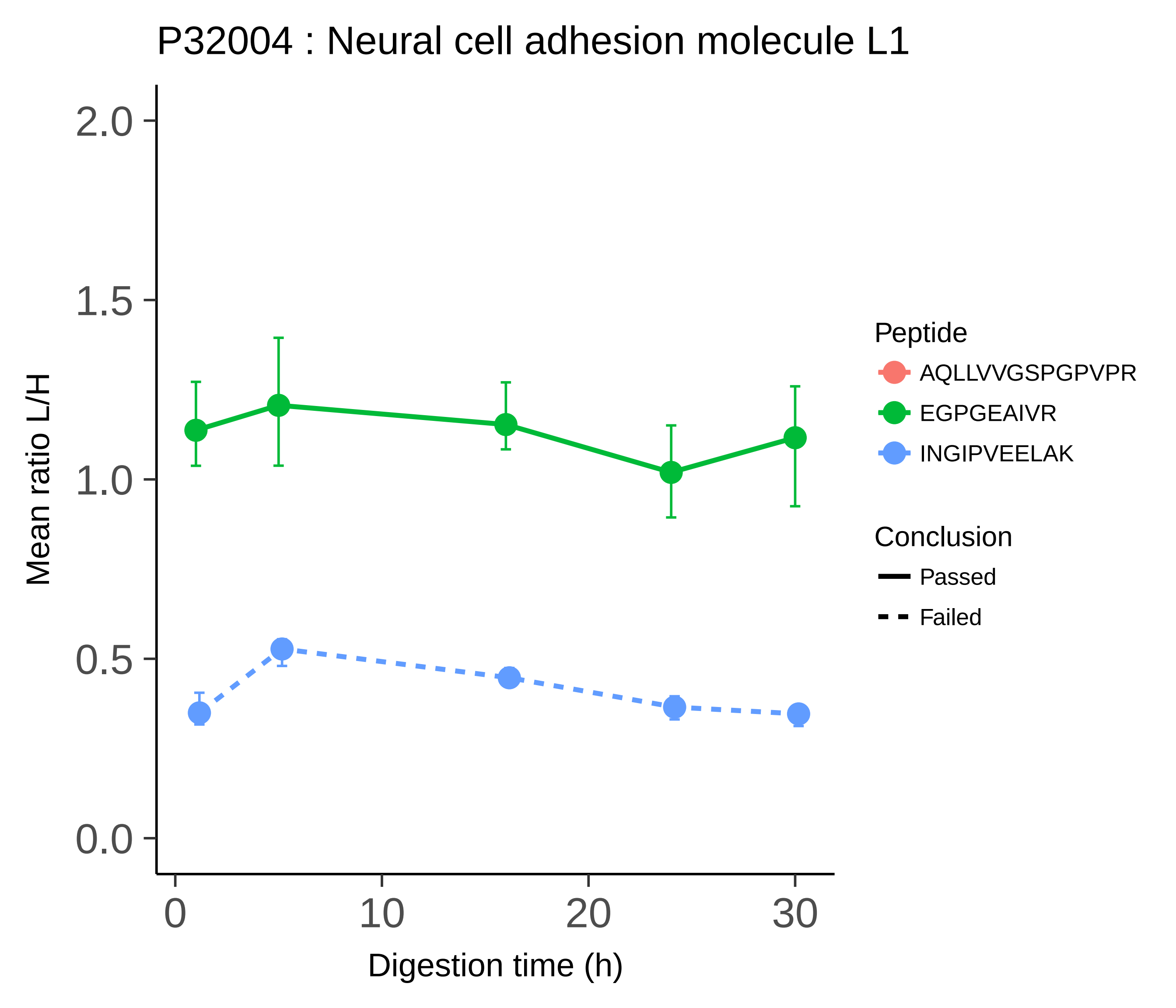

P32004 : Neural cell adhesion molecule L1
Mean ratio L/H
2.0
1.5
1.0
0.5
0.0
0
10
20
30
Digestion time (h)
Peptide
AQLLVVGSPGPVPR
EGPGEAIVR
INGIPVEELAK
Conclusion
Passed
Failed

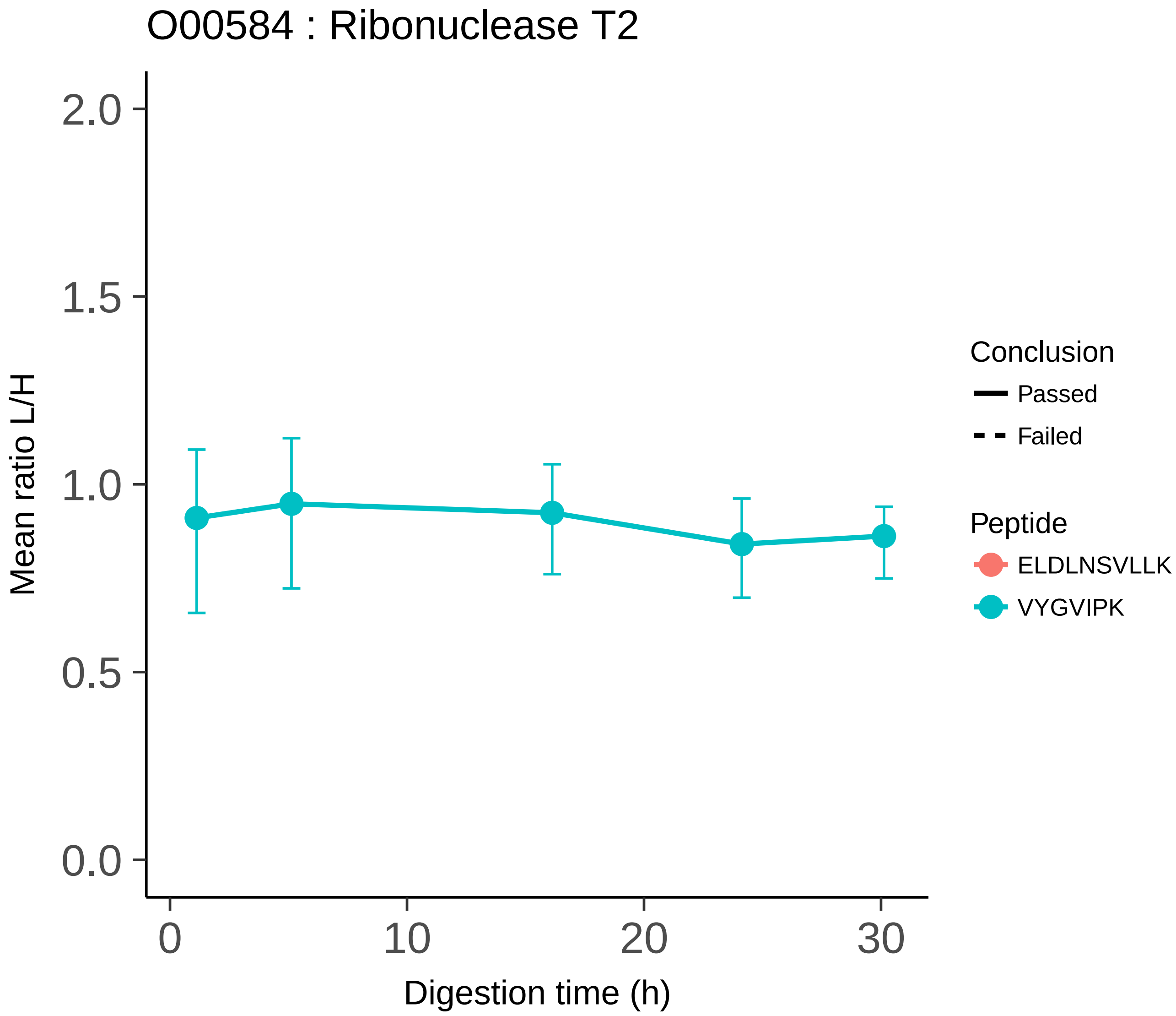
O00584 : Ribonuclease T2
Mean ratio L/H
2.0
1.5
1.0
0.5
0.0
0
10
20
30
Digestion time (h)
Conclusion
Passed
Failed
Peptide
ELDLNSVLLK
VYGVIPK

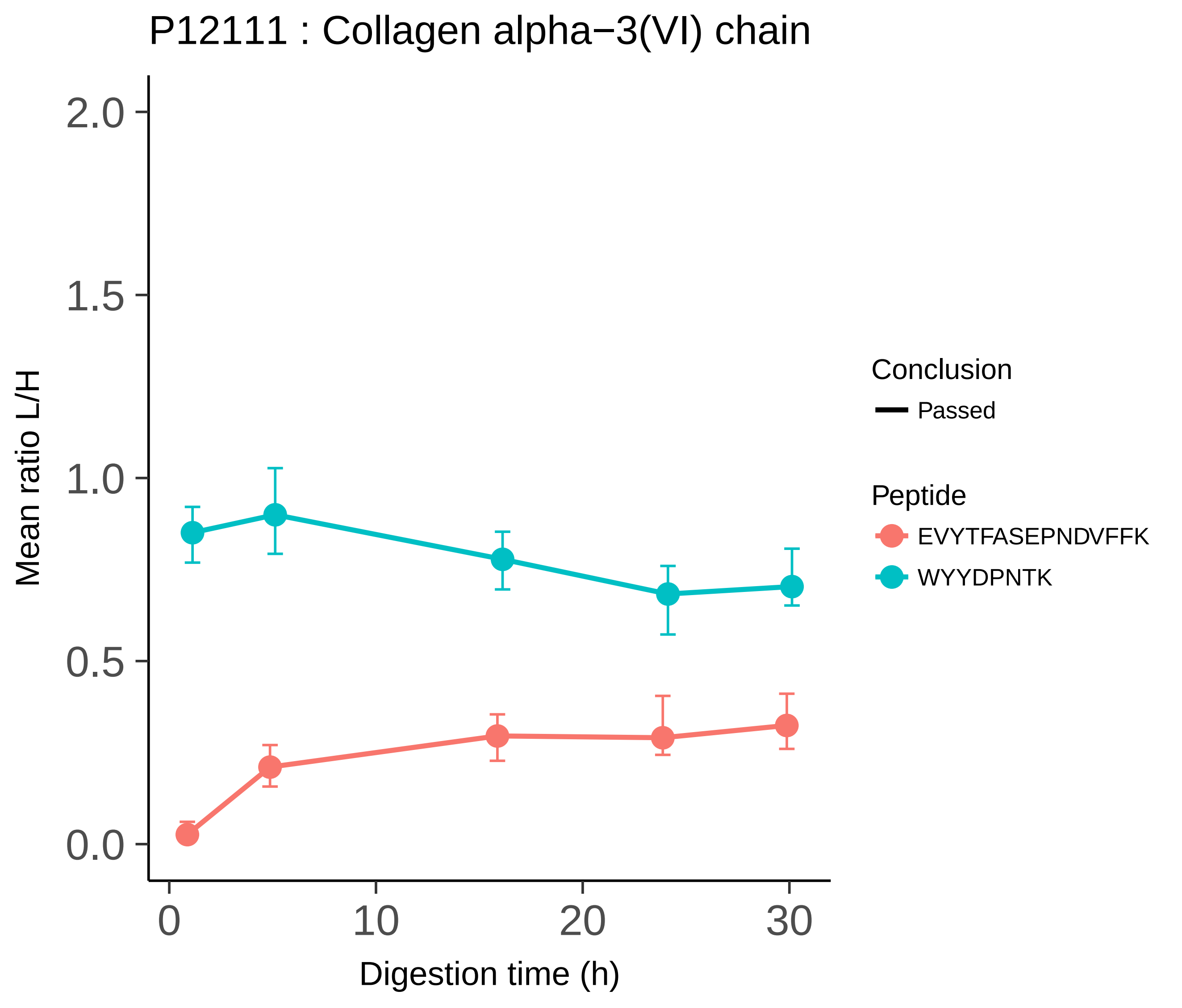
P12111 : Collagen alpha−3(VI) chain
Mean ratio L/H
2.0
1.5
1.0
0.5
0.0
0
10
20
30
Digestion time (h)
Conclusion
Passed
Peptide
EVYTFASEPNDVFFK
WYYDPNTK

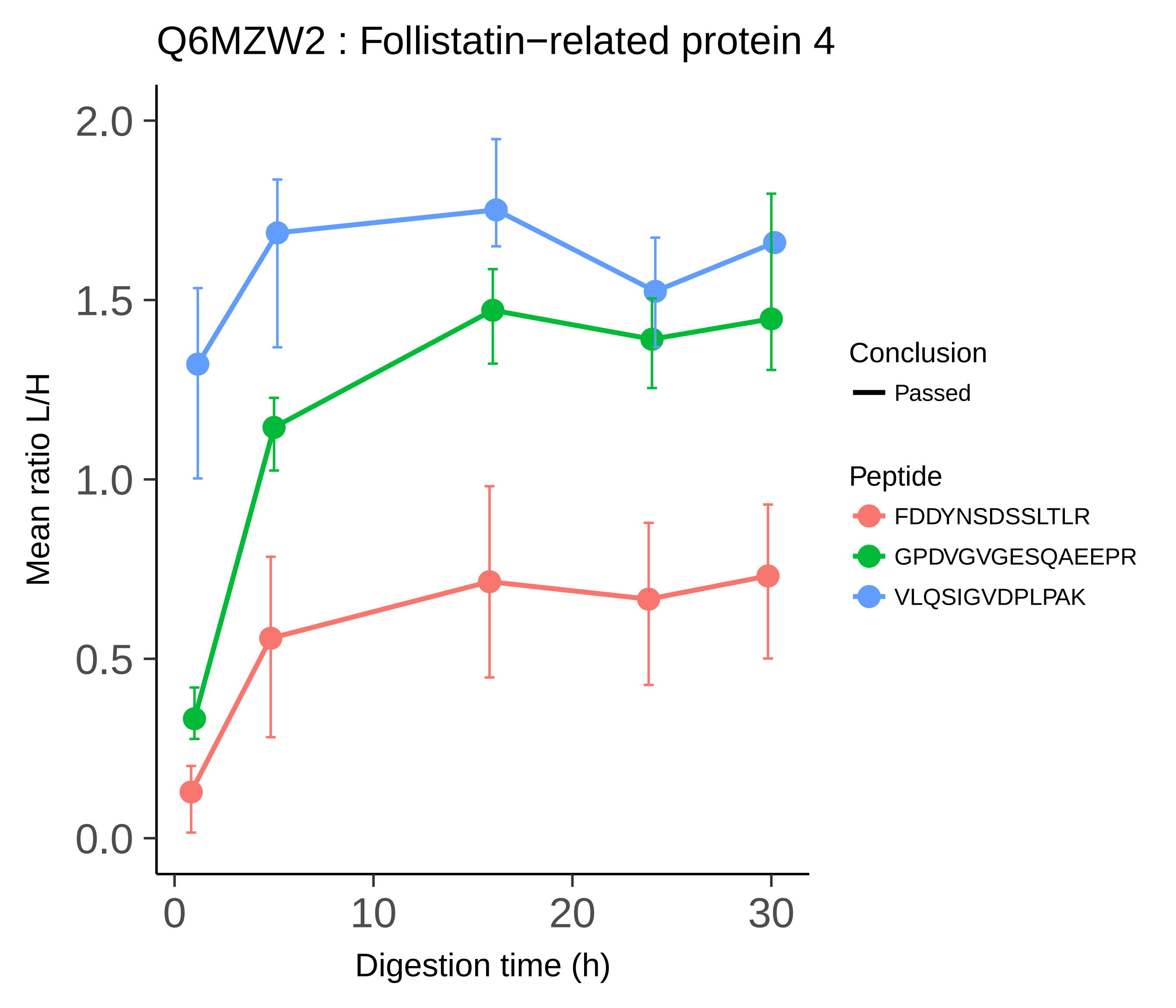
Q6MZW2 : Follistatin−related protein 4
Mean ratio L/H
2.0
1.5
1.0
0.5
0.0
0
10
20
30
Digestion time (h)
Conclusion
Passed
Peptide
FDDYNSDSSLTLR
GPDVGVGESQAEEPR
VLQSIGVDPLPAK

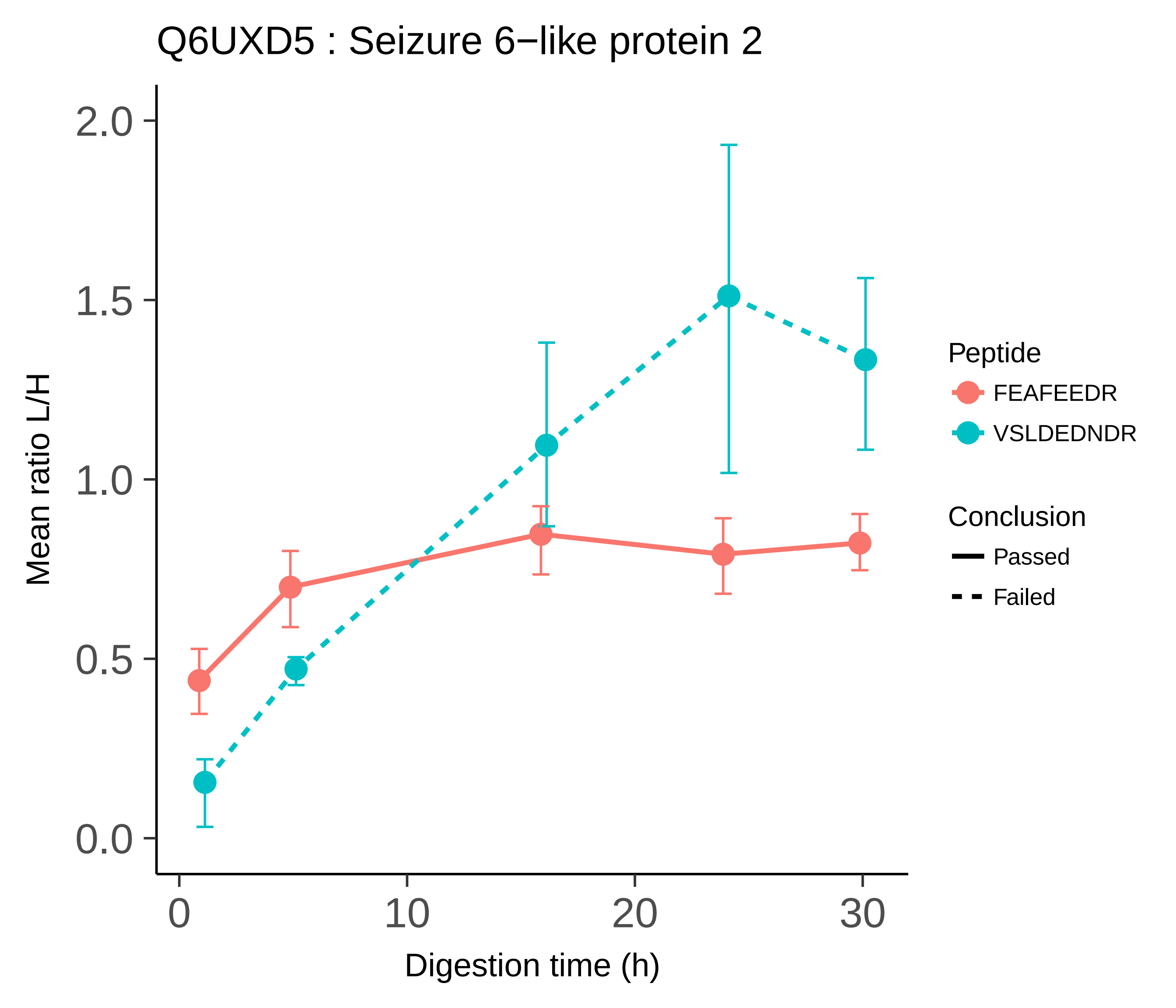
Q6UXD5 : Seizure 6−like protein 2
2.0
1.5
1.0
0.5
0.0
Mean ratio L/H
0
10
20
30
Digestion time (h)
Peptide
FEAFEEDR
VSLDEDNDR
Conclusion
Passed
Failed

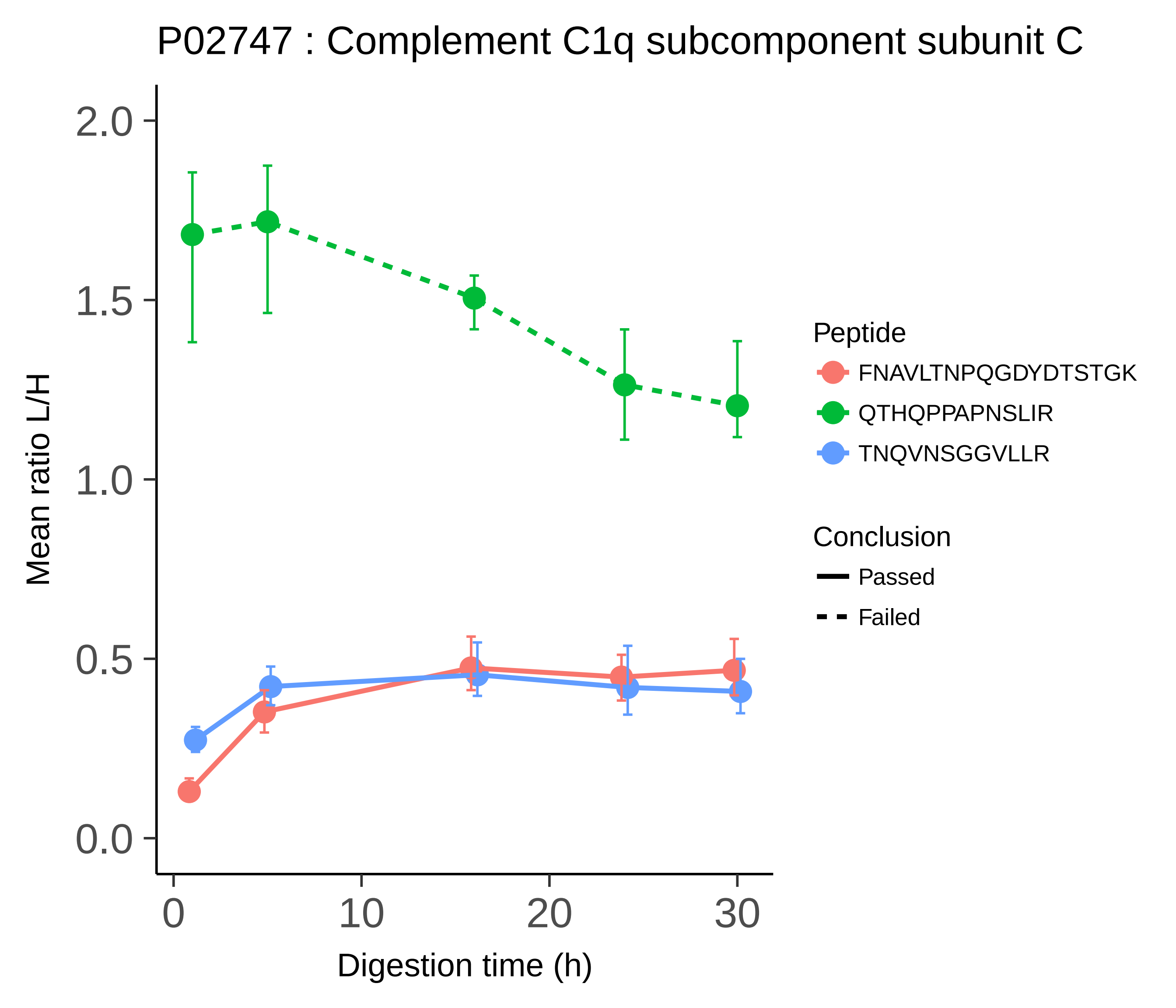
P02747 : Complement C1q subcomponent subunit C
Mean ratio L/H
2.0
1.5
1.0
0.5
0.0
0
10
20
30
Digestion time (h)
Peptide
FNAVLTNPQGDYDTSTGK
QTHQPPAPNSLIR
TNQVNSGGVLLR
Conclusion
Passed
Failed

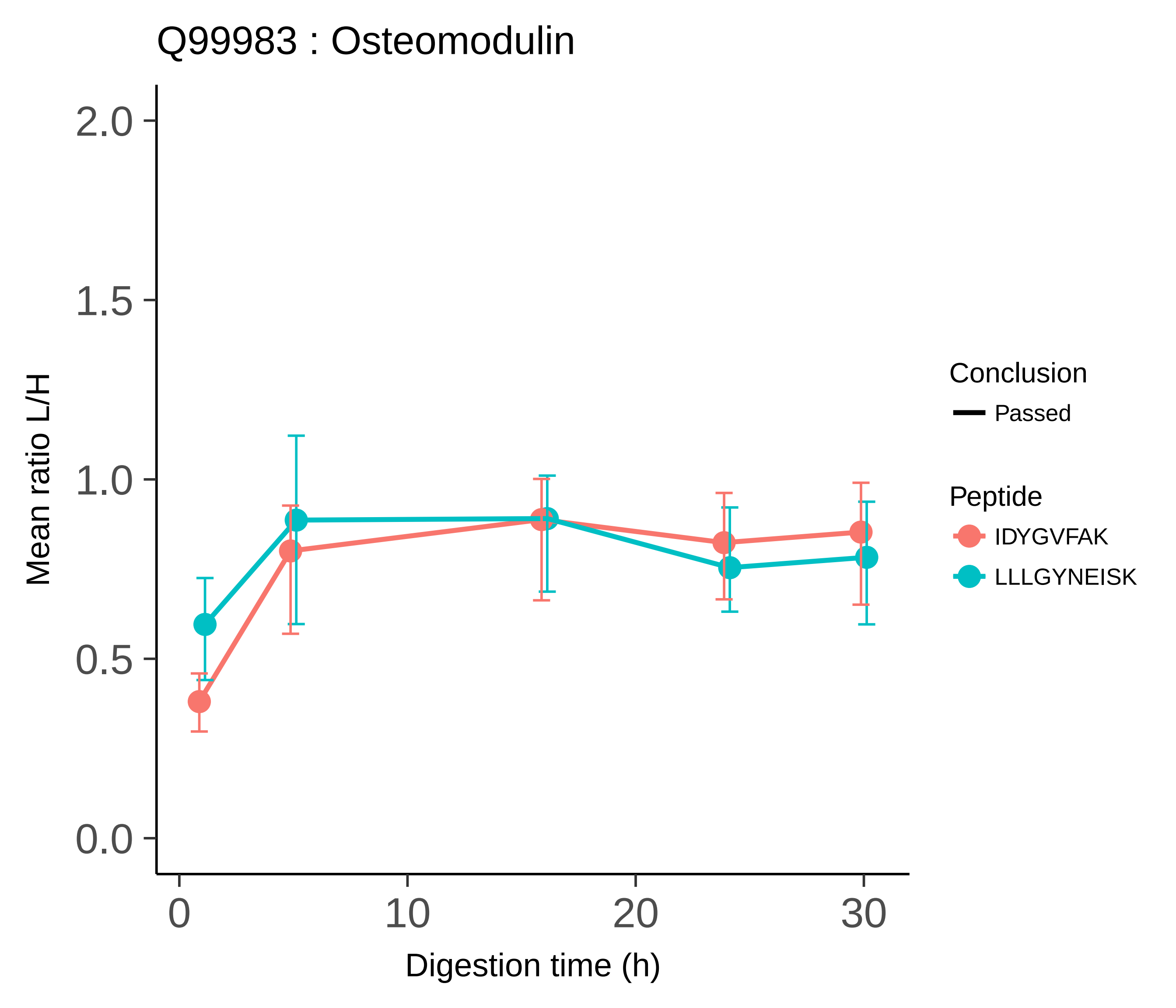
Q99983 : Osteomodulin
Mean ratio L/H
2.0
1.5
1.0
0.5
0.0
0
10
20
30
Digestion time (h)
Conclusion
Passed
Peptide
IDYGVFAK
LLLGYNEISK

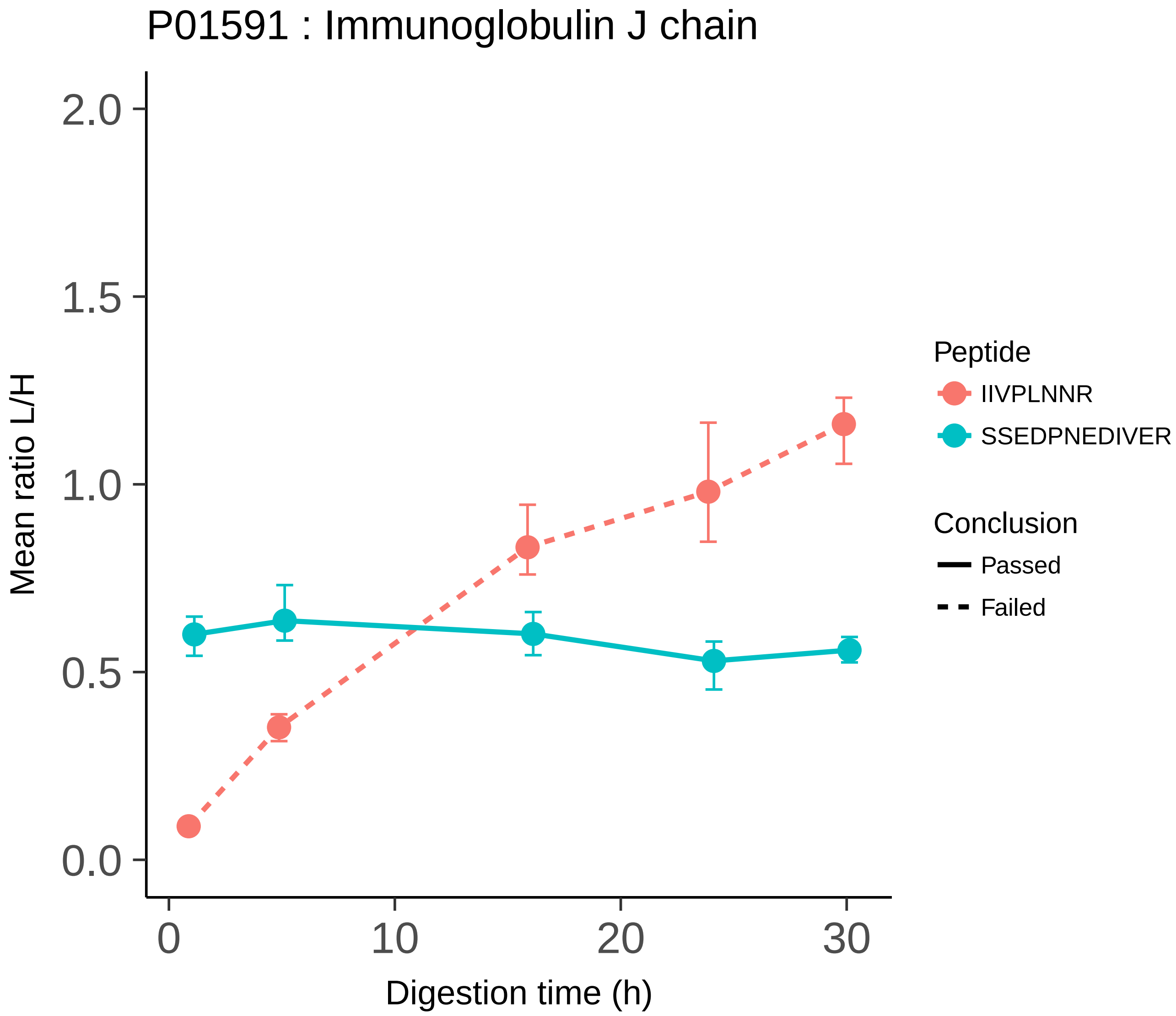
P01591 : Immunoglobulin J chain
Mean ratio L/H
2.0
1.5
1.0
0.5
0.0
0
10
20
30
Digestion time (h)
Peptide
IIVPLNNR
SSEDPNEDIVER
Conclusion
Passed
Failed

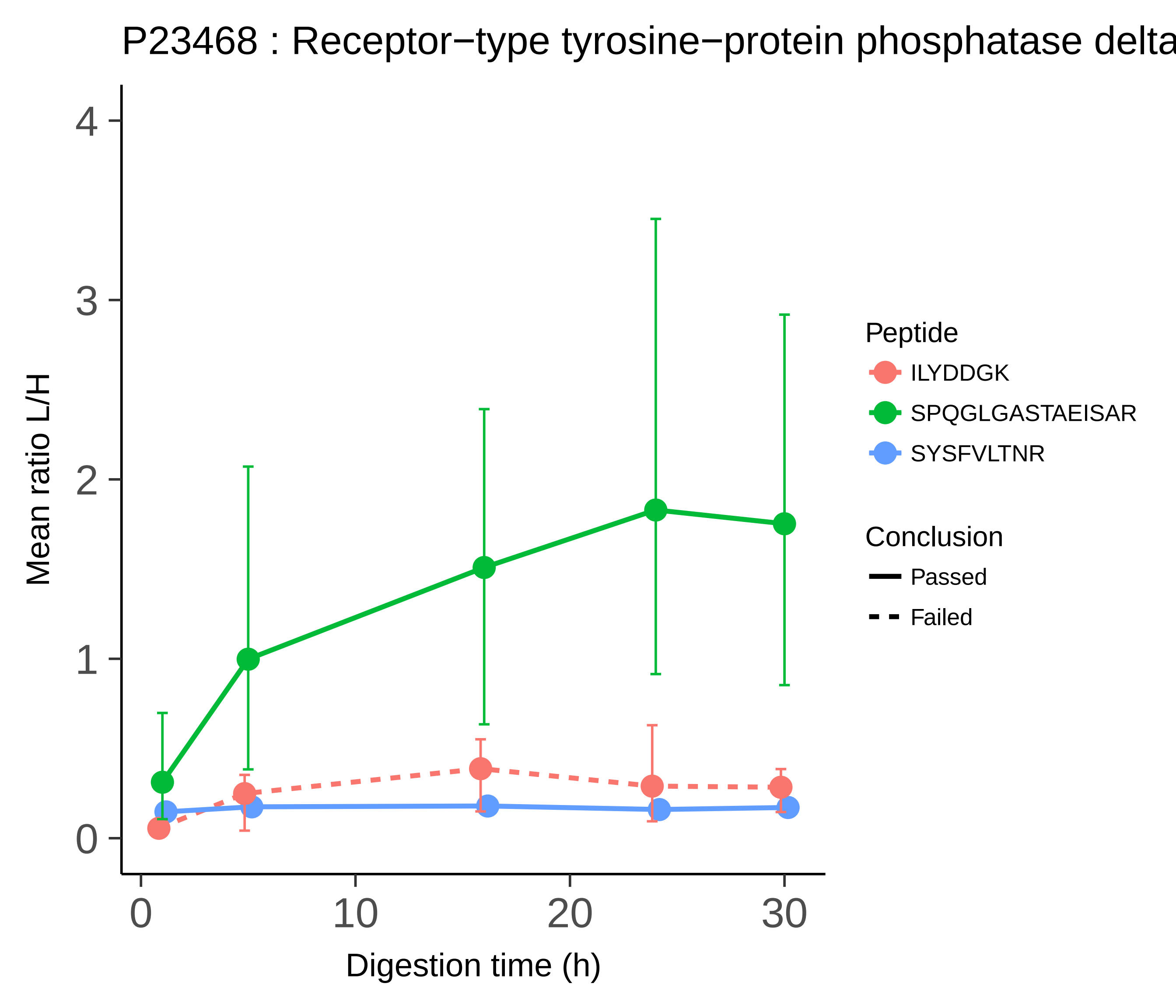

P23468 : Receptor−type tyrosine−protein phosphatase delta
Mean ratio L/H
4
3
2
1
0
0
10
20
30
Digestion time (h)
Peptide
ILYDDGK
SPQGLGASTAEISAR
SYSFVLTNR
Conclusion
Passed
Failed

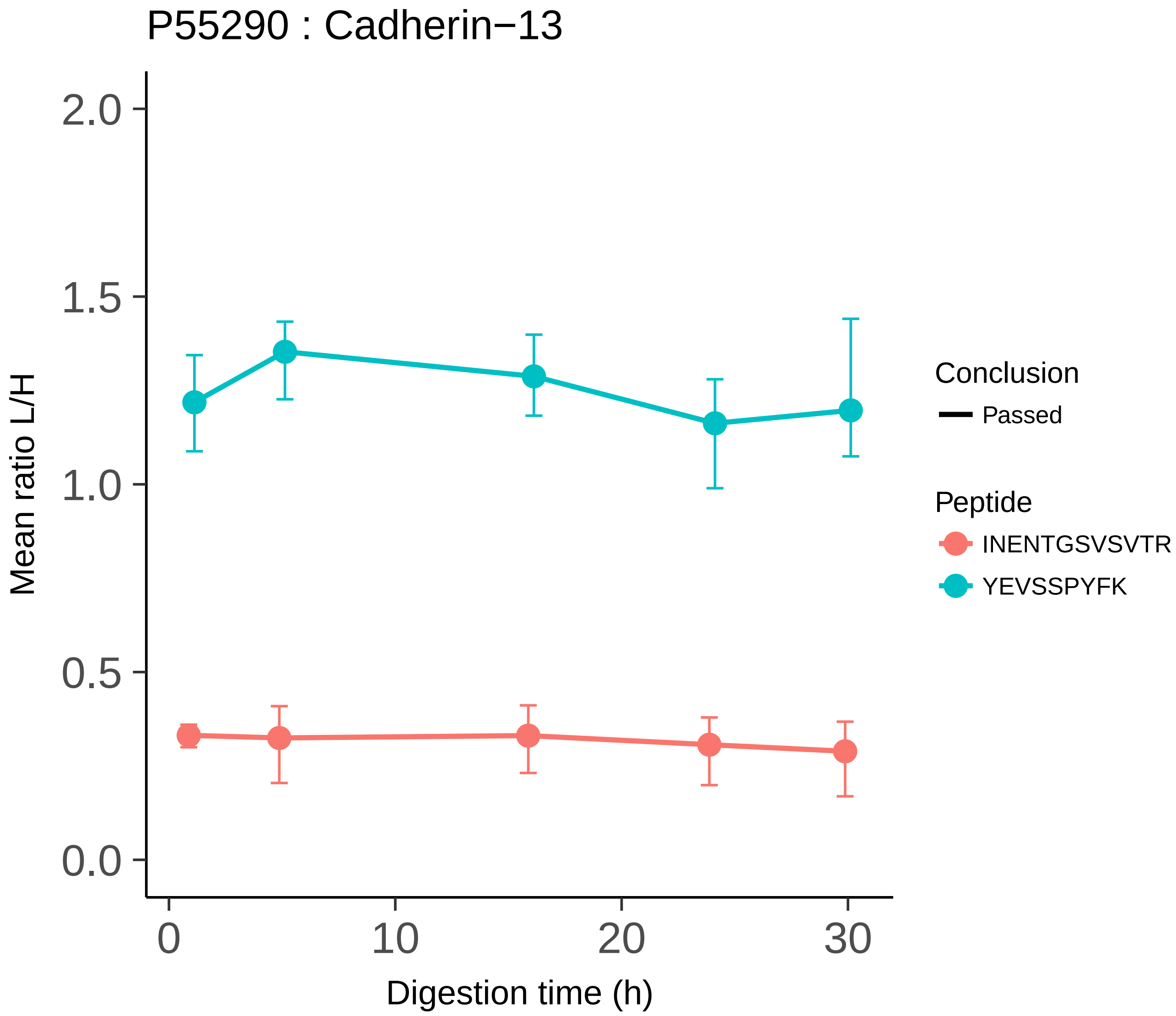

P55290 : Cadherin−13
Mean ratio L/H
2.0
1.5
1.0
0.5
0.0
0
10
20
30
Digestion time (h)
Conclusion
Passed
Peptide
INENTGSVSVTR
YEVSSPYFK

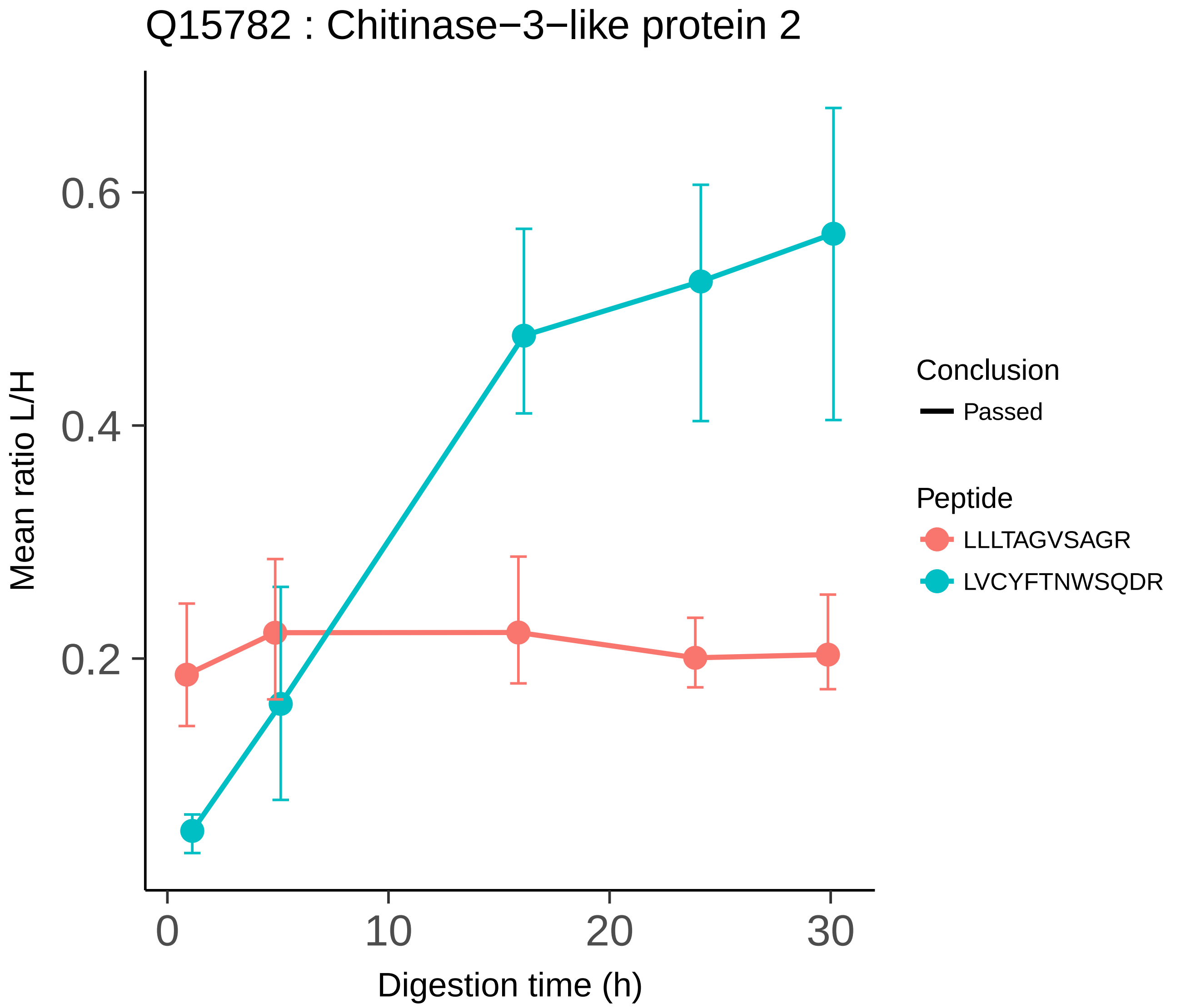
Q15782 : Chitinase−3−like protein 2
Mean ratio L/H
0.6
0.4
0.2
0
10
20
30
Digestion time (h)
Conclusion
Passed
Peptide
LLLTAGVSAGR
LVCYFTNWSQDR

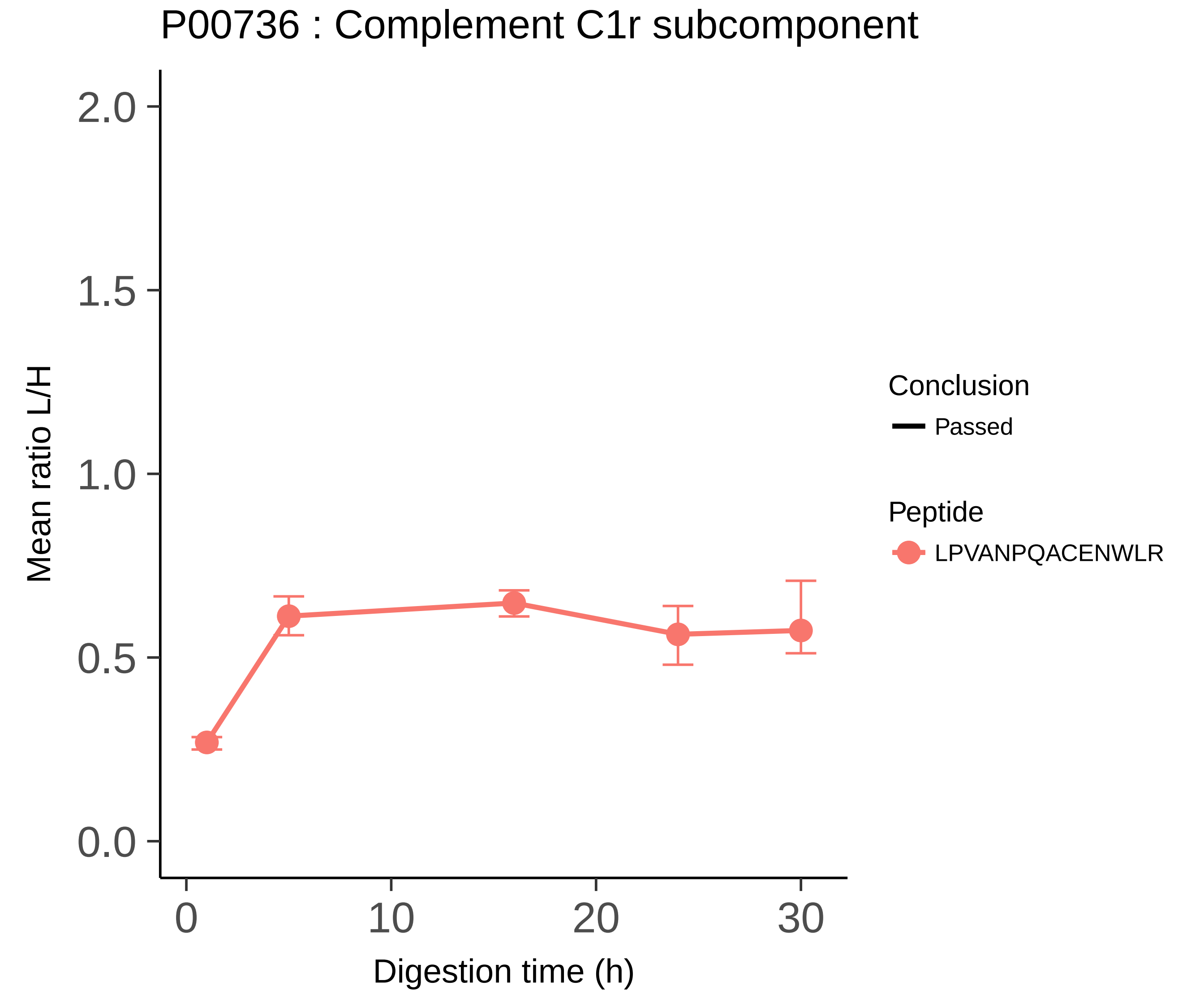
P00736 : Complement C1r subcomponent
Mean ratio L/H
2.0
1.5
1.0
0.5
0.0
0
10
20
30
Digestion time (h)
Conclusion
Passed
Peptide
LPVANPQACENWLR

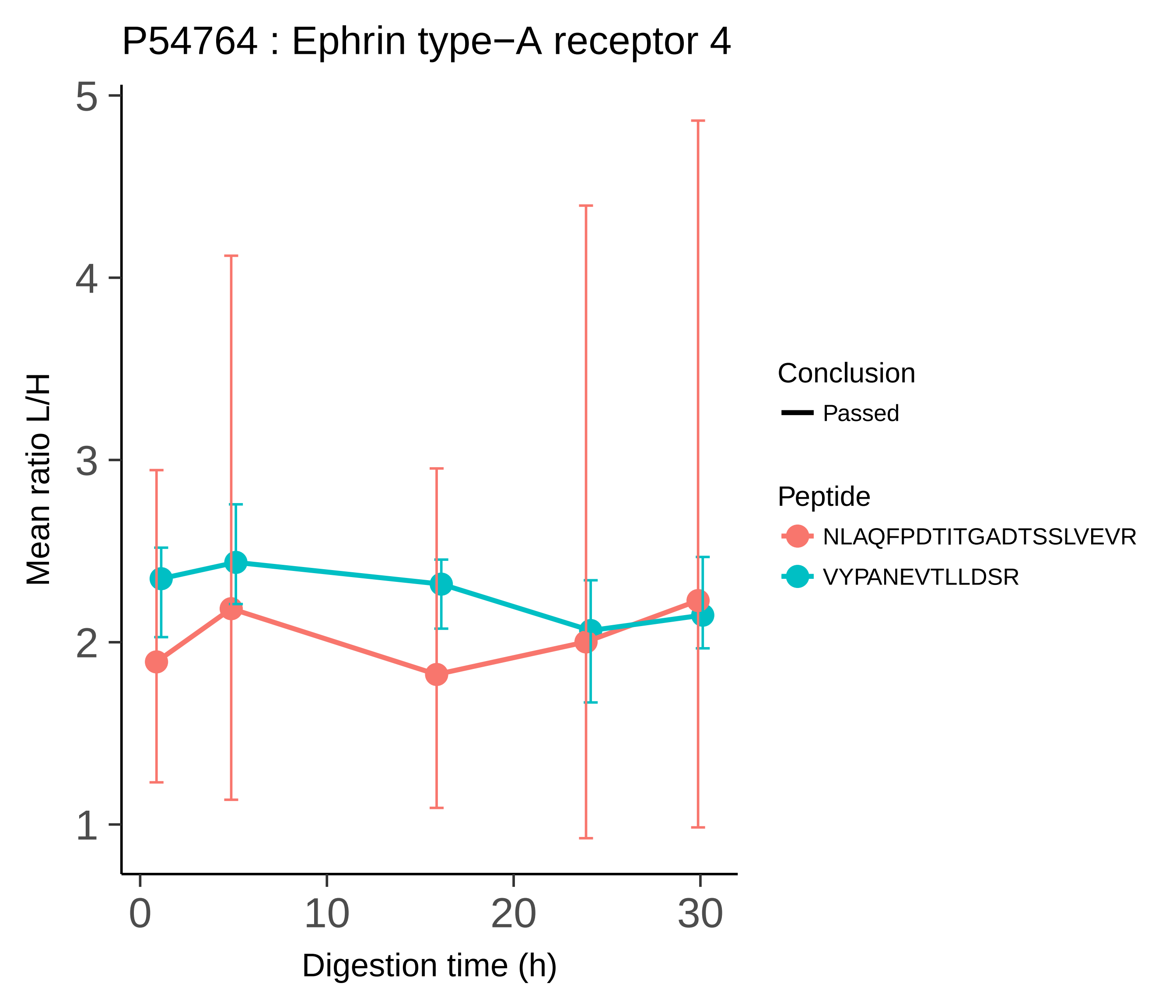
P54764 : Ephrin type−A receptor 4
Mean ratio L/H
1
2
3
4
5
0
10
20
30
Digestion time (h)
Conclusion
Passed
Peptide
NLAQFPDTITGADTSSLVEVR
VYPANEVTLLLDSR

P48058 : Glutamate receptor 4

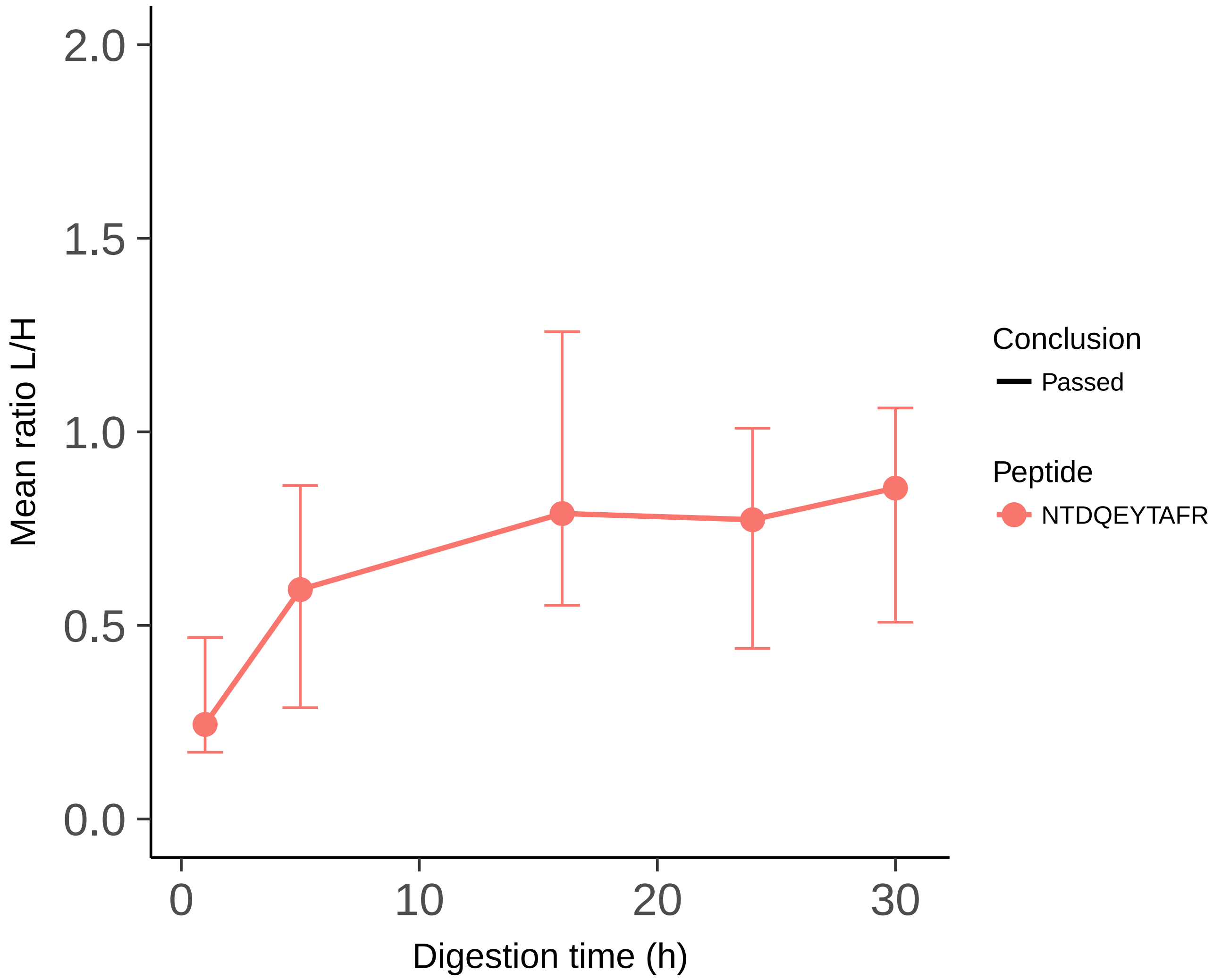

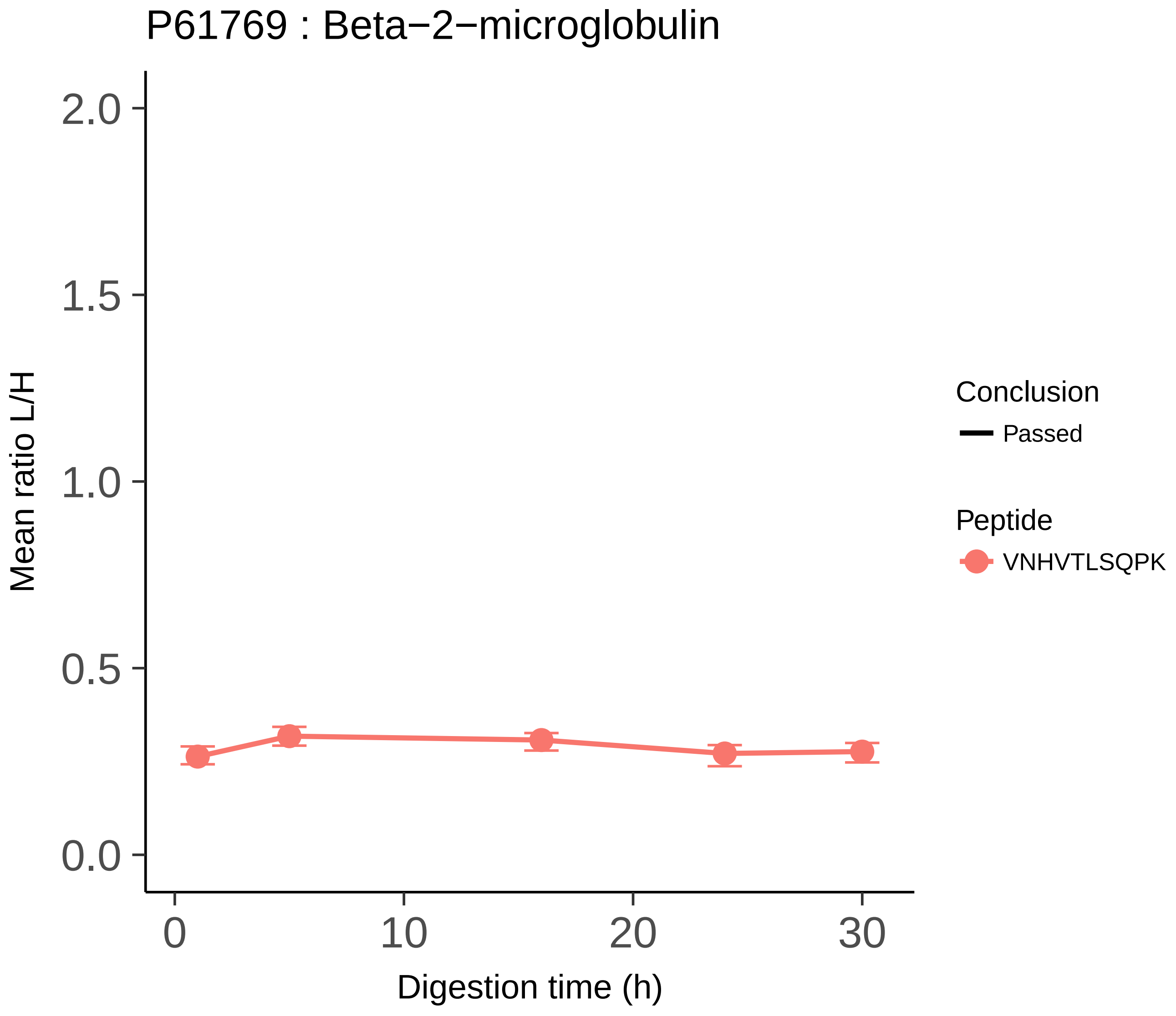
P61769 : Beta−2−microglobulin
2.0
1.5
1.0
0.5
0.0
Mean ratio L/H
0
10
20
30
Digestion time (h)
Conclusion
Passed
Peptide
VNHVTLSQPK
