# Supplementary figures and images for: Development of robust targeted proteomics assays for cerebrospinal fluid biomarkers in multiple sclerosis
Source: Clin Proteomics. 2020 Sep 18;17:33. doi: 10.1186/s12014-020-09296-5 (PMC7499868; doi:10.1186/s12014-020-09296-5)

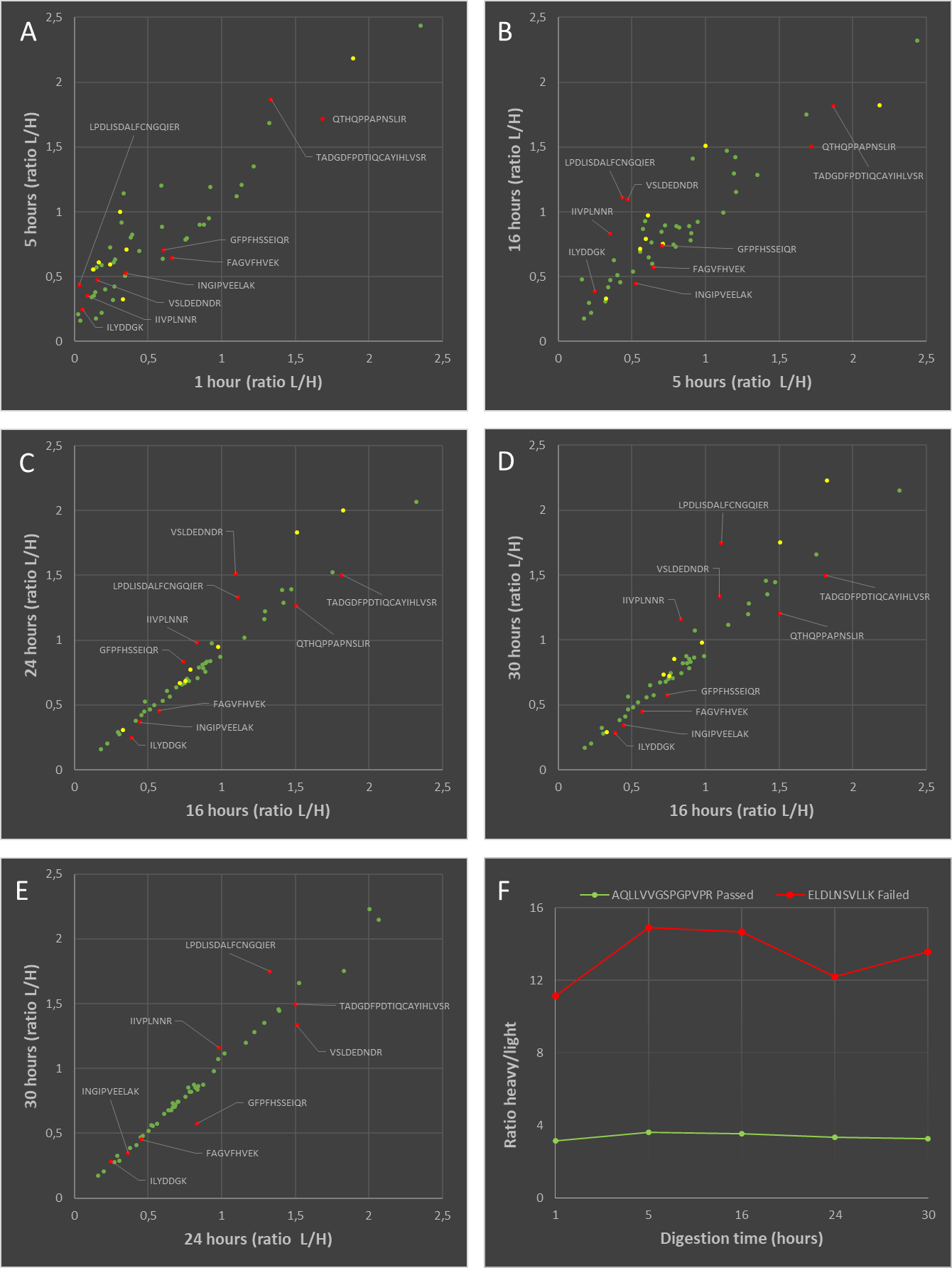

Supplement: Supplementary file 8 — Additional file 8: Fig. S2. Scatter plots comparing peptide amount (ratio L/H) at all times points tested in the digestion experiment. [file 12014_2020_9296_MOESM8_ESM.png]
