# Supplementary material for: Development of robust targeted proteomics assays for cerebrospinal fluid biomarkers in multiple sclerosis
Source: Clin Proteomics. 2020 Sep 18;17:33. doi: 10.1186/s12014-020-09296-5 (PMC7499868; doi:10.1186/s12014-020-09296-5)
Supplement: Supplementary file 11 — Additional file 11: Table S9. Overview of the tests for stability, digestion and MS vs. OND experiments, for the 25 proteins. The colours and numbers refer to the peptides passing the specific test. Green: protein passed with two or more peptides; yellow: passed with one peptide; and, red: either no peptides passed (0) or none were tested (-). [file 12014_2020_9296_MOESM11_ESM.pdf]

Accession	Protein Name	Stability	Digestion	MS vs. OND
P51693	Amyloid-like protein 1	3	2	2
P61769	Beta-2-microglobulin	2	1	1
P55290	Cadherin-13	3	2	2
P16070	CD44 antigen	3	2	1
P36222	Chitinase-3-like protein 1	0	-	-
Q15782	Chitinase-3-like protein 2	2	2	2
P10645	Chromogranin-A	3	3	3
P12111	Collagen alpha-3(VI) chain	3	3	1
P02747	Complement C1q subcomponent subunit C	3	2	0
P00736	Complement C1r subcomponent	4	1	0
P54764	Ephrin type-A receptor 4	3	2	2
Q6MZW2	Follistatin-related protein 4	3	3	3
P48058	Glutamate receptor 4	3	1	1
P01591	Immunoglobulin J chain	2	1	1
Q92876	Kallikrein-6	3	1	1
P32004	Neural cell adhesion molecule L1	3	2	2
Q9ULB1	Neurexin-1	3	2	2
Q9P2S2	Neurexin-2	3	3	3
Q92823	Neuronal cell adhesion molecule	3	3	3
Q99983	Osteomodulin	2	2	0
Q9UHG2	ProSAAS	1	1	1
P23468	Receptor-type tyrosine-protein phosphatase delta	3	2	2
O00584	Ribonuclease T2	2	1	1
P13521	Secretogranin-2	3	2	2
Q6UXD5	Seizure 6-like protein 2	2	1	1

Supplementary Table 9: Overview of the tests for stability, digestion and MS vs. OND experiments, for the 25 proteins. The colours and numbers refer to the peptides passing the specific test. Green: protein passed with two or more peptides; yellow: passed with one peptide; and, red: either no peptides passed (0) or none were tested (-).
