# Supplementary material for: Development of robust targeted proteomics assays for cerebrospinal fluid biomarkers in multiple sclerosis
Source: Clin Proteomics. 2020 Sep 18;17:33. doi: 10.1186/s12014-020-09296-5 (PMC7499868; doi:10.1186/s12014-020-09296-5)
Supplement: Supplementary file 12 — Additional file 12: Fig. S3. Calibration curves for 17 assay peptides that passed all quality control tests. [file 12014_2020_9296_MOESM12_ESM.pdf]

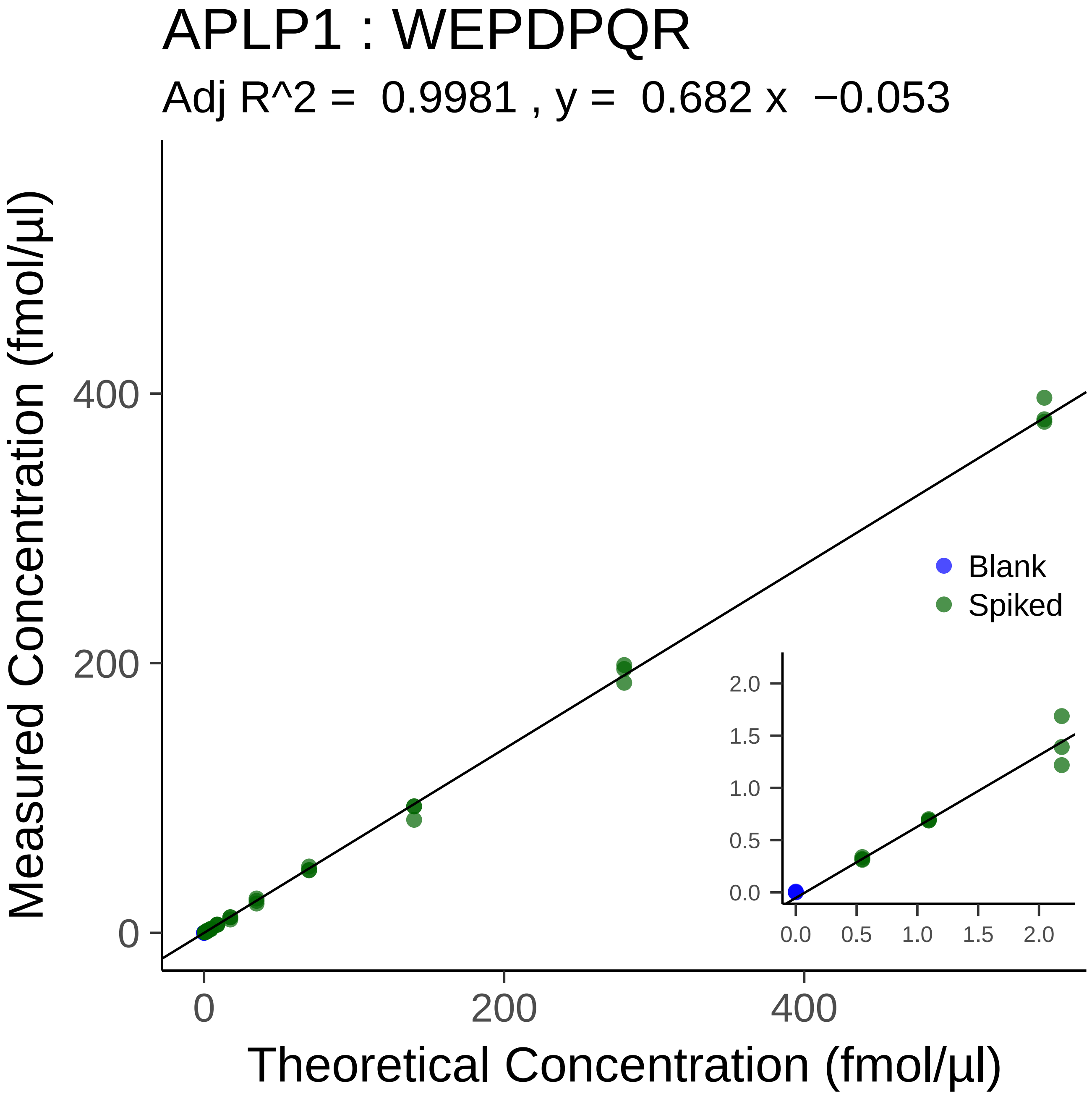

APLP1 : WEPDPQR
Adj R^2 = 0.9981 , y = 0.682 x −0.053
Measured Concentration (fmol/µl)
Theoretical Concentration (fmol/µl)
0
200
400
Blank
Spiked
0.0
0.5
1.0
1.5
2.0

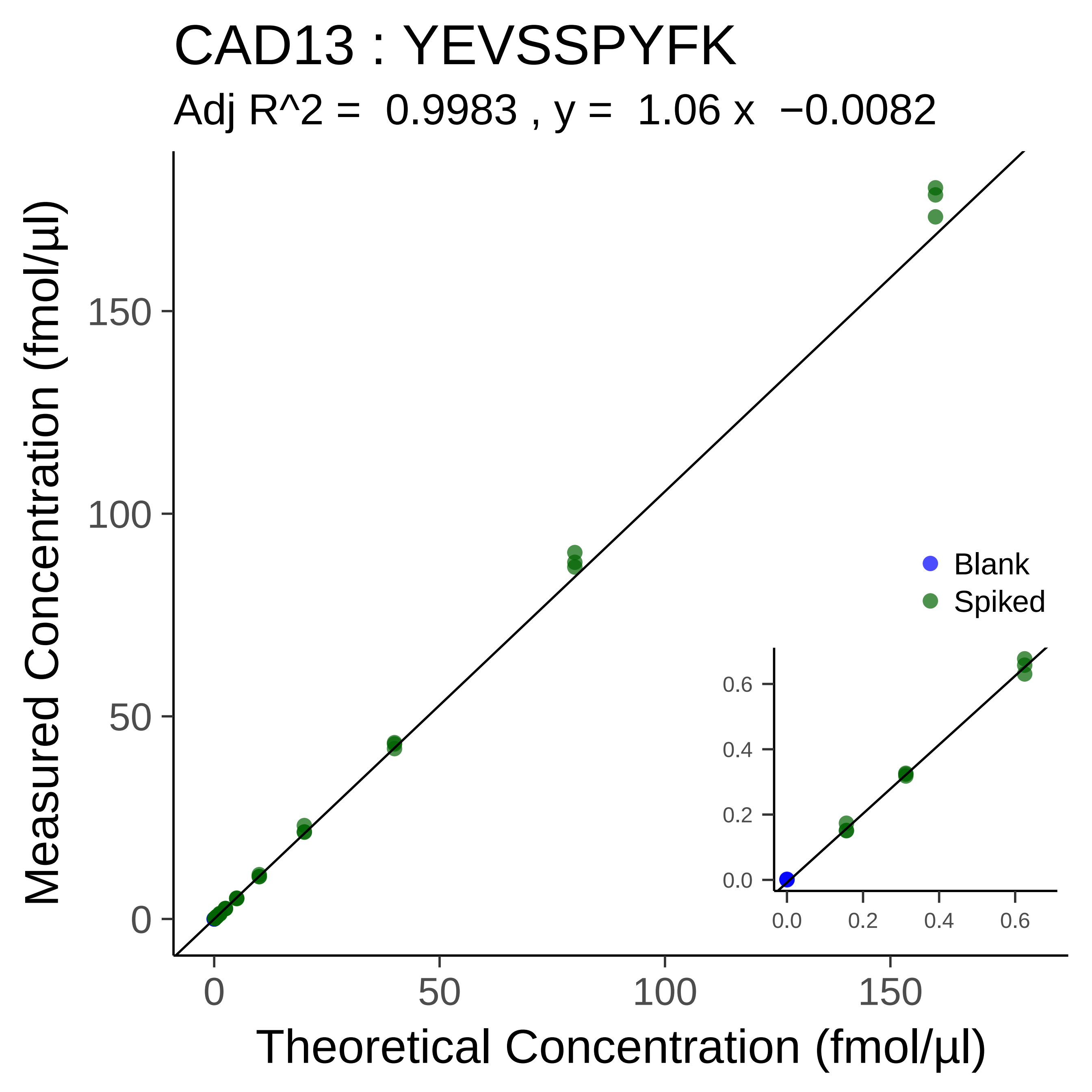

CAD13 : YEVSSPYFK
Adj R^2 = 0.9983 , y = 1.06 x −0.0082
Measured Concentration (fmol/µl)
Theoretical Concentration (fmol/µl)
0
50
100
150
0
50
100
150
Blank
Spiked
0.0
0.2
0.4
0.6
0.0
0.2
0.4
0.6

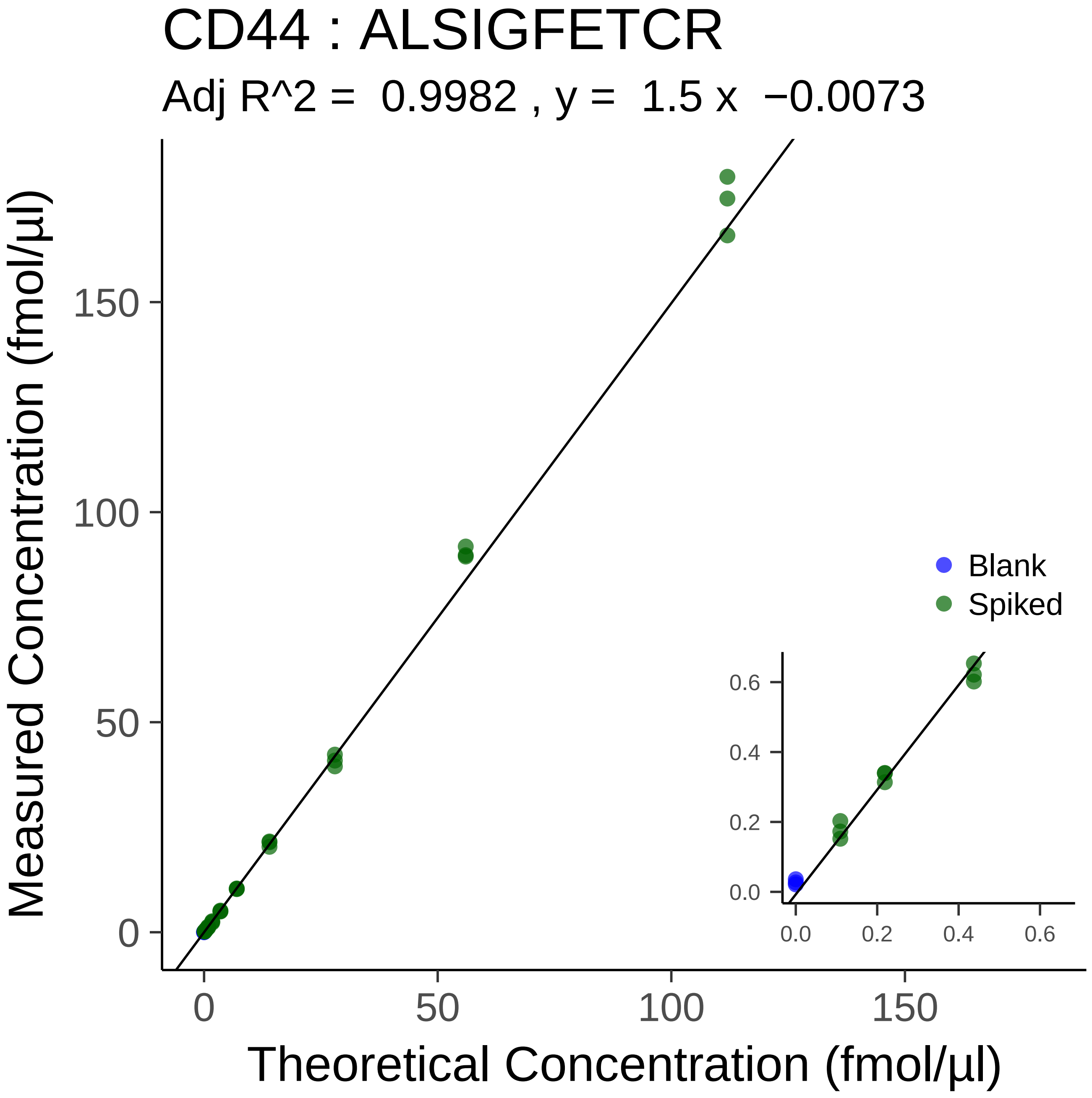

CD44 : ALSIGFETCR
Adj R^2 = 0.9982 , y = 1.5 x −0.0073
Measured Concentration (fmol/µl)
Theoretical Concentration (fmol/µl)
0
50
100
150
Blank
Spiked
0.0
0.2
0.4
0.6

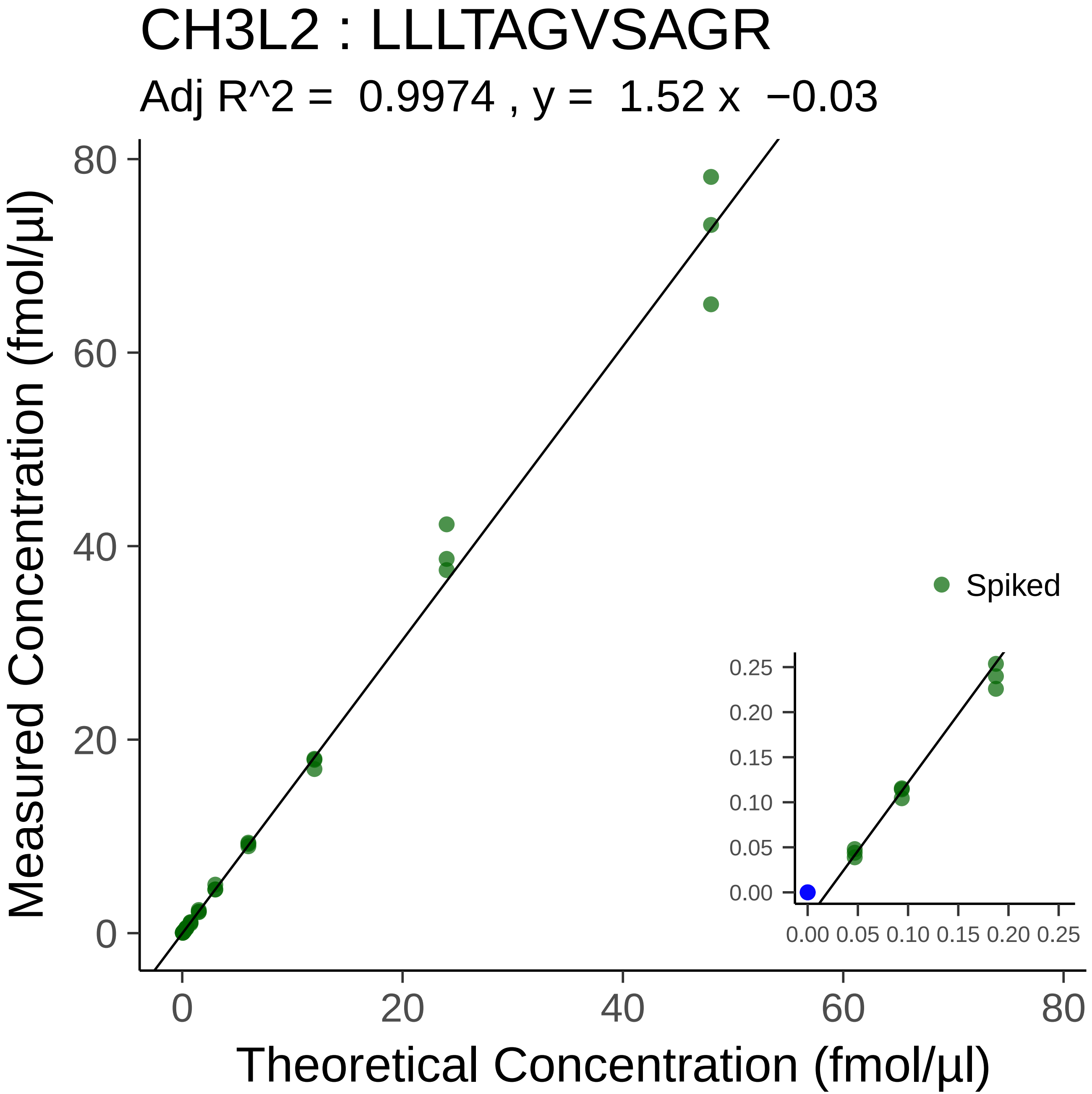

CH3L2 : LLLTAGVSAGR
Adj R^2 = 0.9974 , y = 1.52 x −0.03
Measured Concentration (fmol/µl)
Theoretical Concentration (fmol/µl)
0
20
40
60
80
Spiked
0.00
0.05
0.10
0.15
0.20
0.25

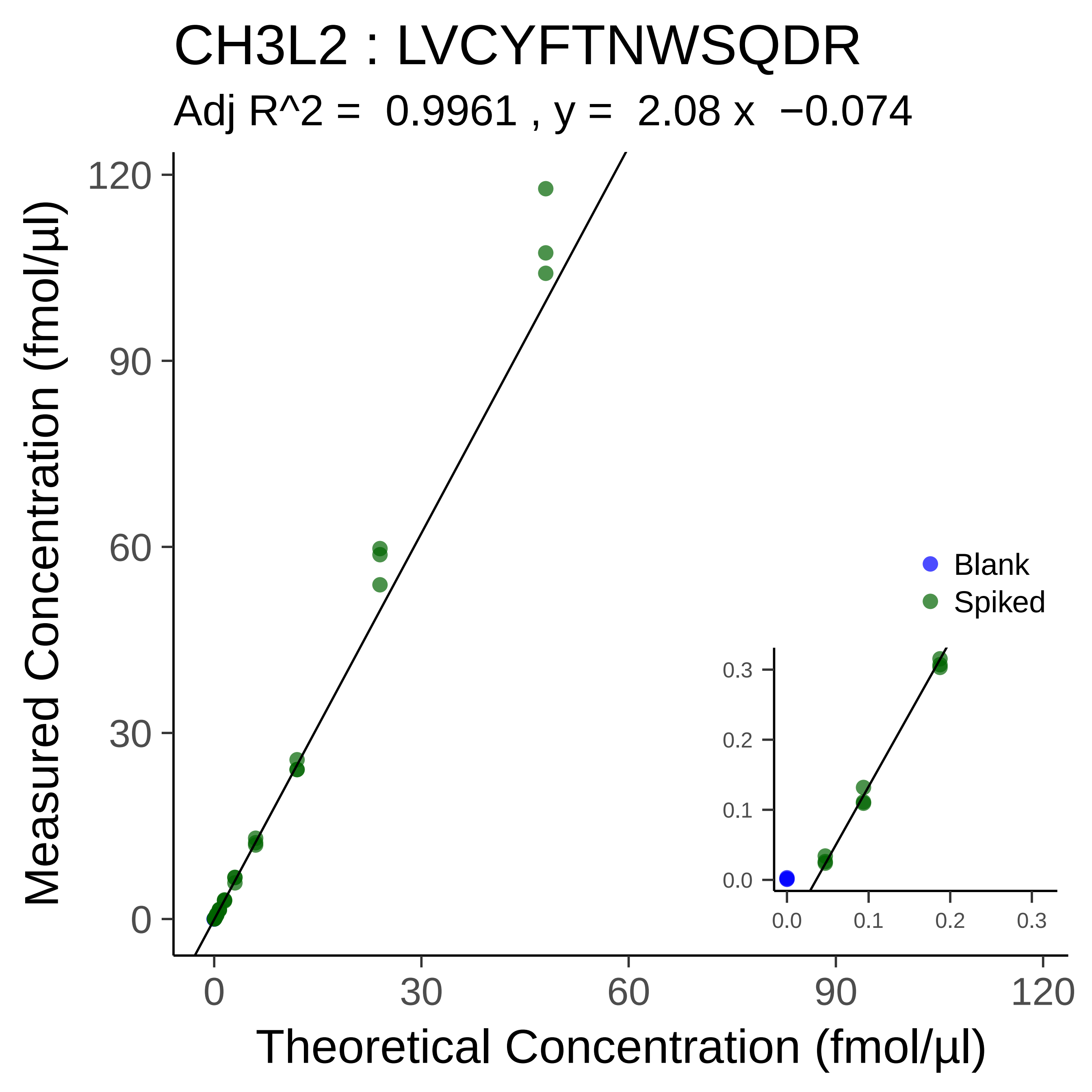

CH3L2 : LVCYFTNWSQDR
Adj R^2 = 0.9961 , y = 2.08 x −0.074
Measured Concentration (fmol/µl)
Theoretical Concentration (fmol/µl)
0
30
60
90
120
Blank
Spiked
0.0
0.1
0.2
0.3

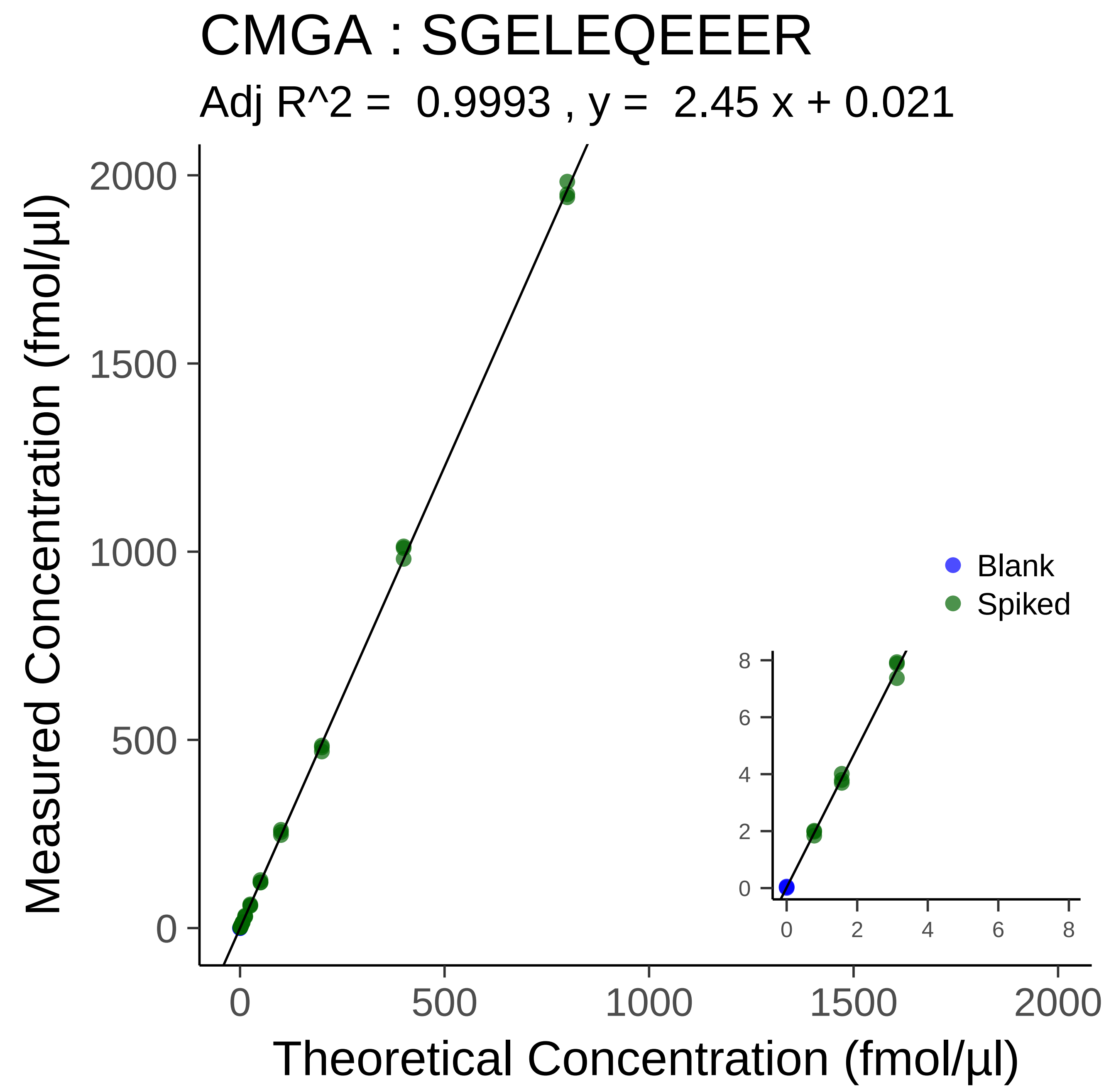

CMGA : SGELEQEEER
Adj R^2 = 0.9993 , y = 2.45 x + 0.021
Measured Concentration (fmol/µl)
Theoretical Concentration (fmol/µl)
0
500
1000
1500
2000
Blank
Spiked
0
2
4
6
8

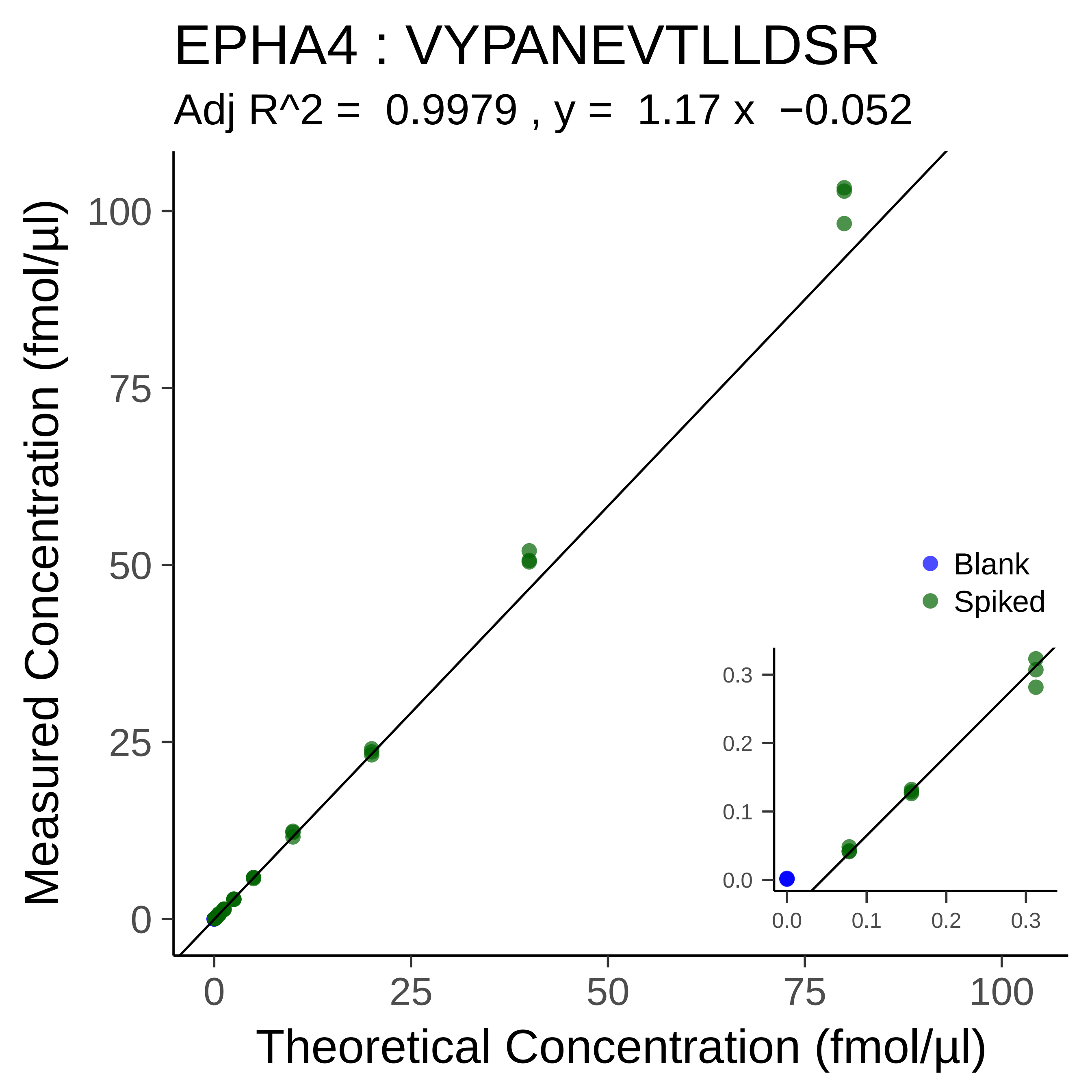

EPHA4 : VYPANEVTLLLDSR
Adj R^2 = 0.9979 , y = 1.17 x −0.052
Measured Concentration (fmol/µl)
Theoretical Concentration (fmol/µl)
0
25
50
75
100
Blank
Spiked
0.0
0.1
0.2
0.3

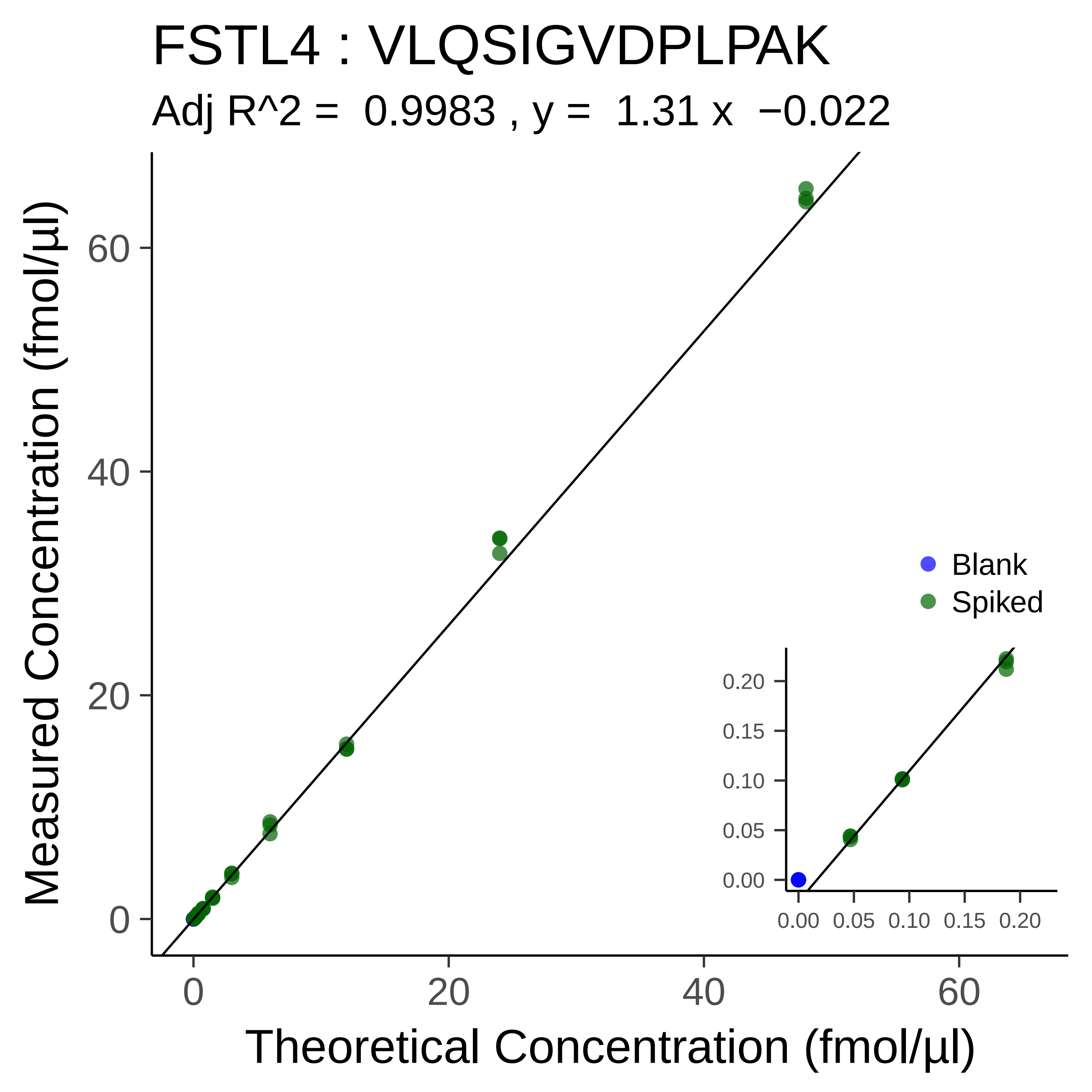
FSTL4 : VLQSIGVDPLPAK
Adj R^2 = 0.9983 , y = 1.31 x −0.022
Measured Concentration (fmol/µl)
Theoretical Concentration (fmol/µl)
0
20
40
60
Blank
Spiked
0.00
0.05
0.10
0.15
0.20

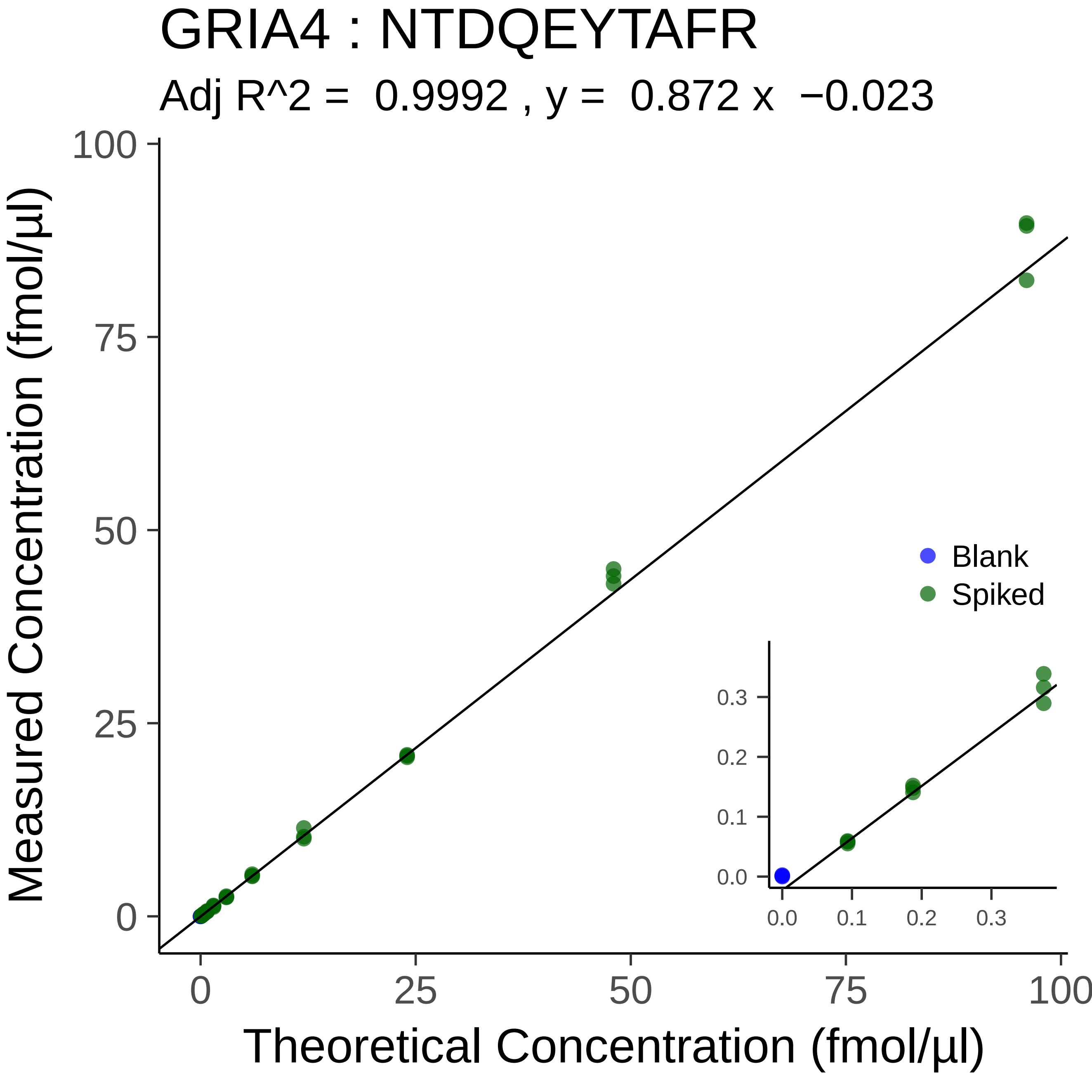

GRIA4 : NTDQEYTAFR
Adj R^2 = 0.9992 , y = 0.872 x −0.023
Measured Concentration (fmol/µl)
Theoretical Concentration (fmol/µl)
100
75
50
25
0
0
25
50
75
100
Blank
Spiked
0.3
0.2
0.1
0.0
0.0
0.1
0.2
0.3

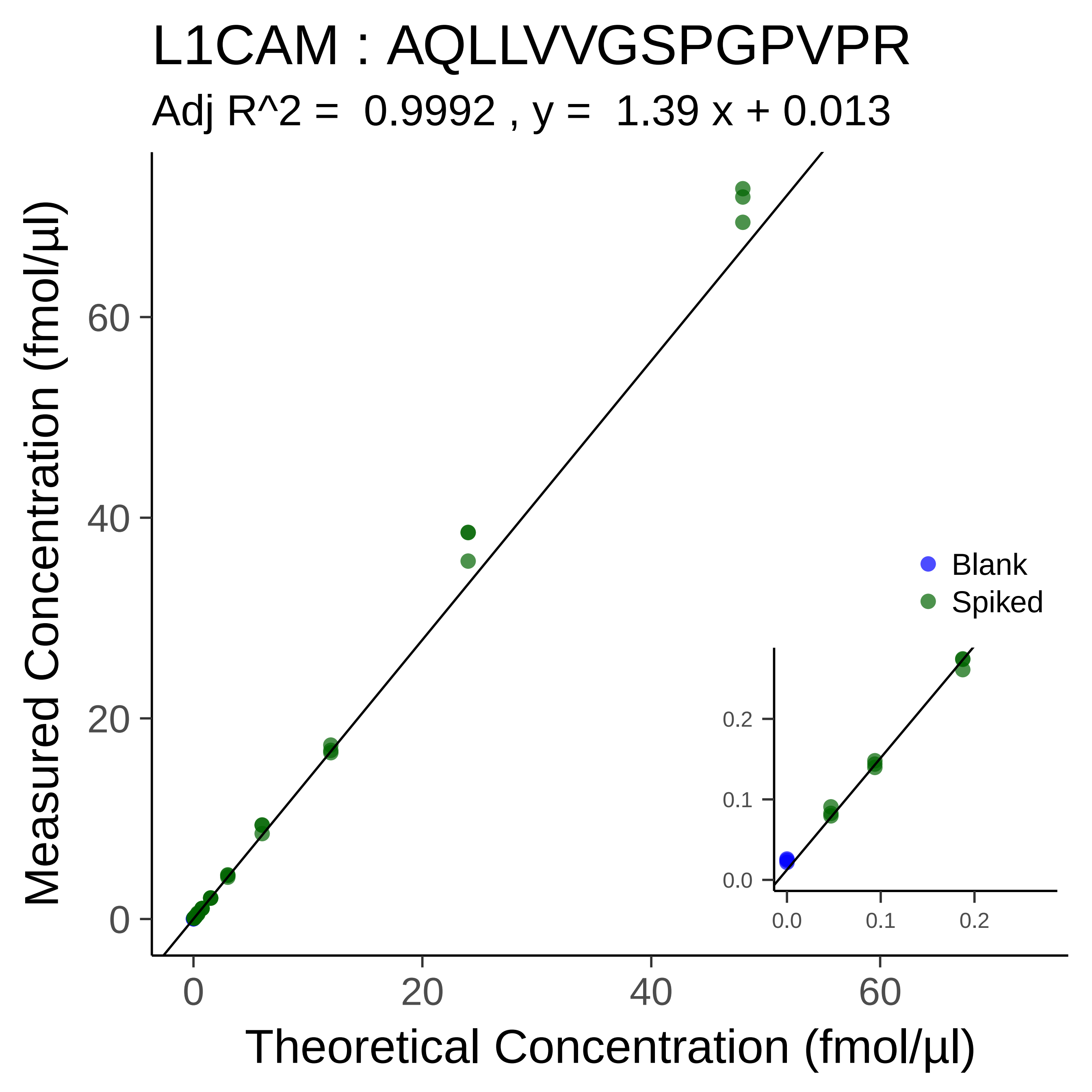
L1CAM : AQLLVVGSPGPVPR
Adj R^2 = 0.9992 , y = 1.39 x + 0.013
Measured Concentration (fmol/µl)
Theoretical Concentration (fmol/µl)
0
20
40
60
Blank
Spiked
0.0
0.1
0.2

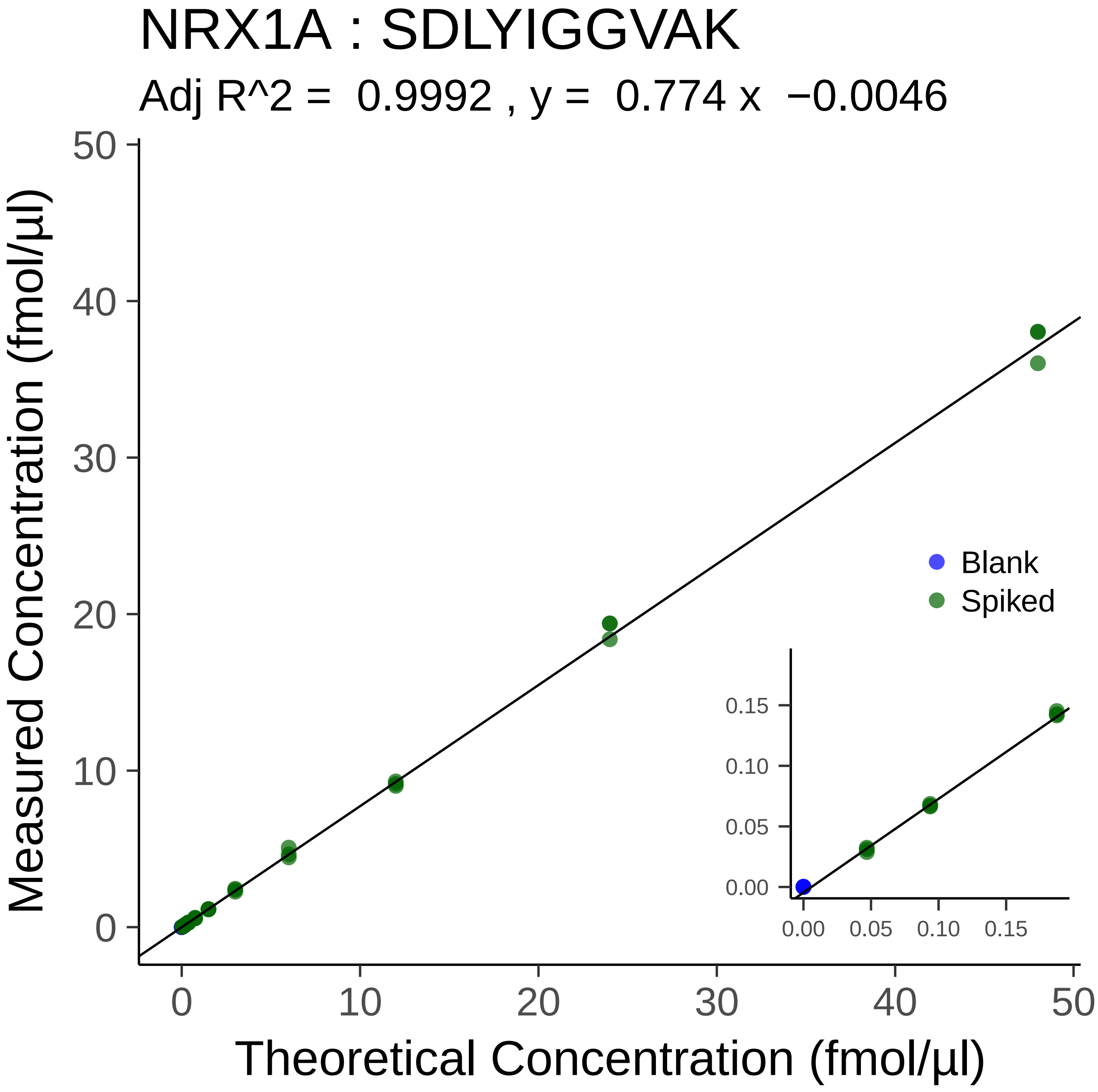
NRX1A : SDLYIGGVAK
Adj R^2 = 0.9992 , y = 0.774 x −0.0046
Measured Concentration (fmol/µl)
Theoretical Concentration (fmol/µl)
0
10
20
30
40
50
Blank
Spiked
0.00
0.05
0.10
0.15

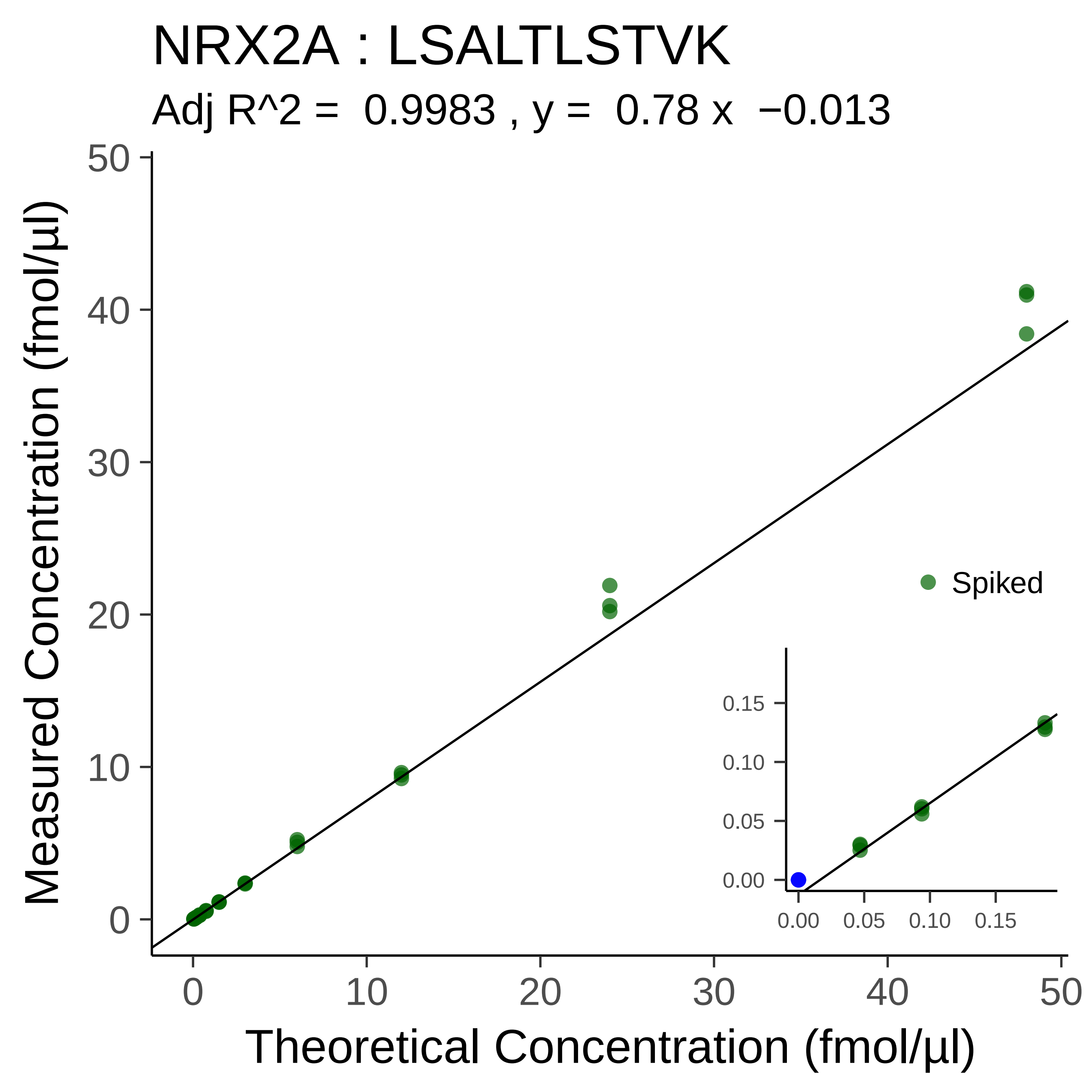

NRX2A : LSALTLSTVK
Adj R^2 = 0.9983 , y = 0.78 x −0.013
Measured Concentration (fmol/µl)
Theoretical Concentration (fmol/µl)
Spiked
0
10
20
30
40
50
0.00
0.05
0.10
0.15

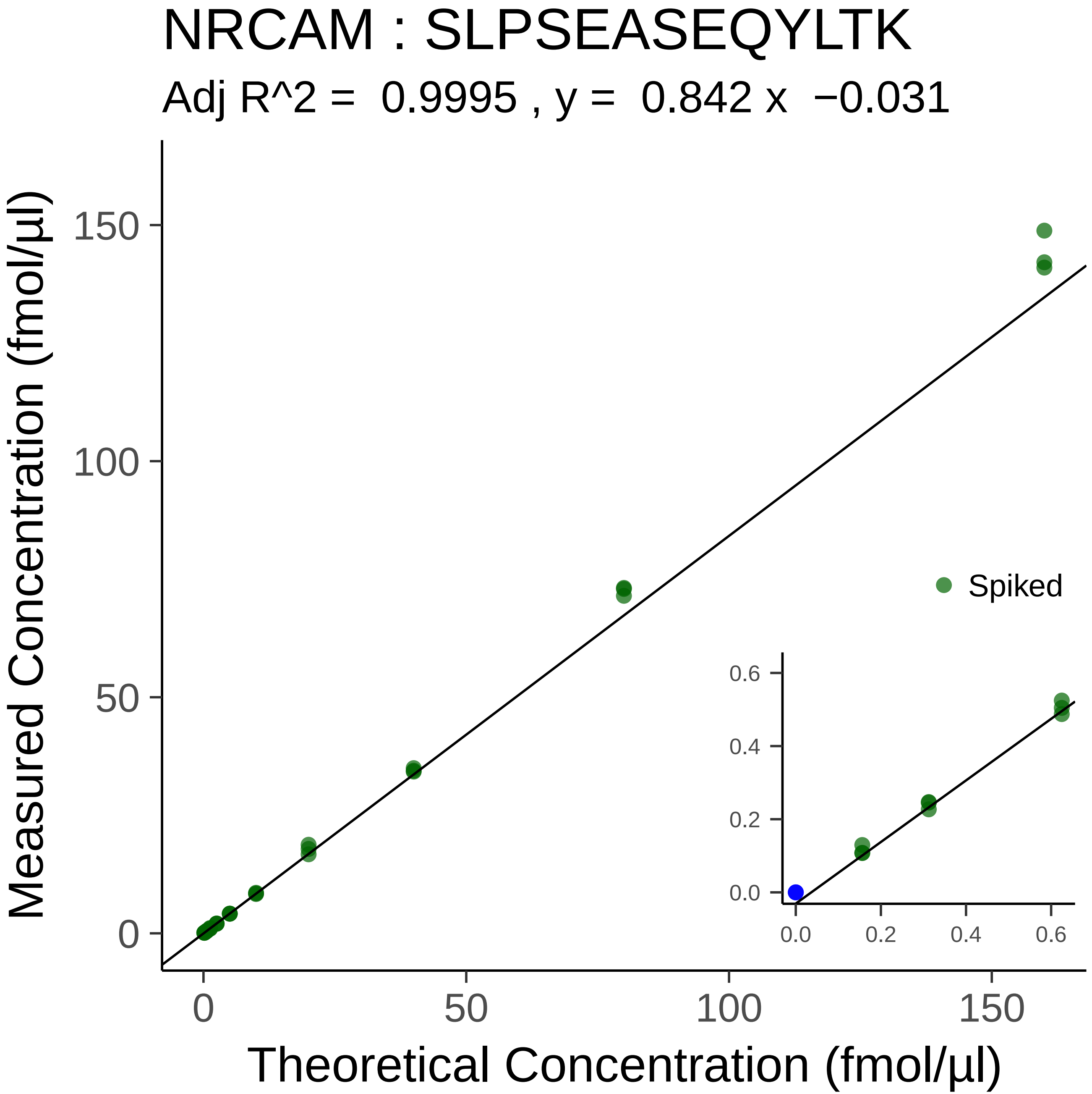

NRCAM : SLPSEASEQYLTK
Adj R^2 = 0.9995 , y = 0.842 x −0.031
Measured Concentration (fmol/µl)
Theoretical Concentration (fmol/µl)
0
50
100
150
Spiked
0.0
0.2
0.4
0.6

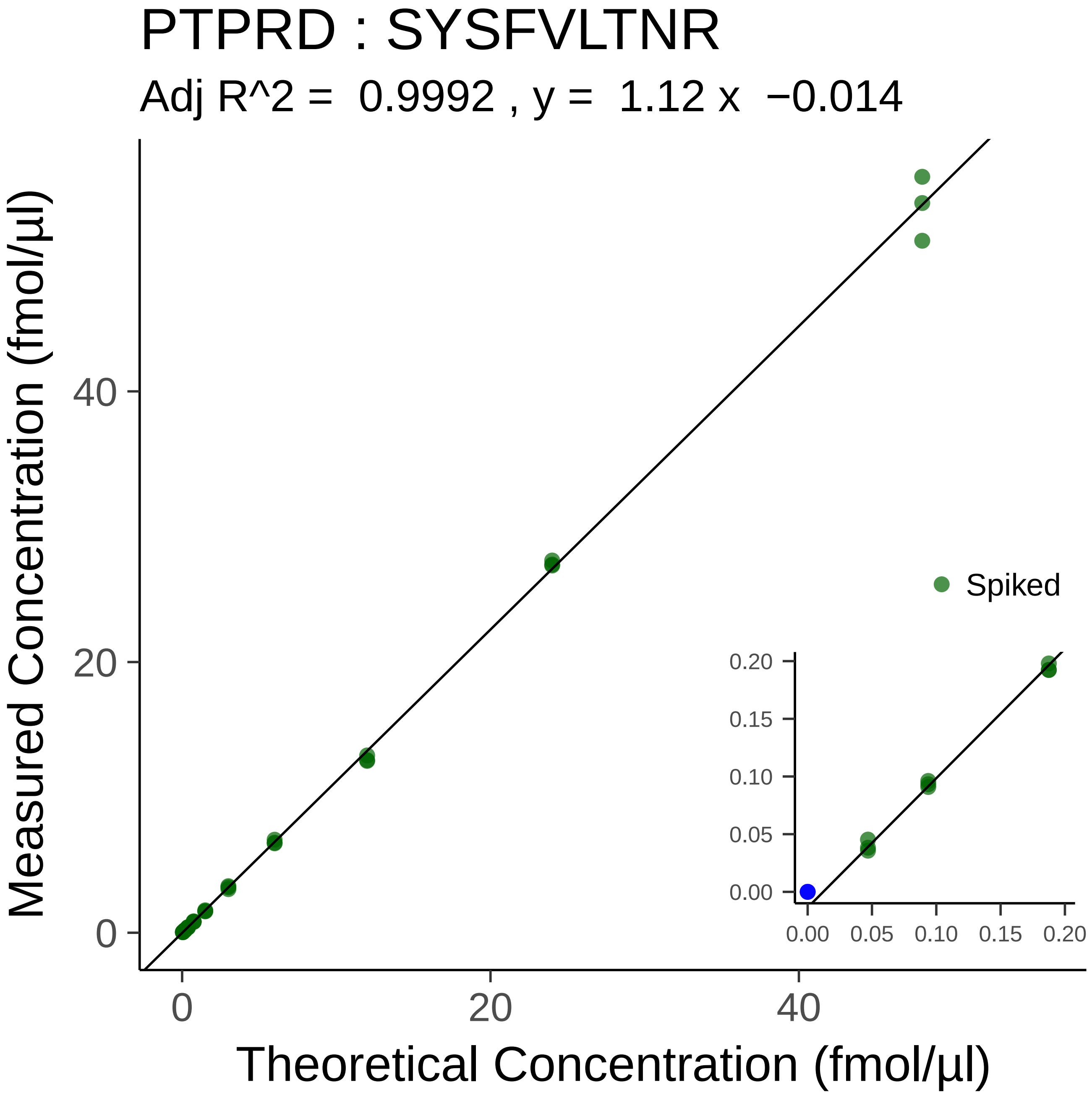

PTPRD : SYSFVLTNR
Adj R^2 = 0.9992 , y = 1.12 x −0.014
Measured Concentration (fmol/µl)
Theoretical Concentration (fmol/µl)
0
20
40
Spiked
0.00
0.05
0.10
0.15
0.20

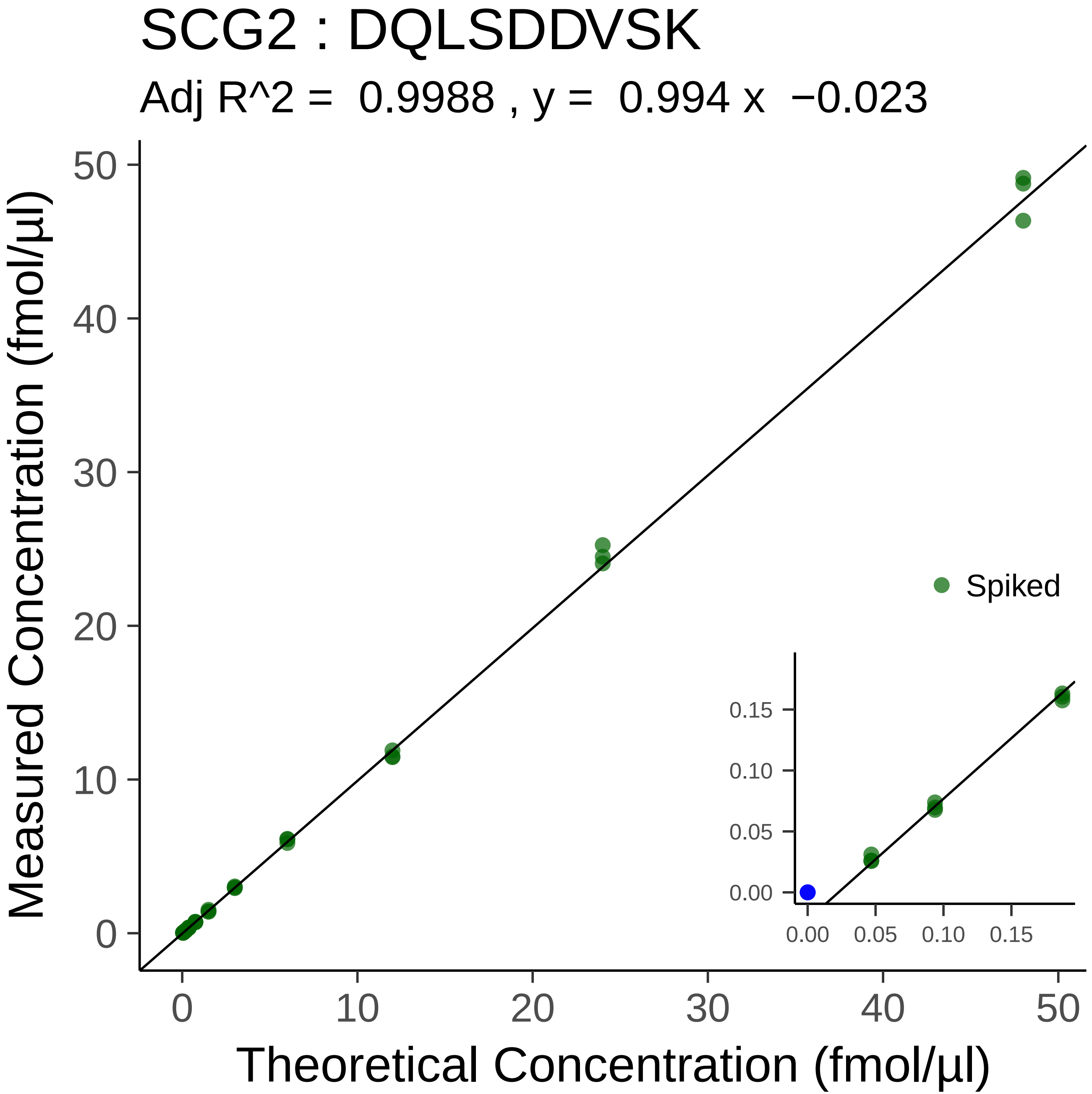

SCG2 : DQLSDDVSK
Adj R^2 = 0.9988 , y = 0.994 x −0.023
Measured Concentration (fmol/µl)
Theoretical Concentration (fmol/µl)
0
10
20
30
40
50
0
10
20
30
40
50
Spiked
0.00
0.05
0.10
0.15
0.00
0.05
0.10
0.15

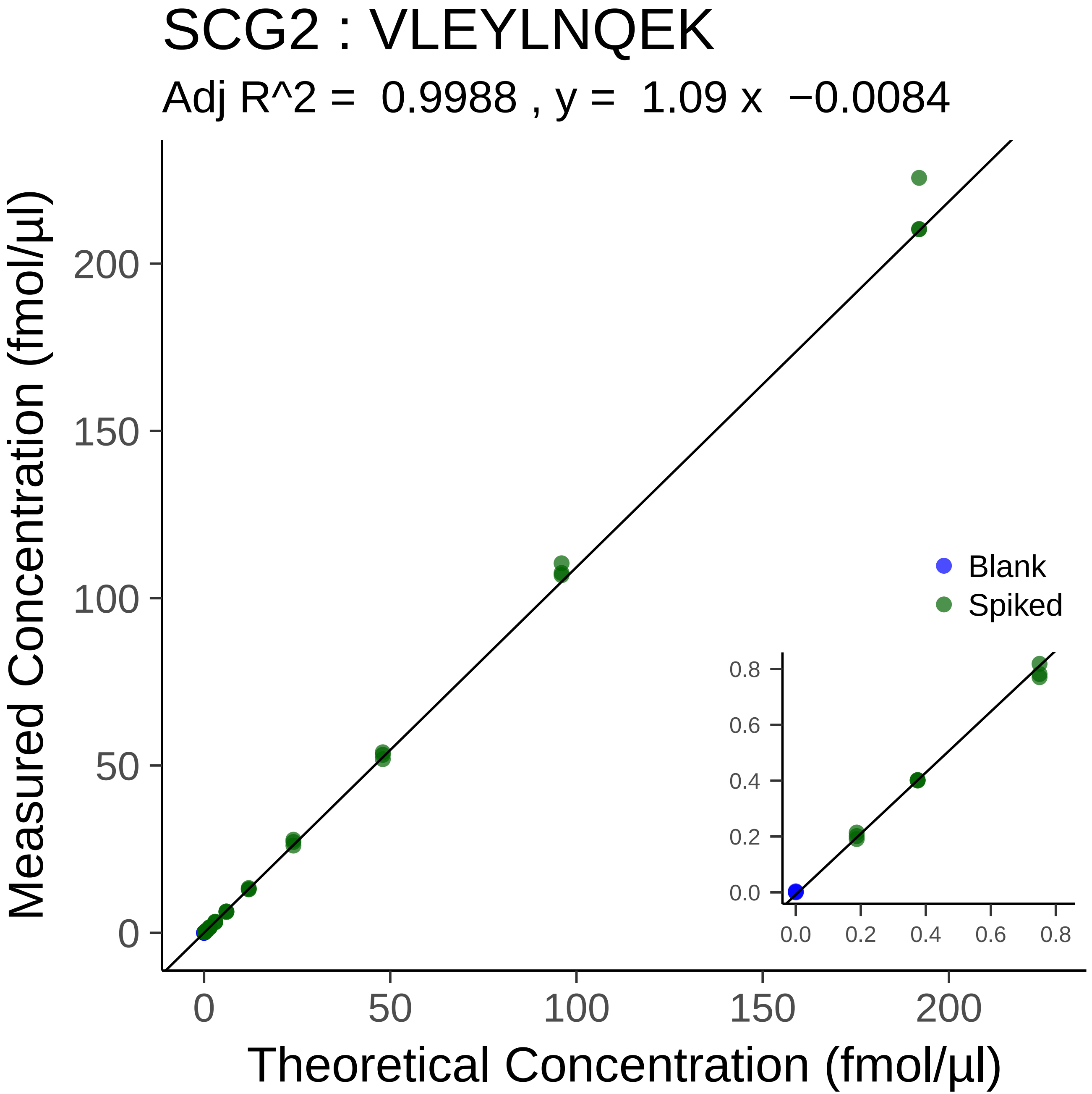
SCG2 : VLEYLNQEK
Adj R^2 = 0.9988 , y = 1.09 x −0.0084
Measured Concentration (fmol/µl)
Theoretical Concentration (fmol/µl)
0
50
100
150
200
Blank
Spiked
0.0
0.2
0.4
0.6
0.8

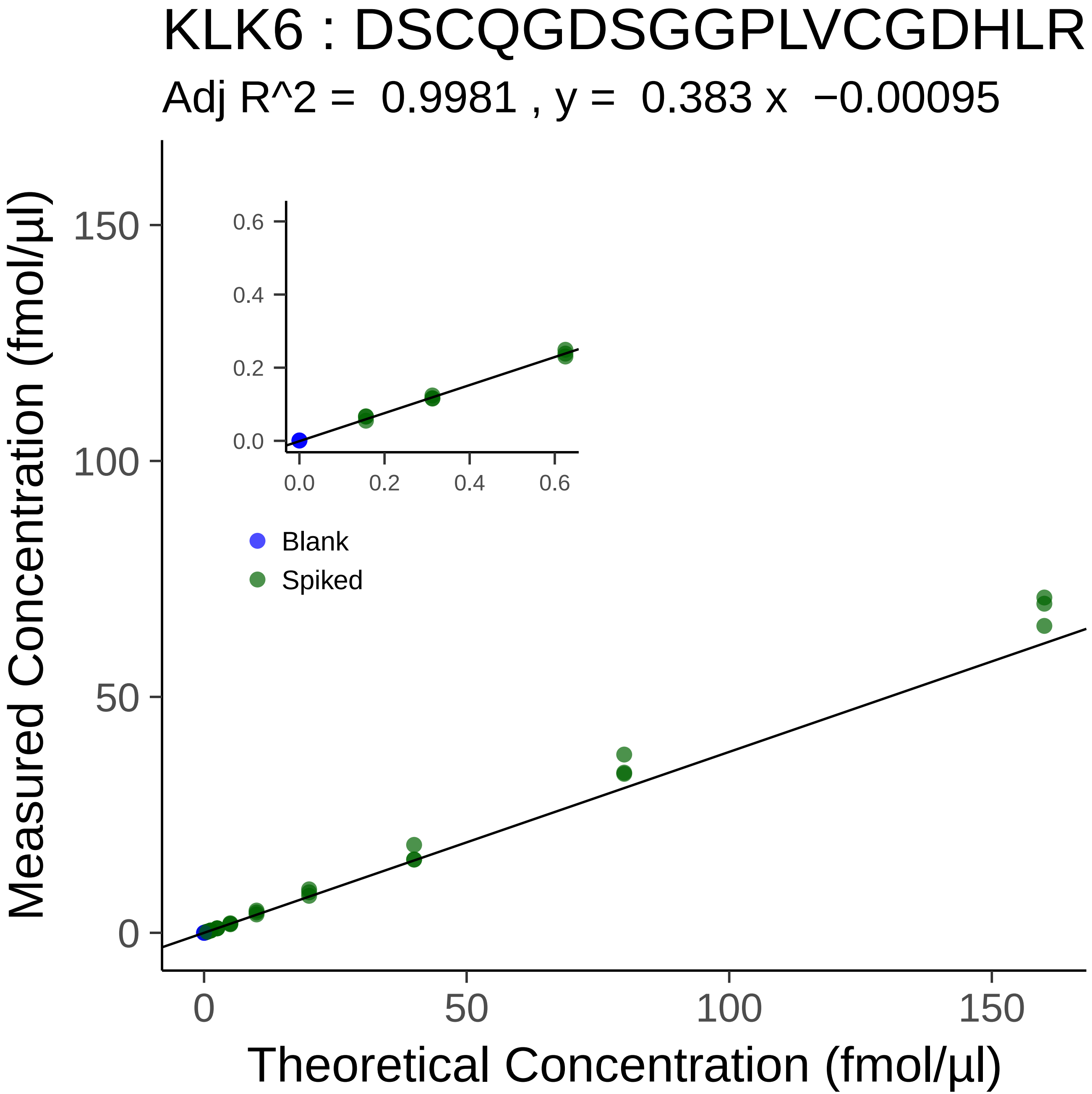
KLK6 : DSCQGDSGGPLVCGDHLR
Adj R^2 = 0.9981 , y = 0.383 x −0.00095
Measured Concentration (fmol/µl)
Theoretical Concentration (fmol/µl)
0
50
100
150
0
50
100
150
0.0
0.2
0.4
0.6
0.0
0.2
0.4
0.6
Blank
Spiked
