# Supplementary material for: Development of robust targeted proteomics assays for cerebrospinal fluid biomarkers in multiple sclerosis
Source: Clin Proteomics. 2020 Sep 18;17:33. doi: 10.1186/s12014-020-09296-5 (PMC7499868; doi:10.1186/s12014-020-09296-5)
Supplement: Supplementary file 13 — Additional file 13: Fig. S4. Representative transition peaks from the Skyline analysis. A and B show typical examples of used transitions. The transition intensity, integration limits, retention time and mass error (ppm) is illustrated. C and D show examples of how 1-3 transitions were often much higher than the rest. Peak smoothing (Savitzky-Golay) was used in Skyline, which notably does not affect the quantification. [file 12014_2020_9296_MOESM13_ESM.pdf]

Supplementary Figure 4

A

Amyloid-like protein 1
WEPDPQ**R**

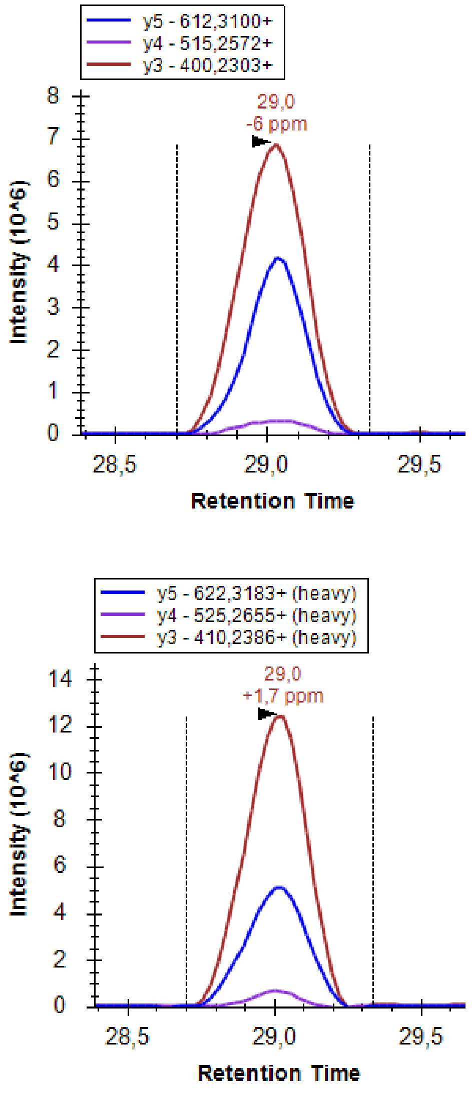

B

Cadherin-13
YEVSSPYF**K**

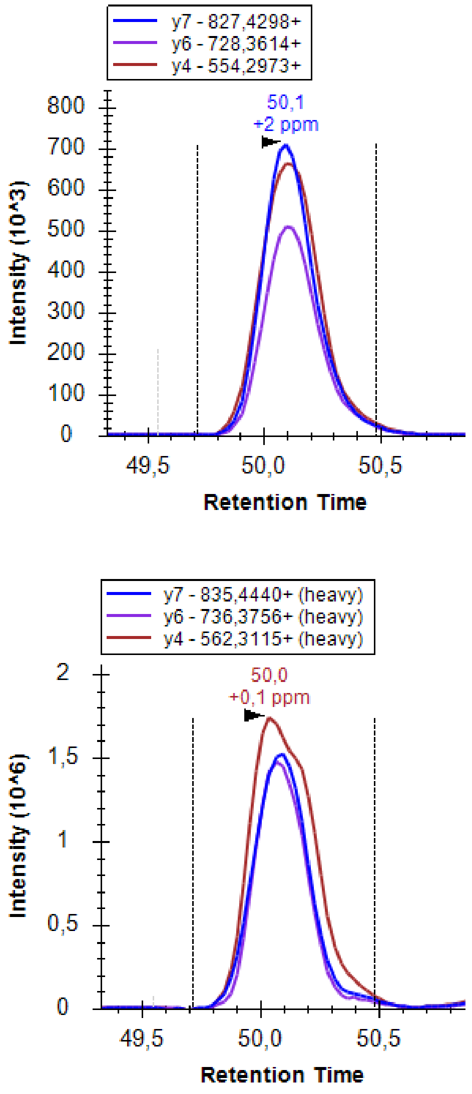

C

Neuronal cell adhesion molecule
VFNTPEGVPSAPSSLK

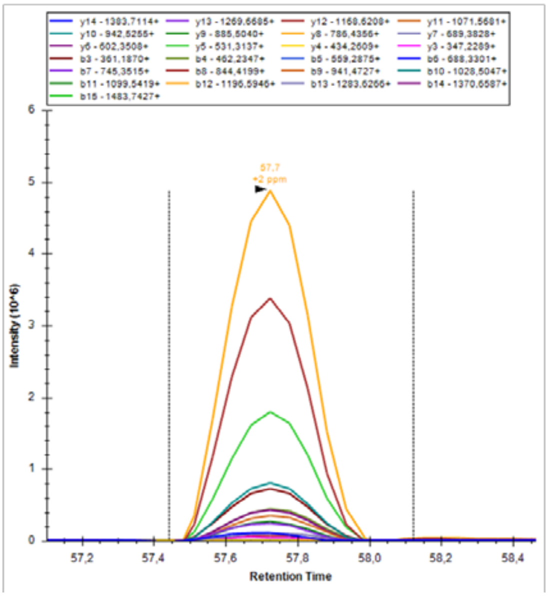

D

Amyloid-like protein 1
WEPDPQR

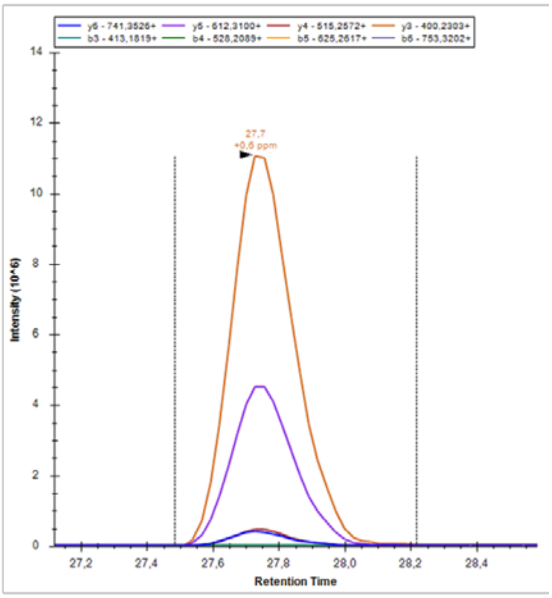

Supplementary Figure 4: Representative transition peaks from the Skyline analysis. **A** and **B** show typical examples of used transitions. The transition intensity, integration limits, retention time and mass error (ppm) is illustrated. **C** and **D** show examples of how 1-3 transitions were often much higher than the rest. Peak smoothing (Savitzky-Golay) was used in Skyline, which notably does not affect the quantification.
